# Isolation and Structure Elucidation of Cembranoids from a Dongsha Atoll Soft Coral *Sarcophyton stellatum*

**DOI:** 10.3390/md16060210

**Published:** 2018-06-14

**Authors:** Atallah F. Ahmed, Yi-Wei Chen, Chiung-Yao Huang, Yen-Ju Tseng, Chi-Chen Lin, Chang-Feng Dai, Yang-Chang Wu, Jyh-Horng Sheu

**Affiliations:** 1 Department of Marine Biotechnology and Resources, National Sun Yat-sen University, Kaohsiung 804, Taiwan; afahmed@KSU.EDU.SA (A.F.A.); m985020005@student.nsysu.edu.tw (Y.-W.C.); huangcy@mail.nsysu.edu.tw (C.-Y.H.); d935020003@student.nsysu.edu.tw (Y.-J.T.); 2 Department of Pharmacognosy, College of Pharmacy, King Saud University, Riyadh 11451, Saudi Arabia; 3 Institute of Biomedical Sciences, National Chung Hsing University, Taichung 402, Taiwan; lincc@dragon.nchu.edu.tw; 4 Institute of Oceanography, National Taiwan University, Taipei 112, Taiwan; corallab@ntu.edu.tw; 5 Graduate Institute of Natural Products, Kaohsiung Medical University, Kaohsiung 807, Taiwan; yachwu@kmu.edu.tw; 6 Frontier Center for Ocean Science and Technology, National Sun Yat-sen University, Kaohsiung 804, Taiwan; 7 Department of Medical Research, China Medical University Hospital, China Medical University, Taichung 404, Taiwan

**Keywords:** soft coral, *Sarcophyton stellatum*, cembranoid, cytotoxic activity, anti-inflammatory activity

## Abstract

Six new polyoxygenated cembrane-based diterpenoids, stellatumolides A–C (**1**–**3**), stellatumonins A and B (**4** and **5**), and stellatumonone (**6**), were isolated together with ten known related compounds (**7**–**16**) from the ethyl acetate (EtOAc) extract of soft coral *Sarcophyton stellatum*. The structures of the new compounds were established by extensive spectroscopic analyses, including 1D and 2D nuclear magnetic resonance (NMR) spectroscopy and data comparison with related structures. Compounds **8** and **14** were isolated from a natural source for the first time. The isolated metabolites were shown to be not cytotoxic against a limited panel of cancer cells. Compound **9** showed anti-inflammatory activity by reducing the expression of proinflammatory cyclooxygenase-2 (COX-2) and inducible nitric oxide synthase (iNOS) proteins in lipopolysaccharide (LPS)-stimulated mouse leukaemic monocyte macrophage (RAW 264.7) cells.

## 1. Introduction

Soft corals including those of the genus *Sarcophyton* have been well recognized to be a rich source of structurally unique and bioactive diterpenes, in particular cembranoids [1]. Since the first report of sarcophine, a cembrane with a α,β-unsaturated γ-lactone ring [2], series of cembrane-derived terpenoids, such as cembranes with *trans-* [3,4] or *cis-* [4,5] fused α-methylene-γ-lactone rings or with the same γ-lactone ring as in sarcophine [6,7,8] have been discovered from the worldwide investigation of *Sarcophyton* species. Furthermore, some structurally complex biscembranes derived from Diels–Alder reaction also have been unveiled [9,10,11]. Recently, many cembranoids possessing new structures have been isolated from soft corals [12,13,14,15,16,17,18,19,20]. Accordingly, more cembranoids with promising bioactivities have been reported [21,22,23,24,25,26]. Thus, further investigation on new cembranoids and bioactivities might warrant further biomedical research. As a recent publication reported the isolation of four cembranes possessing only simple functional groups from *Sarcophyton stellatum* [27], we further investigated the secondary metabolites of the same coral collected from Dongsha Atoll in order to search for bioactive and new metabolites for further medicinal study. This investigation led to the discovery of six new cembranoid diterpenes: stellatumolides A–C (**1**–**3**), stellatumonins A and B (**4** and **5**), and stellatumonone (**6**), along with ten known related compounds (**7**–**16**). The assay for in vitro anti-inflammatory activity of the isolated compounds showed that (+)-sarcophine (**9**) could reduce the accumulation of the proinflammatory cyclooxygenase-2 (COX-2) and inducible nitric oxide synthase (iNOS) proteins.

## 2. Results and Discussion

The ethyl acetate (EtOAc) extract of *S. stellatum* was initially fractionated over a silica gel column, and the obtained fractions were then separated and purified by repeated column chromatography to yield new cembranoids **1**–**6** (Figure 1), and structures were elucidated on the basis of spectroscopic analyses (Supplementary Materials, Figures S1–S47).

Stellatumolide A (**1**), [α]${}_{D}^{25}$ + 57.2, (*c* 0.1, CHCl_3_), was isolated as a colorless oil and exhibited a sodium adduct ion peak at *m/z* 371.1832 [M + Na]^+^ from high-resolution electrospray ionization mass spectrometry (HRESIMS). Thus, the molecular formula C_20_H_28_O_5_ was established. The infrared (IR) absorption band at ν_max_ 3445 cm^−1^ and the ion peak appearing in the ESIMS at *m/z* 353 [M − H_2_O + Na]^+^ indicated the presence of one hydroxy group in the molecule. The 20 carbon signals in the ^13^C nuclear magnetic resonance (NMR) spectrum (Table 1) were attributable to four methyls, six methylenes, three methines (including two oxy- and one olefinic CH), and seven nonprotonated carbons (including three olefinic, three *sp^3^* oxygenated carbons, and one carbonyl carbon). The α,β-unsaturated γ-lactone moiety was deduced from NMR signals at *δ*_C_ 171.1, 157.9, and 128.8 (each C), and IR absorption at ν_max_ 1760 and 1680 cm^−1^. Moreover, one trisubstituted double bond was found by NMR signals at *δ*_C_ 130.6 (C), 129.2 (CH), and *δ*_H_ 5.26 (1H, d, *J* = 11.0 Hz) (Table 2). The correlation spectroscopy (COSY) spectrum of **1** revealed the presence of three consecutive spin systems (Figure 2). The heteronuclear multiple bond correlations (HMBC) from H_3_-17 to the carbonyl carbon (*δ*_C_ 171.1, C), C-1 (*δ*_C_ 157.9, C), and C-15 (*δ*_C_ 128.8, C); H_3_-18 to C-3, C-4, and C-5; H_3_-19 to C-7, C-8, and C-9; and H_3_-20 to C-11, C-12, and C-13 established the carbon skeleton of **1**. The unresolved 2H signal (*δ*_H_ 4.27) present in the ^1^H NMR spectrum, measured in CDCl_3_, could be well resolved by measuring **1** in C_6_D_6_ (Experimental Section), resulting in two signals at *δ*_H_ 4.19 (1H, s) and 3.93 (1H, d, *J* = 10.0 Hz), which were further assigned to oxymethines H-3 and H-7, respectively. Therefore, the HMBC correlation from H-3 to C-8 clearly indicated an ether linkage between C-3 and C-8 (Figure 2). In consideration of the seven degrees of unsaturation and the molecular formula of **1**, an additional ether linkage was placed between the ketal carbon C-2 and C-4. By further comparison of the ^13^C NMR spectroscopic data of **1** with those of a spiro oxetanebutenolide derivative ramariolide B [28], two signals of nonprotonated *sp^3^* oxycarbons resonating at *δ*_C_ 113.4 and 92.2 for C-2 and C-4 of **1**, relative to those at *δ*_C_ 112.7 and 90.7 for corresponding carbons, established an unusual spiroketal unit between the oxetane (C-2/C-4) and the γ-lactone ring (C-2/C-16) of **1**. From the above findings, detailed HMBC spectrum (Figure 3), and 2D NMR correlation analysis (Figure 2), the planar structure of **1** was established.

**Figure 1:**
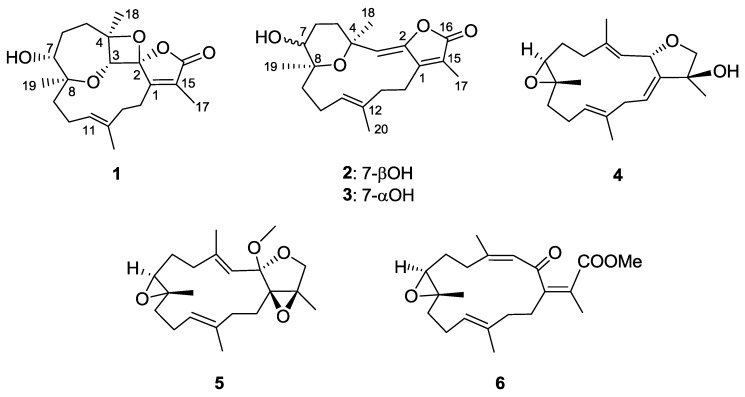
Structures of compounds **1**–**6**.

The relative configurations at C-2, C-3, C-4, C-7, and C-8 of **1** were proposed from analysis of nuclear Overhauser effect spectroscopy (NOESY) (Figure 4, molecular structures are energy-minimized using MM2 force field method). A strong NOE interaction of H-3 (*δ*_H_ 4.19, s) with H-7 (*δ*_H_ 3.93, br d, *J* = 10.0 Hz), measured in C_6_D_6_ (Figure 3 and Figure 4), indicated that both protons should be positioned on the same face and were arbitrarily assigned as β-oriented. Thus, the significant NOE correlation of H-3 with one of the H_2_-14 (*δ*_H_ 2.12, dd, *J =* 13.0, 13.0 Hz) established the β-orientation of the C-1 residue in the oxetane, and hence the R* configuration of C-2. Furthermore, one of the methylene protons at C-6 (*δ*_H_ 1.22, m) exhibited a NOESY correlation with H-7, and was therefore characterized as H-6β, while the other (*δ*_H_ 1.50, m) was assigned as H-6α. H-6α showed NOE interactions with both H_3_-18 and H_3_-19; therefore, H_3_-18 and H_3_-19 were also positioned on the α-face. Due to the high flexibility of the macrocycle ring structure of **1**, a conformational search at the molecular mechanics level was carried out using CONFLEX^®^ [29,30] software (CONFLEX 7.0, Conflex Corp., Tokyo, Japan) with the MMFF94s force field. As a result, a total of 18 conformers were determined. Three of these conformers were found to be the most populated (Figure 5) and fulfilled the observed NOESY correlations (Figure 3 and Figure 4). In these conformers, the splitting pattern (br d) and the coupling constant (*J* value = 10.0 Hz) displayed by H-7 in **1** could be thus explained as a function of the dihedral angles 179.7° and 66.2° formed with the adjacent 6-CH_2_ protons (H*_trans_* and H*_cis_*; *J* = 10–15 and 0–2 Hz, respectively). Finally, the *Z* and *E* geometries of the C-1/C-15 and C-11/C-12 double bonds in **1**, respectively, were established by the NOE interaction of H_3_-17 (*δ*_H_ 1.49, s) with H-14 (*δ*_H_ 2.01, ddd, *J* = 13.0, 8.0, 8.0 Hz) and the upfield shift of C-20 (*δ*_C_ 16.5 in CDCl_3_), respectively. The above results revealed that **1** possesses a structure in good agreement with that shown in formula **1** (Figure 1). Compound **1** was found to be the first cembranoid possessing an unusual 6,9,2′-trioxaspiro[bicycle(5,2,0)nonane-8,1′- cyclopentane]-3′-one moiety in the molecule.

**Figure 2:**
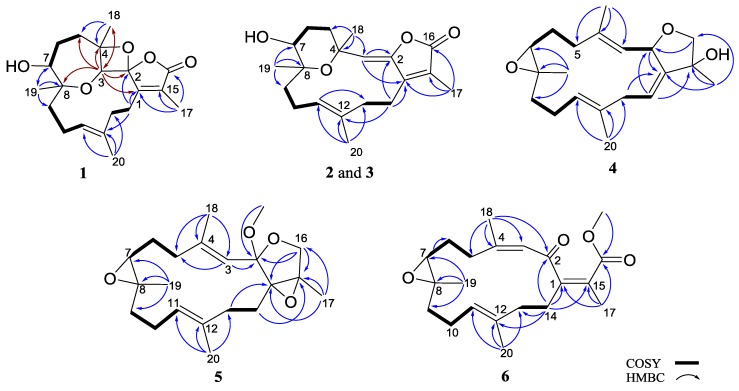
Selected COSY and HMBC correlations of **1**–**6**.

**Figure 3:**
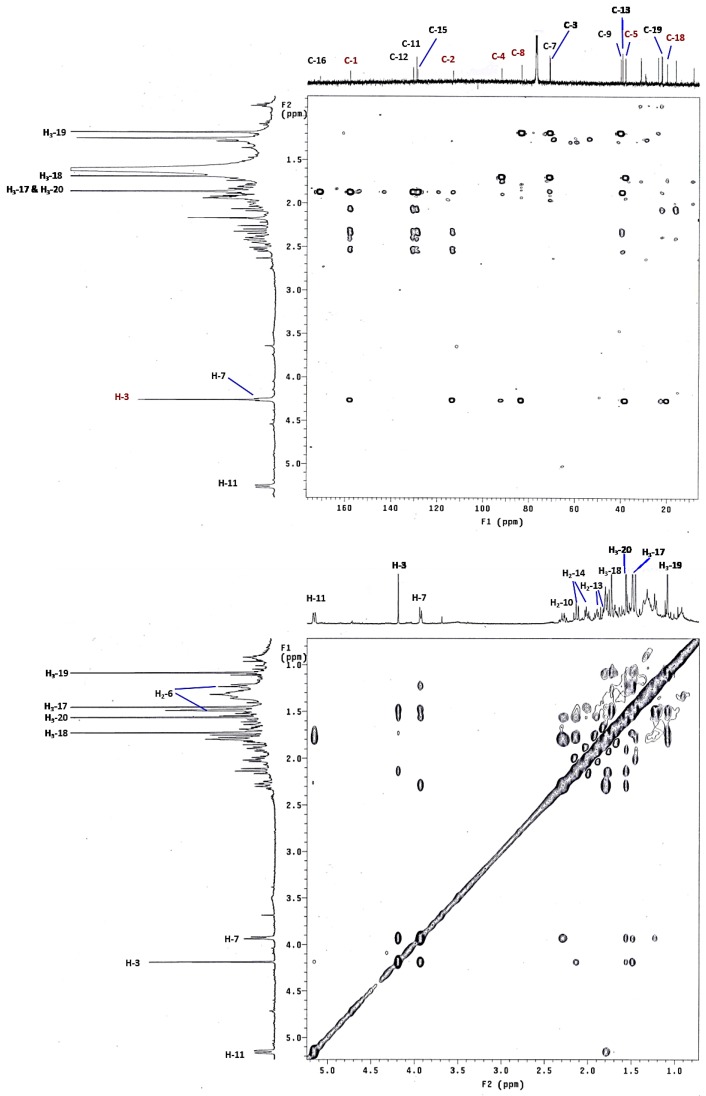
HMBC (**upper**) in CDCl_3_ and NOESY (**lower**) spectra of **1** in C_6_D_6_.

Stellatumolide B (**2**), [α]${}_{D}^{25}$ −59.4 (*c* 0.3, CHCl_3_), possessed the molecular formula C_20_H_28_O_4_ as indicated by HREIMS at *m/z* 332.1985, implying seven degrees of unsaturation. Similarly to compound **1**, the strong IR absorptions at ν_max_ 3420, 1752, and 1666 cm^–1^ indicated the presence of hydroxyl, ester carbonyl, and olefinic groups, respectively. The carbonyl group was identified as an α,β-unsaturated ester from the ^13^C NMR signals of three nonprotonated carbons at *δ*_C_ 170.3, 152.2, and 123.3. Moreover, two trisubstituted double bonds were deduced from NMR signals at *δ*_C_/*δ*_H_ 147.8 (C), 130.9 (C), 117.4 (CH)/5.16 (1H, s), and 129.1(CH)/ 4.97 (1H, dd) (Table 1 and Table 2). The ^13^C NMR spectrum, in addition to distortionless enhancement by polarization transfer (DEPT) and heteronuclear multiple quantum coherence (HMQC) experiments, showed 20 carbon signals, assigned to four methyls, six methylenes, one *sp^3^* oxymethine, two olefinic methines, and seven quaternary carbons (including four olefinic *sp^2^*, two oxygenated *sp^3^*, and one carbonyl carbons). As for compound **1**, three partial structures of consecutive spin systems were distinguished by analysis of COSY correlations (Figure 2). Examination of the HMBC correlations of **2** enabled connection of these three partial structures, including the positioning of hydroxyl groups at C-7 and an ether linkage at C-4 and C-8 (Figure 2). From the HMBC correlations detected from H_3_-18 (*δ*_H_ 1.48, s) to C-3 (*δ*_C_ 117.4, CH) and H-3 (*δ*_H_ 5.16, s) to C-2 (*δ*_C_ 147.8, C) and C-1 (*δ*_C_ 152.3, C), the C-2/C-3 location of the oxytrisubstituted double bond could be assigned. Furthermore, the HMBC correlations from H_3_-17 (*δ*_C_ 1.94, s) to C-1, C-15 (*δ*_C_ 123.3, C), and the carbonyl C-16 (*δ*_C_ 170.3, C), confirmed the C-1/C-2 location of the γ-lactone ring. In considering the degrees of unsaturation and molecular formula, an ether linkage was placed between C-4 and C-8.

**Figure 4:**
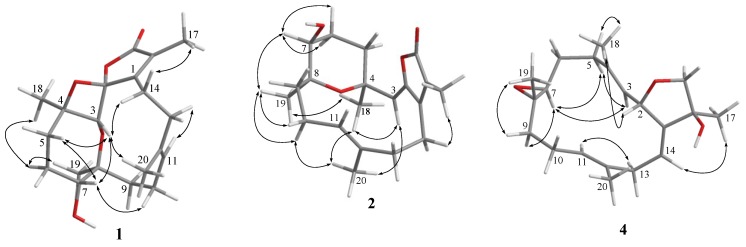

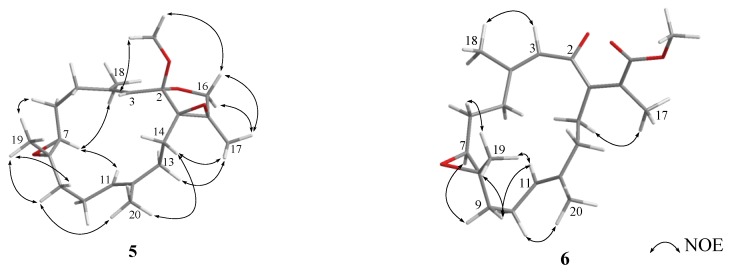
Selected nuclear Overhauser effect (NOE) correlations for **1**, **2**, and **4**–**6.**

**Figure 5:**
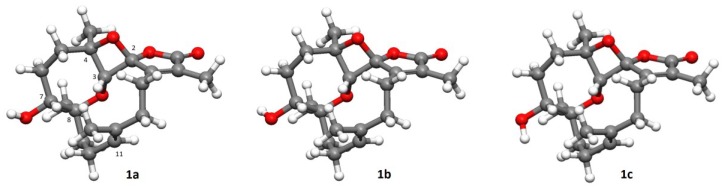
CONFLEX/MMFF94s-optimized most abundant conformers of compound **1** and their relative abundance: (**1a**) 85.86 kcal/mol (54.702%); (**1b**) 86.17 kcal/mol (32.668%); (**1c**) 86.74 kcal/mol (12.576%).

Further detailed examination of 2D NMR correlations (Figure 2) established the gross structure of **2** as 4,8-epoxy-7β-hydroxy-cembra-1(15),2,11-trien-16,2-olide. Careful investigation of NOESY correlations in combination with measuring distances between relevant protons in the MM2 energy-minimized model enabled resolution of the relative configuration of compound **2** (Figure 4). The NOE interactions of H_3_-18 with H_3_-19 and H_3_-20, H_3_-20 with one of the methylene protons at C-10 (*δ*_H_ 1.82, dd, *J* = 14.5), H_2_-10 with H_3_-19, and H_3_-19 with H-7 (*δ*_H_ 3.44 dd, *J* = 7.0, 5.0) revealed that the methyl groups at C-4 and C-8 were on the same face as H-7, and assuming that H_3_-18 was α-oriented, both C-7 and C-8 were therefore of the S* configuration. Moreover, the NOE correlations displayed by H_3_-17/H-14, H_3_-18/H-3, and H_3_-20/H-10 assigned the geometry of the double bonds at C-1/C-15, C-2/C-3, and C-11/C-12 as *Z*, *Z*, and *E*, respectively. On the basis of the above findings, the structure of stellatumolide B (**2**) was elucidated. 

Stellatumolide C (**3**) was found to have the same molecular formula (C_20_H_28_O_4_) as compound **2** and the same hydroxyl and unsaturated γ-lactone functionalities on the basis of its HREIMS and IR spectra. The NMR data of **3** (Table 1 and Table 2) were found to be mostly identical to those of **2**, with the exception that the chemical signal of C-7 was found to be shifted upfield (Δ*δ*_C_ − 2.7 ppm) in comparison with that of **2**, and the variational *J* values of 9.5 and 5.0 Hz at H-7 of **3** relative to that of **2** (7.0 and 3.0 Hz). After interpretation of the 2D NMR spectra, in particular the NOE correlations including that of H_3_-18/H_3_-19, compound **3** was established as the 7-epimer of compound **2**.

**Figure 6:**
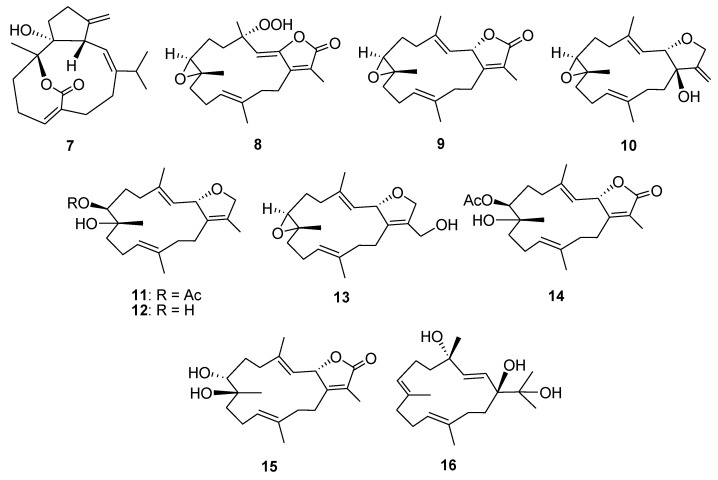
Structures of known compounds **7**–**16**.

**Figure 7:**
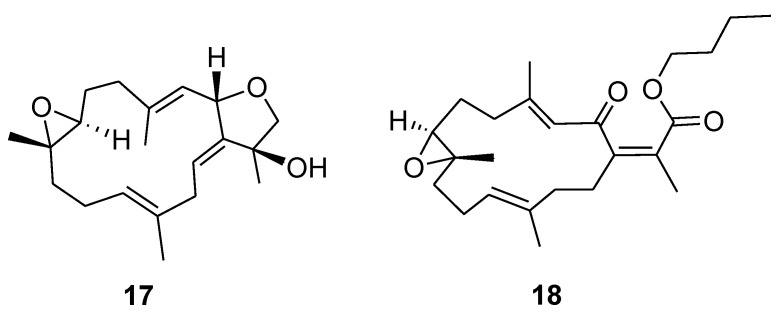
Known natural products, sarcophyton B (**17**) and secosarcophinolide (**18**), used for spectral data comparison.

Stellatumonin A (**4**) was isolated as a colorless oil ([α]${}_{D}^{25}$ −220, *c* 0.3, CHCl_3_) and possessed the molecular formula C_20_H_30_O_3_, as determined by HRESIMS (*m/z* 341.2091, [M + Na]^+^). The NMR spectroscopic features (Table 1 and Table 2) indicated that **4** was structurally similar to **9** (Figure 6) [2,7] from C-2 to C-12 in the fourteen-membered ring; however, an additional 1,14-double bond was observed. The planar structure of **4** was obtained by detailed investigation and interpretation of 2D NMR (COSY, HMQC, and HMBC) spectroscopic correlations (Figure 2), and was determined to be 2,16:7,8-diepoxy-cembra-1(14),3,11-trien-15-ol. After elucidation of the structure of **4**, a natural marine compound **17** (Figure 7) [31] was found to have the very similar structure. However, on comparison of the NMR data of both compounds, significant upfield shifts at C-2, C-3, C-12, C-14, C-16, and C-17 (Δ*δ*_C_ −3.5, −3.3, −2.2, −2.9, −1.5, and −4.0 ppm, respectively), and a downfield shift at C-13 (Δ*δ*_C_ + 3.8 ppm), in **4** relative to **17** were observed. Also, H-14 in **4** exhibited a significant downfield shift (Δ*δ*_H_ − 0.46 ppm) relative to that of **17**. The *E* geometry, instead of *Z* geometry as in **17**, for the C-14/C-1 double bond was thus suggested for **4**, and was consequently confirmed by the NOE interaction of H-14 with H_3_-17. Furthermore, detailed NOE correlation analysis of **4** (Figure 4) confirmed the relative configuration at chiral carbons C-2, C-7, C-8, and C-15 to be 2S*, 7S*, 8S*, and 15S*, respectively (Figure 1).

The HRESIMS (*m/z* 348.2301, M^+^) and NMR data (Table 1 and Table 2) established the molecular formula of stellatumonin B (**5**) to be C_21_H_32_O_4_, with six degrees of unsaturation. The ^13^C NMR of **5** displayed 21 carbon signals ascribable to five methyls (including that of a methoxy group), seven methylenes, three methines, and six quaternary carbons. In addition, a trisubstituted epoxide (*δ*_C_/*δ*_H_ 62.0/2.84 and 60.7), a tetrasubstituted epoxide (*δ*_C_ 71.3 and 63.3, each C), two trisubstituted double bonds (*δ*_C_/*δ*_H_ 142.8, C; 134.6, C; 124.6, CH/5.24; and 120.6, CH/5.21), a quaternary dioxycarbon (*δ*_C_ 107.4, C), and an oxymethylene (*δ*_C_/*δ*_H_ H 68.8, CH_2_/3.64, and 3.88) were disclosed. The COSY and HMBC correlations, as depicted in Figure 2, determined the positions of the two trisubstituted double bonds, the two epoxides, and the oxymethylene groups at C-3/C-4 and C-11/C-12, C-7/C-8 and C-1/C-15, and C-16, respectively, inferring the tetracyclic structure of **5** (Figure 2). Furthermore, C-2 was determined to be the ketal carbon (*δ*_C_ 107.4), containing a methoxy group (*δ*_C_ 49.3, CH_3_), as supported by the HMBC correlation from protons of methoxy group to C-2. The gross structure of **5** was thus confirmed. The upfield chemical shifts (*δ*_C_ < 20 ppm) of C-18 and C-20 and NOE correlations (Figure 4) proved the *E* configuration of C-3/C-4 and C-11/C-12 double bonds of **5**. One of the H_2_-16 protons resonating at *δ*_H_ 3.88 was arbitrarily designated as H-16β, while the other as H-16α (*δ*_H_ 3.64). The NOE interactions of H-16β with 2-OCH_3_ protons, and H_3_-17 with H_2_-16, H-13, (*δ*_H_ 2.10) and H-14 (*δ*_H_ 1.82) reflected the β-orientations of the methoxy at C-2 and the oxygen of the epoxide at C-1/C-15. The significant NOE response of the olefinic H-3 with H-7 and other NOE correlations (Figure 4) in **5** established the *trans* geometry of the 7.8-epoxide with an α-oriented H-7 and β-oriented H_3_-19. Thus, the relative configuration of compound **5** was determined.

The NMR spectroscopic data of **6** (Table 1) are similar to those of secosarcophinolide (**18**) [32], with the difference that the *n*-butoxy group at C-16 was replaced by a methoxy group in **6**. COSY and HMBC correlations further confirmed the molecular skeleton of **6** (Figure 2). Analysis of the NOE corrections (Figure 4) established *Z* geometry for 1,15- and 3,4-double bonds, and *E* geometry for the 11,12-double bond and *trans*-7,8-epoxide of **6**. Thus, the relative configuration of **6** was determined. This result could be further supported by NMR data of biscembranoids containing the same *cis*-β-methyl-α,β-conjugated enone [11,33].

**Scheme 1:**
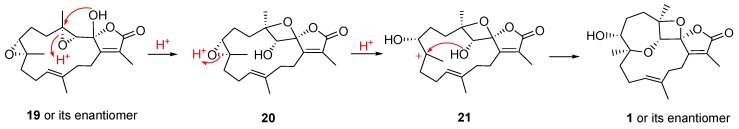
Proposed biosynthetic pathway for **1.**

A plausible biosynthethic pathway of **1** from the possible precursor **19** was proposed as shown in Scheme 1. The acid-catalyzed ring opening of the 3,4-epoxide of a postulated diepoxy intermediate **19** followed by the nucleophilic addition of the 2-hydroxy group at the cationic carbon C-4 led to the dioxaspiro intermediate **20**. Acid-catalyzed ring opening of 7,8-epoxide affords a carbonium ion intermediate **21** which after the nucleophilic attack of 3-hydroxy group at the cationic carbon C-8 from an upward orientation gives **1**. 

In addition to the isolated new compounds (**1**–**6**), ten known cembrenoids (**7**–**16**) (Figure 6) were also obtained from *S. stellatum*, including sarsolilide (**7**) [34,35], a hydroperoxide (**8**) obtained by autoxidation of dihydrofuranocembrenoid [36] (+)-sarcophine (**9**) [2,7], laevigatol B (**10**) [37], sarcophytonin E (**11**) [38], sarcophytonin C (**12**) [39], 17-hydroxysarcophytoxide (**13**) [40,41], 7β-acetoxy-8α-hydroxydeepoxysarcophine (**14**) [42], 7β,8α-dihydroxydeepoxy-*ent*-sarcophine (**15**) [43], and crassumol A (**16***)* [38]. Among these cembranoids, compounds **8** and **14** were isolated from a natural source for the first time.

Cytotoxicities of metabolites **1**–**16** against the growth of human hepatocellular liver carcinoma (HepG2), human breast cancer (MDA-MB231), and human lung adenocarcinoma (A549) cell lines were screened. None of the metabolites exhibited inhibitory activity against the growth of the tested cancer cells (IC_50_ > 20 μg/mL).

**Figure 8:**
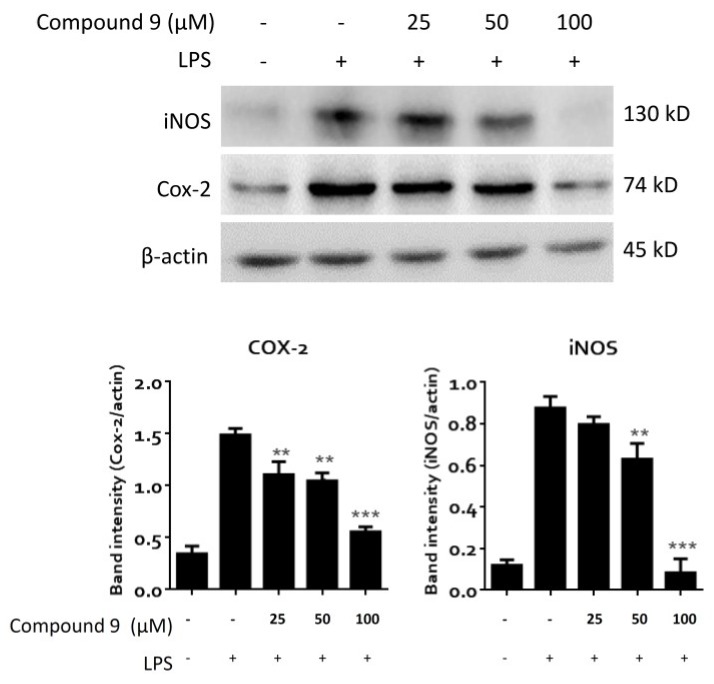
The effect of compound **9** on the protein expression of cyclooxygenase-2 (COX-2) and inducible nitric oxide synthase (iNOS) in mouse leukaemic monocyte macrophage (RAW 264.7) cells. Cells were pretreated with compound **9** at a concentration of 25, 50, and 100 μM for 1 h, and then treated with 1 μg/mL lipopolysaccharide (LPS) for 24 h. Band image and the densitometric quantifications of Western blotting results is shown (**upper**). β-actin was used as the internal control protein. The band intensity was quantitated using ImageJ software. The bar graph (**lower**) shows the mean ± SD values from three independent experimental results. ** *p* < 0.01; *** *p* < 0.001 compared with LPS-alone group.

Furthermore, the in vitro anti-inflammatory activity of (+)-sarcophine (**9**) on inhibition of the expression of COX-2 and iNOS proteins in the lipopolysaccharide (LPS)-stimulated mouse leukaemic monocyte macrophage cell line (RAW 264.7) was further evaluated, due to the sufficient quantity of **9** (104.3 mg) isolated from this investigation. The results showed that **9** could effectively inhibit the LPS-induced expression of iNOS protein at 50 and 100 μM. Compound **9** also could significantly inhibit the expression of COX-2 at 25–100 μM (Figure 8).

Comparing with compound **9**, some other nonselective COX-2 inhibitors, such as ibuprofen (half maximal inhibitory concentration IC_50_ for COX-2: 30 μM) [44] and aspirin (IC_50_ for COX-2: 1.36 mM; for iNOS: 3 mM) [45,46], appear to be less effective in inhibiting COX-2 than **9**, whereas the selective COX-2 inhibitor celecoxib (IC50 for COX-2: 0.072 μM) [44] is more effective than **9**. Also, the inhibiting activity for iNOS expression of **9** was found to be 25–100 μM, showing that **9** has higher iNOS-inhibitory activity than aspirin (IC_50_: 3 mM).

## 3. Experimental Section

### 3.1. General Experimental Procedures 

Optical rotations were measured on a JASCO P-1020 polarimeter (JASCO Corporation, Tokyo, Japan). Ultraviolet (UV) spectra were recorded on a JASCO V-650 spectrophotometer (JASCO Corporation). Infrared (IR) spectra were recorded on JASCO FT/IR-4100 Fourier transform infrared spectrophotometer (JASCO Corporation, Tokyo, Japan). NMR spectra were recorded on a Varian Unity INOVA500 FT-NMR (Varian Inc., Palo Alto, CA, USA) instrument at 500 MHz for ^1^H and 125 MHz for ^13^C in CDCl_3_, and the chemical shifts were referenced to residual signals of TMS (*δ*_H_ 0.00 ppm) and the CDCl_3_ (*δ*_C_ 77.0 ppm). EIMS and ESIMS data were obtained by BRUKER APEX II mass spectrometer (Bruker, Bremen, Germany). Silica gel (230–400 mesh, Merck, Darmstadt, Germany) was used for column chromatography. Precoated silica gel plates (Merck, Kieselgel 60 F-254, 0.2 mm) were used for analytical thin-layer chromatography (TLC). High-performance liquid chromatography was performed on a Hitachi L-2455 high-performance liquid chromatography (HPLC) apparatus (Hitachi Ltd., Tokyo, Japan) with a Supelco C18 column (250 mm × 21.2 mm, 5 μm).

### 3.2. Animal Material 

The soft coral *Sarcophyton stellatum* Kukenthal (Alcyoniidea) was collected by hand via self-contained underwater breathing apparatus (SCUBA) at a depth of 10–15 m along the coast of Dongsha Atoll, Taiwan, and stored in at −20 °C until extraction. A voucher sample (NHSC 2009-04) was deposited at Department of Marine Biotechnology and Resources, National Sun Yat-sen University. The soft coral was identified by one of the authors (C.-F.D.). 

### 3.3. Extraction and Isolation 

The frozen bodies of *S. stellatum* (2.5 kg, wet wt.) were minced and extracted thoroughly with EtOAc. The combined extract was concentrated by reduced pressure and the solvent-free extract (39.2 g) was fractionated on a column of silica gel using pure *n*-hexane, EtOAc-*n*-hexane (1:100 to 10:1, gradient), and subsequently pure EtOAc as eluting solvents to yield 20 fractions (F1 to F20). Fractions F7 and F8 eluted with EtOAc-*n*-hexane (1:6 and 1:4, respectively) were combined and further purified over a silica gel column using EtOAc-*n*-hexane (1:8) and RP-18 gel column using MeOH–H_2_O (1:1) as eluents. The purified fraction was then chromatographed on RP-18 HPLC using MeOH–H_2_O (6:1 to 5:1, gradient) to afford **1** (1.0 mg), **6** (0.9 mg), and **7** (1.3 mg), eluted by MeOH–H_2_O (6:1); and **2** (3.2 mg) and **3** (3.7 mg), eluted by MeOH–H_2_O (5:1), subsequently. F10 and F11 eluted with EtOAc-*n*-hexane (1:1 and 2:1, respectively) were combined together and separated over a Si gel column using EtOAc–*n*-hexane (1:4), RP-18 gel column using MeOH–H_2_O (5:1), and on RP-18 HPLC using MeOH–H_2_O (5:1 to 4:1, gradient) to yield **5** (3.2 mg), **8** (0.8 mg), **9** (104.3 mg), **10** (4.7 mg), and **11** (5.8 mg), successively. F12 and F13 eluted with EtOAc-*n*-hexane (4:1 and 6:1, respectively) were combined and chromatographed on Si gel 60 column using EtOAc-*n*-hexane (1:2), RP-18 gel column using MeOH–H_2_O (2:1), and on RP-18 HPLC using MeOH–H_2_O (3:1 to 2:1, gradient) to yield **12** (2.6 mg), **13** (4.6 mg), and **14** (2.2 mg). Finally, F14 eluted with EtOAc-*n*-hexane (8:1) was isolated on Si gel 60 column using EtOAc-*n*-hexane (1:1), RP-18 gel column using MeOH–H_2_O (1:1), and on RP-18 HPLC using MeOH–H_2_O (2:1 to 4:1, gradient) to give **4** (3.1 mg), **15** (2.1 mg), and **16** (7.8 mg).

Stellatumolide A (**1**): colorless oil; [α]${}_{D}^{25}$ +57.2 (*c* 0.1, CHCl_3_); IR (neat) ν_max_ 3445, 2925, 2857, 1760, 1680, 1455, 1379, 1259, 1227, 1092, 1037 and 762 cm^–1^; UV (MeOH) λ_max_ 204 (log ε = 3.5); ^13^C (125 MHz, CDCl_3_) and ^1^H NMR (500 MHz, CDCl_3_) data, see Table 1 and Table 2, respectively; ^13^C (125 MHz, C_6_D_6_) δ 170.0 (C, C-16), 157.8 (C, C-1), 130.8 (C, C-12), 129.9 (CH, C-11), 129.5 (C, C-15), 114.0 (C, C-2), 92.1 (C, C-4), 83.8 (C, C-8), 72.2 (CH, C-3), 71.2 (CH, C-7), 41.2 (CH_2_, C-9), 40.1 (CH_2_, C-13), 39.1 (CH_2_, C-5), 32.1 (CH_2_, C-6), 24.7 (CH_2_, C-10), 22.6 (CH_2_, C-14), 23.2 (CH_3_, C-19), 20.8 (CH_3_, C-18), 16.5 (CH_3_, C-20), 8.7 (CH_3_, C-17); ^1^H (500 MHz, C_6_D_6_) δ 5.15 (1H, d, *J* = 11.0 Hz, H-11), 4.19 (1H, s, H-3), 3.93 (1H, d, *J* = 10.0 Hz, H-7), 2.29 (2H, m, H_2_-10), 2.12 (1H, dd, *J* = 13.0, 13.0 Hz, H-14), 2.01 (1H, ddd, *J* = 13.0, 8.0, 8.0 Hz, H-14),1.89 (1H, m, H-13), 1.80 (1H, m, H-9), 1.78 (1H, m, H-13), 1.73 (3H, s, H_3_-18), 1.68 (1H, m, H-9), 1.56 (3H, s, H_3_-20), 1.50 (1H, m, H-6), 1.49 (3H, s, H_3_-17), 1.30 (2H, m, H_2_-5), 1.22 (1H, m, H-6), 1.08 (3H, s, H_3_-19); ESIMS *m/z* 371 [M + Na]^+^, 353 [M − H_2_O + Na]^+^; HRESIMS *m/z* 371.1832 [M + Na]^+^ (calcd. for C_20_H_28_O_5_Na, 371.1834).

Stellatumolide B (**2**): colorless oil; [α]${}_{D}^{25}$ −59.4 (*c* 0.3, CHCl_3_); IR (neat) ν_max_ 3420, 2928, 2859, 1752, 1666, 1457, 1376, 1063, and 1020 cm^–1^; UV (MeOH) λ_max_ 275 (log ε = 4.3); ^13^C (125 MHz, CDCl_3_) and ^1^H NMR (500 MHz, CDCl_3_) data, see Table 1 and Table 2, respectively; HREIMS *m/z* 332.1985 (calcd. for C_20_H_28_O_4_, 332.1988).

Stellatumolide C (**3**): colorless oil; [α]${}_{D}^{25}$ −52.5 (*c* 0.3, CHCl_3_); IR (neat) ν_max_ 3419, 2965, 2927, 2856, 1749, 1666, 1457, 1377, 1063, and 1019 cm^–1^; UV (MeOH) λ_max_ 275 (log ε = 4.3); ^13^C (125 MHz, CDCl_3_) and ^1^H NMR (500 MHz, CDCl_3_) data, see Table 1 and Table 2, respectively; HREIMS *m/z* 332.1989 (calcd. for C_20_H_28_O_4_, 332.1988].

Stellatumonin A (**4**): colorless oil; [α]${}_{D}^{25}$ −220 (*c* 0.3, CHCl_3_); IR (neat) ν_max_ 3421, 2927, 2863, 1729, 1453, 1380, 1247, 1136, 1043 and 767 cm^–1^; ^13^C (125 MHz, CDCl_3_) and ^1^H NMR (500 MHz, CDCl_3_) data, see Table 1 and Table 2, respectively; HRESIMS *m/z* 341.2091, [M + Na]^+^ (calcd. For C_20_H_30_O_3_Na, 341.2093).

Stellatumonin B (**5**): colorless oil; [α]${}_{D}^{25}$ −105 (*c* 0.2, CHCl_3_); IR (neat) ν_max_ 2959, 2927, 2857, 1734, 1455, 1385, and 1049 cm^–1^; ^13^C (125 MHz, CDCl_3_) and ^1^H NMR (500 MHz, CDCl_3_) data, see Table 1 and Table 2, respectively. HRESIMS *m/z* 348.2301 (calcd. For C_21_H_32_O_4_, 348.2301).

Stellatumonone (**6**): colorless oil; [α]${}_{D}^{25}$ −63.5 (*c* 0.1, CHCl_3_); IR (neat) ν_max_ 2925, 2859, 1720, 1673, 1619, 1447, 1298, 1253 and 1093 cm^–1^; UV (MeOH) λ_max_ 203 (log ε = 4.4); ^13^C (125 MHz, CDCl_3_) and ^1^H NMR (500 MHz, CDCl_3_) data, see Table 1 and Table 2, respectively; HREIMS *m/z* 346.2143 (calcd. for C_21_H_30_O_4_, 346.2144].

### 3.4. Cytotoxicity Assay 

The cancer cell lines were purchased from the American Type Culture Collection (ATCC). Cytotoxicity assays of the isolated metabolites were performed using 3-(4,5-dimethylthiazol-2-yl)-2,5-diphenyltetrazolium bromide (MTT) colorimetric method [47,48]. Compound is considered inactive when IC_50_ > 20 μg/mL. The positive control used is doxorubicin. 

### 3.5. In Vitro Anti-Inflammatory Assay

RAW264.7 cells were purchased from the Food Industry Research and Development Institute (Hsinchu, Taiwan). Cell were cultured in DMEM, supplemented with RPMI-1640 medium supplemented with 10% heat-inactivated fetal bovine serum, 100 U/mL penicillin G, and 100 μg/mL streptomycin (Gibco/BRL, Gran Island, NY, USA), at 37 °C in an incubator with 5% CO_2_. RAW 264.7 cells were seeded onto a 6-well plate with 2 × 10^6^ cell per well and cultured for 24 h. The cells were pretreated with the compound **9** for 1 h and treated with lipopolysaccharide (LPS, 1 μg/mL) from *Escherichia coli* 055:B5 (Sigma-Aldrich, St Louis, MO, USA) in the presence or absence of the compound **9** (25, 50, and 100 µM). After 24 h LPS treatment, the cell lysates were prepared using Cell Lysis Buffer (Cell Signalling Technology, Beverly, MA, USA). The expression of COX-2 and iNOS proteins was measured using Western blotting analysis. 

### 3.6. Western Blotting Analysis

Protein concentrations were measured using a bicinchoninic acid (BCA) protein assay kit (Pierce, Rockford, IL, USA). Protein extracts were boiled, loaded into sodium dodecyl sulfate polyacrylamide gel electrophoresis (SDS-PAGE), and electrotransferred to polyvinylidene fluoride (PVDF) membranes. After blocking in 5% nonfat milk in TBST buffer (20 mM Tris-HCl, 120 mM NaCl, and 0.1% Tween 20) for 1 h, the membranes were incubated with antibodies for COX-2 (#4842), iNOS (#13120), and β-actin (#3700) (all purchased from Cell Signaling Technology, Danvers, MA, USA) at 4 °C with gentle agitation overnight. The membranes were then washed and incubated with horseradish peroxidase-labelled rabbit and mouse secondary antibodies (Jackson ImmunoResearch, West Grove, PA, USA) for 2 h at room temperature. After successive washes, the membranes were developed with an enhanced chemoluminescence (ECL) kit (Amersham Biosciences, Buckinghamshire, UK), and blots were visualized using a LAS3000 system (Fujifilm, Tokyo, Japan). Densitometric analysis was performed with ImageJ software (National Institute of Health, Bethesda, MD, USA).

### 3.7. Statistical Analysis

The data are expressed as the mean ± SD. One-way ANOVA followed by Tukey’s post-hoc test (Graphpad Prism 5.0, GraphPad Software, San Diego, CA, USA) was used to compare multiple groups according to the experiments. *p* values < 0.05 were considered statistically significant.

## 4. Conclusions

Six new polyoxygenated cembrane-based diterpenoids, **1**–**6**, together with ten known related compounds (**7**–**16**), were isolated from the Formosan soft coral *Sarcophyton stellatum*. Compound **1** possessed an unusual spiroketal unit between the oxetane (C-2/C-4) and the γ-lactone ring (C-2/C-16) in the cembranoid, suggesting the potential to discover more new molecular structures from marine organisms and warrant the further discovery of new medicines from the ocean. Compound **9** could effectively inhibit the accumulation of the proinflammatory COX-2 and iNOS proteins in LPS-stimulated RAW264.7 macrophage cells in a dose-response manner. Thus, compound **9** might be useful for discovery of an effective anti-inflammatory agent. 

## Figures and Tables

**Table 1:** ^13^C NMR data of compounds **1**–**6** and **18**.

Position	1 ^a^	2 ^a^	3 ^a^	4 ^a^	5 ^a^	6 ^a^	18 ^c,d^
1	157.9 (C) ^b^	152.2 (C)	152.2 (C)	147.8 (C)	63.3 (C)	155.3 (C)	151.0 (C)
2	113.4 (C)	147.8 (C)	148.2 (C)	76.2 (CH)	107.4 (C)	197.8 (C)	196.7 (C)
3	71.1 (CH)	117.4 (CH)	116.5 (CH)	125.3 (CH)	120.6 (CH)	125.3 (CH)	123.8 (CH)
4	92.2 (C)	73.7 (C)	74.2 (C)	136.1 (C)	142.8 (C)	153.2 (C)	155.8 (C)
5	38.3 (CH_2_)	40.4 (CH_2_)	38.8 (CH_2_)	36.2 (CH_2_)	37.6 (CH_2_)	29.2 (CH_2_)	37.6 (CH_2_)
6	31.5 (CH_2_)	26.7 (CH_2_)	26.9 (CH_2_)	25.9 (CH_2_)	25.7 (CH_2_)	25.9 (CH_2_)	24.9 (CH_2_)
7	71.3(CH)	77.3 (CH)	74.6 (CH)	61.9 (CH)	62.0 (CH)	62.3 (CH)	62.0 (CH)
8	83.5 (C)	74.6 (C)	74.9 (C)	59.7 (C)	60.7 (C)	60.6 (C)	60.5 (C)
9	40.4 (CH_2_)	37.8 (CH_2_)	38.0 (CH_2_)	39.2 (CH_2_)	37.1 (CH_2_)	37.6 (CH_2_)	37.2 (CH_2_)
10	24.0 (CH_2_)	21.6 (CH_2_)	22.1 (CH_2_)	24.1 (CH_2_)	22.5 (CH_2_)	22.9 (CH_2_)	22.7 (CH_2_)
11	129.2 (CH)	129.1 (CH)	128.5 (CH)	123.6 (CH)	124.6 (CH)	125.1 (CH)	125.9 (CH)
12	130.6 (C)	130.9 (C)	131.4 (C)	132.7 (C)	134.6 (C)	134.4 (C)	134.3 (C)
13	39.6 (CH_2_)	38.7 (CH_2_)	38.3 (CH_2_)	38.2 (CH_2_)	33.7 (CH_2_)	36.3 (CH_2_)	36.6 (CH_2_)
14	22.2 (CH_2_)	21.8 (CH_2_)	22.2 (CH_2_)	120.1 (CH)	21.6 (CH_2_)	28.8 (CH_2_)	29.3 (CH_2_)
15	128.8 (C)	123.3 (C)	123.7 (C)	77.4 (C)	71.3 (C)	125.0 (C)	127.8 (C)
16	171.1 (C)	170.3 (C)	170.3 (C)	78.8 (CH_2_)	68.8 (CH_2_)	168.5 (C)	168.8 (C)
17	8.6 (CH_3_)	8.7 (CH_3_)	8.8 (CH_3_)	22.1 (CH_3_)	11.7 (CH_3_)	14.1 (CH_3_)	15.1 (CH_3_)
18	20.1 (CH_3_)	29.7 (CH_3_)	29.5 (CH_3_)	17.1 (CH_3_)	16.8 (CH_3_)	24.2 (CH_3_)	19.6 (CH_3_)
19	22.5 (CH_3_)	26.1 (CH_3_)	25.6 (CH_3_)	16.3 (CH_3_)	17.8 (CH_3_)	17.1 (CH_3_)	17.4 (CH_3_)
20	16.3 (CH_3_)	15.6 (CH_3_)	15.9 (CH_3_)	16.6 (CH_3_)	16.3 (CH_3_)	16.1 (CH_3_)	15.5 (CH_3_)
OMe					49.3 (CH_3_)	52.0 (CH_3_)	

^a^ Spectra recorded in CDCl_3_ at 125 MHz at 25 °C. ^b^ Attached protons were determined by distortionless enhancement by polarization transfer (DEPT) experiments. The values are in ppm downfield from tetramethylsilane (TMS). ^c^ Spectra recorded in CDCl_3_ at 100 MHz at 25 °C. ^d^ The ^13^C data of the *n*-butoxy moiety were omitted.

**Table 2:** ^1^H NMR (CDCl_3_, 500 MHz) spectral data for compounds **1**–**6**.

Position	1 ^a^	2 ^a^	3 ^a^	4 ^a^	5 ^a^	6 ^a^
2				5.27 d (9.5)		
3	4.27 s	5.16 s	5.21 s	5.37 d (9.5)	5.21 s	6.10 s
5	1.72 m; 1.88 m	1.90 m; 1.97 m	1.79, m; 2.09, m	2.32 dd (14.0, 8.0); 2.09 m	2.28 2H, m	2.70 m; 2.97 m
6	1.94 2H, m	1.48 2H, m	1.49 m; 1.54 m	1.52 m; 1.83 m	1.70 m; 1.88 m	1.61 m; 2.00 m
7	4.27 br d (10.0) ^b^	3.44 dd (7.0, 5.0)	3.52 dd (9.5, 3.0)	2.85 dd (5.0, 5.0)	2.84 dd (7.5, 4.0)	2.65 dd (8.0, 3.0)
9	1.82 m; 1.92 m	1.52, m ; 1.63 m	1.49 m; 1.64 m	1.05 dd (14.0, 14.0, 5.0); 2.07 m	1.47, dd (13.0, 13.0); 1.98 m	1.30 m; 2.02 m
10	2.00, m (11.0); 2.44, m	1.82, m; 2.26 m	1.87, m; 2.17 ddd (14.0, 7.0, 7.0)	2.09 m; 2.11 m	1.94 m; 2.12 m	2.00 m; 2.18 m
11	5.26 d (11.0)	4.97 dd (6.5, 6.5)	5.00 dd (7.0, 7.0)	4.88 dd (7.0, 7.0)	5.24, m	5.05 dd (6.5, 6.5)
13	2.07 dd (13.0, 13.0); 2.40 m	2.22 m; 2.30 m	2.25 m; 2.30 m	2.58 2H, m	2.13 2H, m	2.16 2H, m
14	2.33 dd (13.0, 13.0); 2.53 dd (13.0, 7.5)	2.56 2H, m	2.57 2H, dd (7.5, 7.5)	5.68 ddd (8.5, 6.5, 2.0)	1.82 m; 2.14 m	2.40 dd (14.0, 7.0); 2.45 dd (14, 7.0)
16				3.57 d (9.0); 3.94 d (9.0)	3.64 d (10.0); 3.88 d (10.0)	
17	1.87 s	1.94 s	1.95 s	1.41 s	1.40 s	1.92 s
18	1.69 s	1.48 s	1.54 s	1.82 s	1.86 s	1.92 s
19	1.19 s	1.18 s	1.23 s	1.25 s	1.28 s	1.23 s
20	1.87 s	1.63 s	1.63 s	1.63 s	1.59 s	1.58 s
OMe					3.20 s	3.68 s

^a^ Spectra recorded at 25 °C. ^b^ Coupling constants (*J* values) in Hz in parentheses.

## Supplementary Materials

^1^H, ^13^C, DEPT, HMQC, COSY, HMBC and NOESY spectra of new compounds **1**–**6** are available online at http://www.mdpi.com/1660-3397/16/6/210/s1. Figure S1: ^1^H NMR spectrum of **1** in CDCl_3_, Figure S2: ^13^C NMR spectrum of **1** in CDCl_3_, Figure S3: DEPT spectra of **1** in CDCl_3_, Figure S4: HMQC spectrum of **1** in CDCl_3_, Figure S5: COSY spectrum of **1** in CDCl_3_, Figure S6: HMBC spectrum of **1** in CDCl_3_, Figure S7: NOESY spectrum of **1** in CDCl_3_, Figure S8: ^1^H NMR spectrum of **1** in C_6_D_6_, Figure S9: ^13^C NMR spectrum of **1** in C_6_D_6_. Figure S10: DEPT spectrum of **1** in C_6_D_6_, Figure S11: COSY spectrum of **1** in C_6_D_6_, Figure S12: HMBC spectrum of **1** in C_6_D_6_. Figure S13: NOESY spectrum of **1** in C_6_D_6_, Figure S14: ^1^H NMR spectrum of **2** in CDCl_3_, Figure S15: ^13^C NMR spectrum of **2** in CDCl_3_, Figure S16: DEPT spectra of **2** in CDCl_3_, Figure S17: HMQC spectrum of **2** in CDCl_3_, Figure S18: COSY spectrum of **2** in CDCl_3_, Figure S19: HMBC spectrum of **2** in CDCl_3_. Figure S20: NOESY spectrum of **2** in CDCl_3_, Figure S21: ^1^H NMR spectrum of **3** in CDCl_3_, Figure S22: ^13^C NMR spectrum of **3** in CDCl_3_, Figure S23: DEPT spectra of **3** in CDCl_3_, Figure S24: HMQC spectrum of **3** in CDCl_3_, Figure S25: COSY spectrum of **3** in CDCl_3_, Figure S26: HMBC spectrum of **3** in CDCl_3_, Figure S27: NOESY spectrum of **3** in CDCl_3_, Figure S28: ^1^H NMR spectrum of **4** in CDCl_3_, Figure S29: ^13^C NMR spectrum of **4** in CDCl_3_, Figure S30: DEPT spectra of **4** in CDCl_3_**,** Figure S31: HMQC spectrum of **4** in CDCl_3_, Figure S32: COSY spectrum of **4** in CDCl_3_, Figure S33: HMBC spectrum of **4** in CDCl_3_, Figure S34: NOESY spectrum of **4** in CDCl_3_, Figure S35: ^1^H NMR spectrum of **5** in CDCl_3_, Figure S36: ^13^C NMR spectrum of **5** in CDCl_3_, Figure S37: DEPT spectra of **5** in CDCl_3_, Figure S38: HMQC spectrum of **5** in CDCl_3_, Figure S39: COSY spectrum of **5** in CDCl_3_, Figure S40: HMBC spectrum of **5** in CDCl_3_**,** Figure S41: NOESY spectrum of **5** in CDCl_3_, Figure S42: ^1^H NMR spectrum of **6** in CDCl_3_, Figure S43: ^13^C NMR spectrum of **6** in CDCl_3_, Figure S44: HMQC spectrum of **6** in CDCl_3_, Figure S45: COSY spectrum of **6** in CDCl_3_, Figure S46: HMBC spectrum of **6** in CDCl_3_, Figure S47: NOESY spectrum of **6** in CDCl_3_.

## References

[1] Blunt J.W., Carroll A.R., Copp B.R., Davis R.A., Keyzers R.A., Prinsep M.R. (2018). Marine natural products. Nat. Prod. Rep..

[2] Bernstein J., Shmeuli U., Zadock E., Kashman Y., Néeman I. (1974). Sarcophine, a new epoxy cembranolide from marine origin original research article. Tetrahedron.

[3] Huang H.C., Ahmed A.F., Su J.H., Chao C.H., Wu Y.C., Chiang M.Y., Sheu J.H. (2006). Crassocolides A–F, cembranoids with a *trans*-fused lactone from the soft coral *Sarcophyton crassocaule*. J. Nat. Prod..

[4] Duh C.Y., Wang S.K., Chung S.G., Chou G.C., Dai C.F. (2000). Cytotoxic cembrenolides and steroids from the Formosan soft coral *Sarcophyton crassocaule*. J. Nat. Prod..

[5] Wang G.H., Huang H.C., Su J.H., Huang C.Y., Hsu C.H., Kuo Y.H., Sheu J.H. (2011). Crassocolides N–P, three cembranoids from the Formosan soft coral *Sarcophyton crassocaule*. Bioorg. Med. Chem. Lett..

[6] Elkhateeb A., El-Beih A.A., Gamal-Eldeen A.M., Alhammady M.A., Ohta S., Paré P.W., Hegazy M.E.F. (2014). New terpenes from the Egyptian soft coral *Sarcophyton ehrenbergi*. Mar. Drugs.

[7] Xi Z., Bie W., Chen W., Liu D., van Ofwegen L., Proksch P., Lin W. (2013). Sarcophyolides B–E, new cembranoids from the soft coral *Sarcophyton elegans*. Mar. Drugs.

[8] Hegazy M.E.F., Eldeen A.M.G., Shahat A.A., Abdel-Latif F.F., Mohamed T.A., Whittlesey B.R., Paré P.W. (2012). Bioactive hydroperoxyl cembranoids from the red sea soft coral *Sarcophyton glaucum*. Mar. Drugs.

[9] Li W., Zou Y.H., Ge M.X., Lou L.L., Xu Y.S., Ahmed A., Chen Y.Y., Zhang J.S., Tang G.H., Yin S. (2017). Biscembranoids and cembranoids from the soft coral *Sarcophyton elegans*. Mar. Drugs.

[10] Sun P., Yu Q., Li J., Riccio R., Lauro G., Bifulco G., Kurtan T., Mandi A., Tang H., Li T.J. (2016). Bissubvilides A and B, cembrane-capnosane heterodimers from the soft coral *Sarcophyton subviride*. J. Nat. Prod..

[11] Huang C.Y., Sung P.J., Uvarani C., Su J.H., Lu M.C., Hwang T.L., Dai C.F., Wu S.L., Sheu J.H. (2015). Glaucumolides A and B, biscembranoids with new structural type from a cultured soft coral *Sarcophyton glaucum*. Sci. Rep..

[12] Shaaban M., Issa M.Y., Ghani M.A., Hamed A., Abdelwahab A.B. (2018). New pyranosyl cembranoid diterpenes from *Sarcophyton trocheliophorum*. Nat. Prod. Res..

[13] Liang L.F., Chen W.T., Li X.W., Wang H.Y., Guo Y.W. (2017). New bicyclic cembranoids from the South China Sea soft coral *Sarcophyton trocheliophorum*. Sci. Rep..

[14] Ahmed A.F., Tsai C.R., Huang C.Y., Wang S.Y., Sheu J.H. (2017). Klyflaccicembranols A–I, new cembranoids from the soft coral *Klyxum flaccidum*. Mar. Drugs.

[15] Hegazy M.E.F., Elshamy A.I., Mohamed T.A., Hamed A.R., Ibrahim M.A.A., Ohta S., Paré P.W. (2017). Cembrene diterpenoids with ether linkages from *Sarcophyton ehrenbergi*: An anti-proliferation and molecular-docking assessment. Mar. Drugs.

[16] Lai K.H., You W.J., Lin C.C., El-Shazly M., Liao Z.J., Su J.H. (2017). Anti-inflammatory cembranoids from the soft coral *Lobophytum crassum*. Mar. Drugs.

[17] Huang C.Y., Tseng Y.J., Chokkalingam U., Hwang T.L., Hsu C.H., Dai C.F., Sung P.J., Sheu J.H. (2016). Bioactive isoprenoid-derived natural products from a Dongsha Atoll soft coral *Sinularia erecta*. J. Nat. Prod..

[18] Zhao M., Cheng S.M., Yuan W.P., Xi Y.Y., Li X.B., Dong J.Y., Huang K.X., Gustafson K.R., Yan P.C. (2016). Cembranoids from a Chinese collection of the soft coral *Lobophytum crassum*. Mar. Drugs.

[19] Abdel-Lateff A., Alarif W.M., Ayyad S.E.N., Al-Lihaibi S.S., Basaif S.A. (2015). New cytotoxic isoprenoid derivatives from the Red Sea soft coral *Sarcophyton glaucum*. Nat. Prod. Res..

[20] Lin K.H., Tseng Y.J., Chen B.W., Hwang T.L., Chen H.Y., Dai C.F., Sheu J.H. (2014). Tortuosenes A and B, new diterpenoid metabolites from the Formosan soft coral *Sarcophyton tortuosum*. Org. Lett..

[21] Chang Y.T., Wu C.Y., Tang J.Y., Huang C.Y., Liaw C.C., Wu S.H., Sheu J.H., Chang H.W. (2017). Sinularin induces oxidative stress-mediated G2/M arrest and apoptosis in oral cancer cells. Environ. Toxicol..

[22] Chang Y.T., Huang C.Y., Tang J.Y., Liaw C.C., Li R.N., Liu J.R., Sheu J.H., Chang H.W. (2017). Reactive oxygen species mediate soft corals-derived sinuleptolide-induced antiproliferation and DNA damage in oral cancer cells. Onco Targets Ther..

[23] Tseng S.P., Hung W.C., Huang C.Y., Lin Y.S., Chan M.Y., Lu P.L., Lin L., Sheu J.H. (2016). 5-Episinuleptolide decreases the expression of the extracellular matrix in early biofilm Formation of multi-drug resistant *Acinetobacter baumannii*. Mar. Drugs.

[24] Tsai T.C., Chen H.Y., Sheu J.H., Chiang M.Y., Wen Z.H., Dai C.F., Su J.H. (2015). Structural elucidation and structure—Anti-inflammatory activity relationships of cembranoids from cultured soft corals *Sinularia sandensis* and *Sinularia flexibilis*. J. Agric. Food Chem..

[25] Wu Y.J., Neoh C.A., Tsao C.Y., Su J.H., Li H.H. (2015). Sinulariolide suppresses human hepatocellular carcinoma cell migration and invasion by inhibiting matrix metalloproteinase-2/-9 through MAPKs and PI3K/Akt signaling pathways. Int. J. Mol. Sci..

[26] Lin Y.Y., Jean Y.H., Lee H.P., Chen W.F., Sun Y.M., Su J.H., Lu Y., Huang S.Y., Hung H.C., Sung P.J. (2013). A soft coral-derived compound, 11-*epi*-sinulariolide acetate suppresses inflammatory response and bone destruction in adjuvant-induced Arthritis. PLoS ONE.

[27] Rahelivao M.P., Lübken T., Gruner M., Kataeva O., Ralambondrahety R., Andriamanantoanina H., Checinski M.P., Bauer I., Knölker H.J. (2017). Isolation and structure elucidation of natural products of three soft corals and a sponge from the coast of Madagascar. Org. Biomol. Chem..

[28] Centko R.M., Ramon-Garcia S., Taylor T., Patrick B.O., Thompson C.J., Miao V.P., Andersen R.J. (2012). Ramariolides A−D, antimycobacterial butenolides isolated from the mushroom *Ramaria cystidiophora*. J. Nat. Prod..

[29] (2017). CONFLEX 7, Conflex Corp., Japan. http://www.conflex.net/index.html.

[30] Góreck M. (2015). A configurational and conformational study of (−)-Oseltamivir using a multi-chiroptical approach. Org. Biomol. Chem..

[31] Cheng Z.B., Liao Q., Chen Y., Fan C.Q., Huang Z.Y., Xu X.J., Yin S. (2014). Four new cembranoids from the soft coral *Sarcophyton* sp.. Magn. Reson. Chem..

[32] Yao L.G., Liu H.L., Guo Y.W., Mollo E. (2009). New cembranoids from the Hainan soft coral *Sarcophyton glaucum*. Helv. Chim. Acta.

[33] Jia R., Kurtan T., Mandi A., Yan X.H., Zhang W., Guo Y.W. (2013). Biscembranoids formed from an α,β-unsaturated γ-lactone ring as a dienophile: Structure revision and establishment of their absolute configurations using theoretical calculations of electronic circular dichroism spectra. J. Org. Chem..

[34] Zhang M., Long K., Huang S., Shi K., Mak T.C.W. (1992). A novel diterpenolide from the soft coral *Sarcophyton*
*solidun*. J. Nat. Prod..

[35] Liang L.F., Kurtán T., Mándi A., Gao L.X., Li J., Zhang W., Guo Y.W. (2014). Sarsolenane and capnosane diterpenes from the Hainan soft coral *Sarcophyton trocheliophorum* Marenzeller as PTP1B Inhibitors. Eur. J. Org. Chem..

[36] Kobayashi M. (1991). Marine terpenes and terpenoids. Part 12. Autoxidation of dihydrofuranocembranoids. J. Chem. Res..

[37] Quang T.H., Ha T.T., Minh C.V., Kiem P.V., Huong H.T., Ngan N.T., Nhiem N.X., Tung N.H., Tai B.H., Thuy D.T. (2011). Cytotoxic and anti-inflammatory cembranoids from the Vietnamese soft coral *Lobophytum laevigatum*. Bioorg. Med. Chem..

[38] Lin S.T., Wang S.K., Duh C.Y. (2011). Cembranoids from the Dongsha Atoll soft coral *Lobophytum crassum*. Mar. Drugs.

[39] Kobayashi M., Hirase T. (1990). Marine terpenes and terpenoids. XI.: Structures of new dihydrofuranocembranoids isolated from a *Sarcophyton* sp. soft coral of Okinawa. Chem. Pharm. Bull..

[40] Grote D., Shaker K.H., Soliman H.S.M., Hegazi M.M., Seifert K. (2008). Cembranoid diterpenes from the soft corals *Sarcophyton* sp. and *Sarcophyton glaucum*. Nat. Prod. Commun..

[41] Shaker K.H., Muller M., Ghani M.A., Dahse H.M., Seifert K. (2010). Terpenes from the soft corals *Litophyton arboreum* and *Sarcophyton ehrenbergi*. Chem. Biodivers..

[42] Czarkie D., Carmely S., Groweiss A., Kashman Y. (1985). Attempted acid-catalyzed transannular reactions in the cembranoids. Tetrahedron.

[43] Grote D., Soliman H.S., Shaker K.H., Hamza M., Seifert K. (2006). Cembranoid diterpenes and a briarane diterpene from corals. Nat. Prod. Res..

[44] Kim K.J., Choi M.J., Shin J.S., Kim M., Choi H.E., Kang S.M., Jin J.H., Lee K.T., Lee J.Y. (2014). Synthesis, biological evaluation, and docking analysis of a novel family of 1-methyl-1H-pyrrole-2,5-diones as highly potent and selective cyclooxygenase-2 (COX-2) inhibitors. Bioorg. Med. Chem. Lett..

[45] Yun Y., Chen P., Zheng C.L., Yang Y., Duan W.G., Wang L., He B., Ma J.Q., Wang D.H., Shen Z.Q. (2007). Copper-aspirin complex inhibits cyclooxygenase-2 more selectively than aspirin. Yakugaku Zasshi.

[46] Amin A.R., Vyas P., Attur M., Leszczynska-Piziak J., Patel I.R., Weissmann G., Abramson S.B. (1995). The mode of action of aspirin-like drugs: Effect on inducible nitric oxide synthase. Proc. Natl. Acad. Sci. USA.

[47] Alley M.C., Scudiero D.A., Monks A., Hursey M.L., Czerwinski M.J., Fine D.L., Abbott B.J., Mayo J.G., Shoemaker R.H., Boyd M.R. (1988). Feasibility of drug screening with panels of human tumor cell lines using a microculture tetrazolium assay. Cancer Res..

[48] Scudiero D.A., Shoemaker R.H., Paull K.D., Monks A., Tierney S., Nofziger T.H., Currens M.J., Seniff D., Boyd M.R. (1988). Evaluation of a soluble tetrazolium/formazan assay for cell growth and drug sensitivity in culture using human and other tumor cell lines. Cancer Res..

