# Supplementary material for: Isolation and Structure Elucidation of Cembranoids from a Dongsha Atoll Soft Coral Sarcophyton stellatum
Source: Mar Drugs. 2018 Jun 14;16(6):210. doi: 10.3390/md16060210 (PMC6025080; doi:10.3390/md16060210)
Supplement: Supplementary file 1 [file marinedrugs-16-00210-s001.pdf]

SUPPORTING INFORMATION

Isolation and Structure Elucidation of Cembranoids from a Dongsha Atoll Soft Coral *Sarcophyton stellatum*

Atallah F. Ahmed [1,2,†], Yi-Wei Chen [1,†], Chiung-Yao Huang [1,†], Yen-Ju Tseng [1], Chi-Chen Lin [3], Chang-Feng Dai [4], Yang-Chang Wu [5] and Jyh-Horng Sheu [1,5,6,7,*]

[1] Department of Marine Biotechnology and Resources, National Sun Yat-sen University, Kaohsiung 804, Taiwan; afahmed@KSU.EDU.SA (A.F.A.); m985020005@student.nsysu.edu.tw (Y.-W.C.); huangcy@mail.nsysu.edu.tw (C.-Y.H.); d935020003@student.nsysu.edu.tw (Y.-J.T.)

[2] Department of Pharmacognosy, College of Pharmacy, King Saud University, Riyadh 11451, Saudi Arabia

[3] Institute of Biomedical Sciences, National Chung Hsing University, Taichung 402, Taiwan; lincc@dragon.nchu.edu.tw

[4] Institute of Oceanography, National Taiwan University, Taipei 112, Taiwan; corallab@ntu.edu.tw

[5] Graduate Institute of Natural Products, Kaohsiung Medical University, Kaohsiung 807, Taiwan; yachwu@kmu.edu.tw

[6] Frontier Center for Ocean Science and Technology, National Sun Yat-sen University, Kaohsiung 804, Taiwan

[7] Department of Medical Research, China Medical University Hospital, China Medical University, Taichung 404, Taiwan

* Correspondence: sheu@mail.nsysu.edu.tw; Tel.: +886-7-525-2000 (ext. 5030); Fax: +886-7-525-5020.

† *These authors contributed equally to this work.*

Corresponding author: E-mail: sheu@mail.nsysu.edu.tw; Fax: +886-7-525-5020; Tel: +886-7-525-2000 ext. 5030

List of Supplementary material:

No	Content	page	No	Content	page
Figure S1	^{1}H NMR spectrum of **1** in $CDCl_3$	3	Figure S25	COSY spectrum of **3** in $CDCl_3$	27
Figure S2	^{13}C NMR spectrum of **1** in $CDCl_3$	4	Figure S26	HMBC spectrum of **3** in $CDCl_3$	28
Figure S3	DEPT spectra of **1** in $CDCl_3$	5	Figure S27	NOESY spectrum of **3** in $CDCl_3$	29
Figure S4	HMQC spectrum of **1** in $CDCl_3$	6	Figure S28	^{1}H NMR spectrum of **4** in $CDCl_3$	30
Figure S5	COSY spectrum of **1** in $CDCl_3$	7	Figure S29	^{13}C NMR spectrum of **4** in $CDCl_3$	31
Figure S6	HMBC spectrum of **1** in $CDCl_3$	8	Figure S30	DEPT spectra of **4** in $CDCl_3$	32
Figure S7	NOESY spectrum of **1** in $CDCl_3$	9	Figure S31	HMQC spectrum of **4** in $CDCl_3$	33
Figure S8	^{1}H NMR spectrum of **1** in C_6D_6	10	Figure S32	COSY spectrum of **4** in $CDCl_3$	34
Figure S9	^{13}C NMR spectrum of **1** in C_6D_6	11	Figure S33	HMBC spectrum of **4** in $CDCl_3$	35
Figure S10	DEPT spectrum of **1** in C_6D_6	12	Figure S34	NOESY spectrum of **4** in $CDCl_3$	36
Figure S11	COSY spectrum of **1** in C_6D_6	13	Figure S35	^{1}H NMR spectrum of **5** in $CDCl_3$	37
Figure S12	HMBC spectrum of **1** in C_6D_6	14	Figure S36	^{13}C NMR spectrum of **5** in $CDCl_3$	38
Figure S13	NOESY spectrum of **1** in C_6D_6	15	Figure S37	DEPT spectra of **5** in $CDCl_3$	39
Figure S14	^{1}H NMR spectrum of **2** in $CDCl_3$	16	Figure S38	HMQC spectrum of **5** in $CDCl_3$	40
Figure S15	^{13}C NMR spectrum of **2** in $CDCl_3$	17	Figure S39	COSY spectrum of **5** in $CDCl_3$	41
Figure S16	DEPT spectra of **2** in $CDCl_3$	18	Figure S40	HMBC spectrum of **5** in $CDCl_3$	42
Figure S17	HMQC spectrum of **2** in $CDCl_3$	19	Figure S41	NOESY spectrum of **5** in $CDCl_3$	43
Figure S18	COSY spectrum of **2** in $CDCl_3$	20	Figure S42	^{1}H NMR spectrum of **6** in $CDCl_3$	44
Figure S19	HMBC spectrum of **2** in $CDCl_3$	21	Figure S43	^{13}C NMR spectrum of **6** in $CDCl_3$	45
Figure S20	NOESY spectrum of **2** in $CDCl_3$	22	Figure S44	HMQC spectrum of **6** in $CDCl_3$	46
Figure S21	^{1}H NMR spectrum of **3** in $CDCl_3$	23	Figure S45	COSY spectrum of **6** in $CDCl_3$	47
Figure S22	^{13}C NMR spectrum of **3** in $CDCl_3$	24	Figure S46	HMBC spectrum of **6** in $CDCl_3$	48
Figure S23	DEPT spectra of **3** in $CDCl_3$	25	Figure S47	NOESY spectrum of **6** in $CDCl_3$	49
Figure S24	HMQC spectrum of **3** in $CDCl_3$	26			

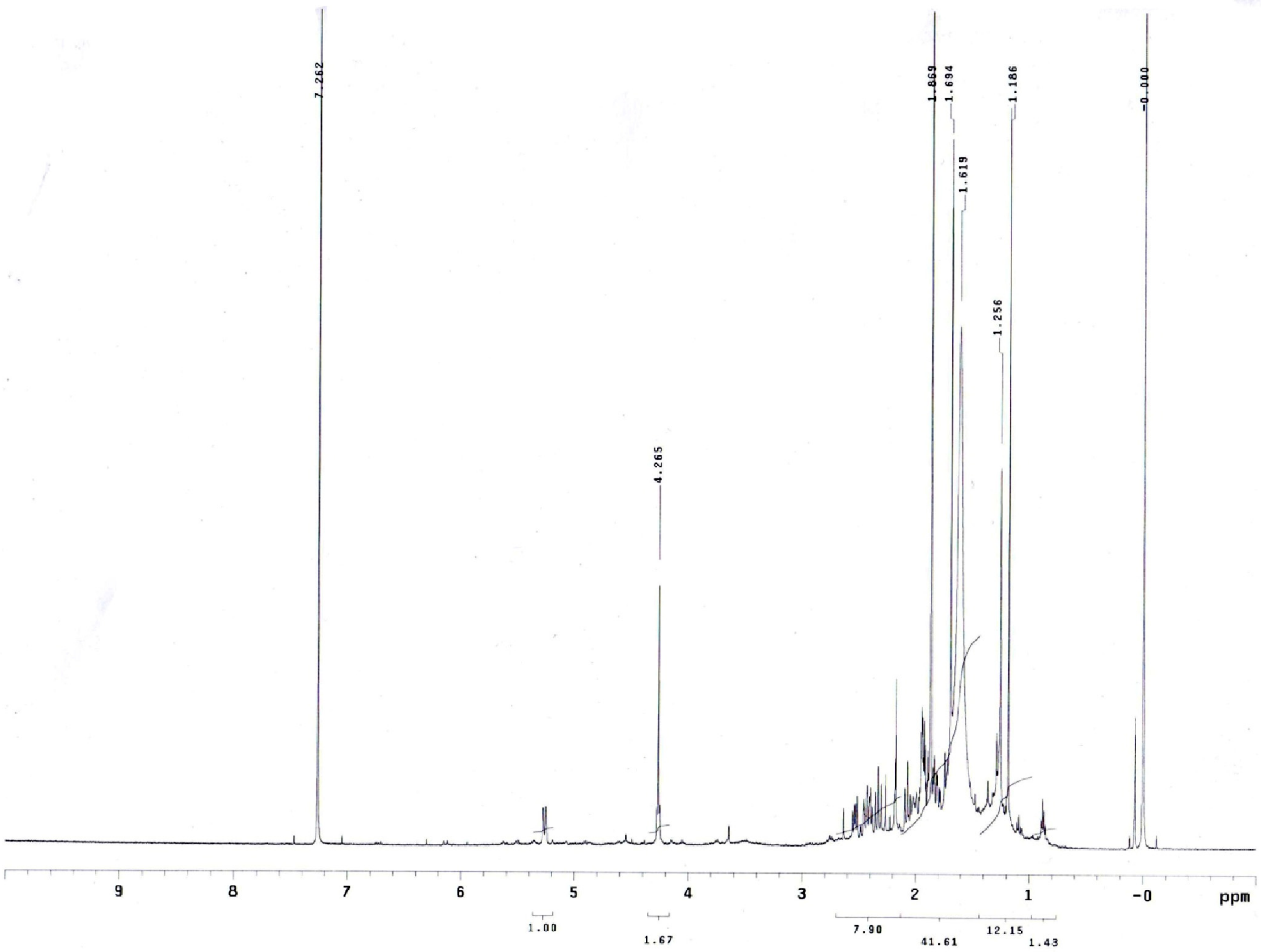

Figure S1. ^{1}H NMR spectrum of **1** in $CDCl_3$

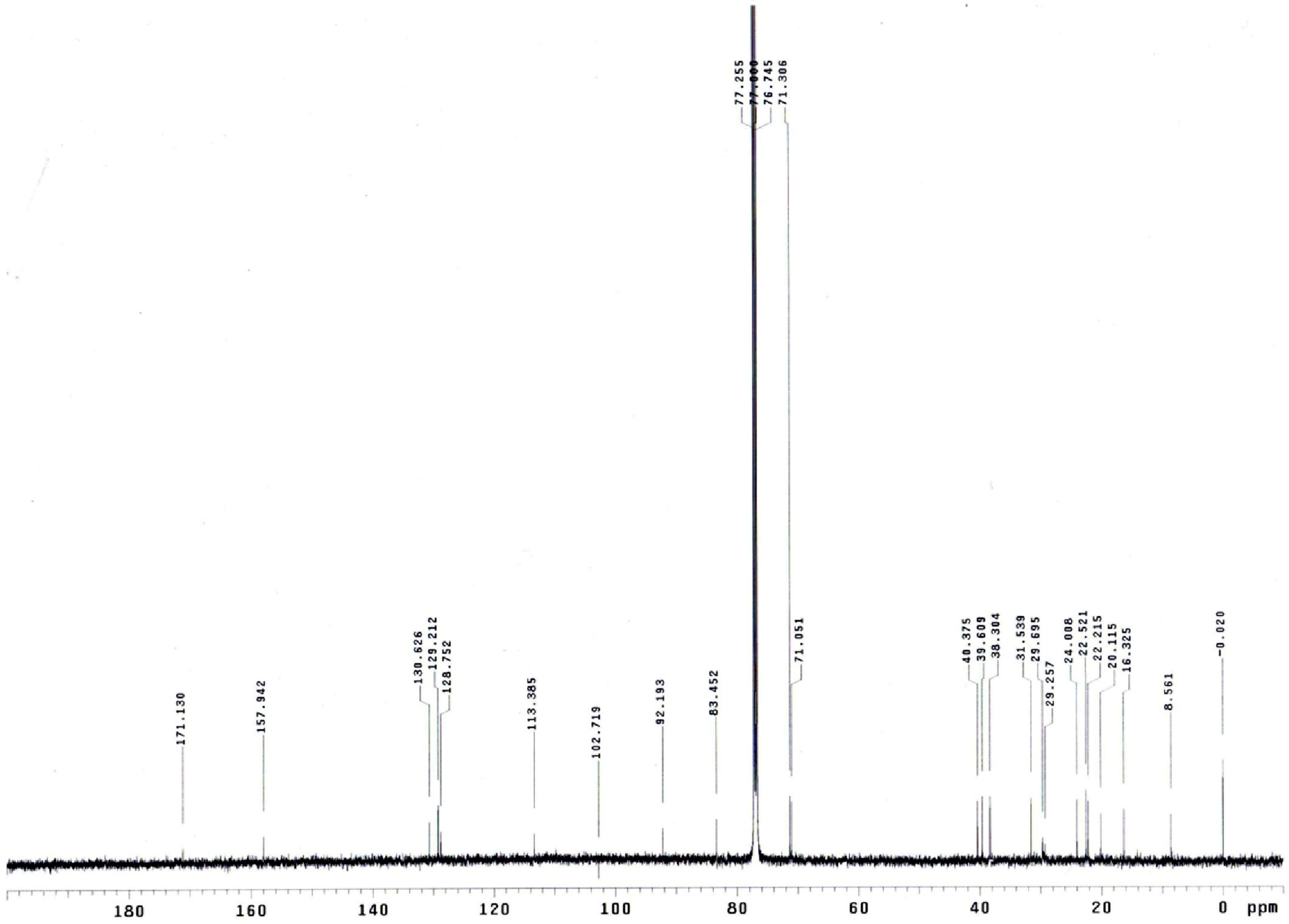

Figure S2. ^{13}C NMR spectrum of **1** in $CDCl_3$

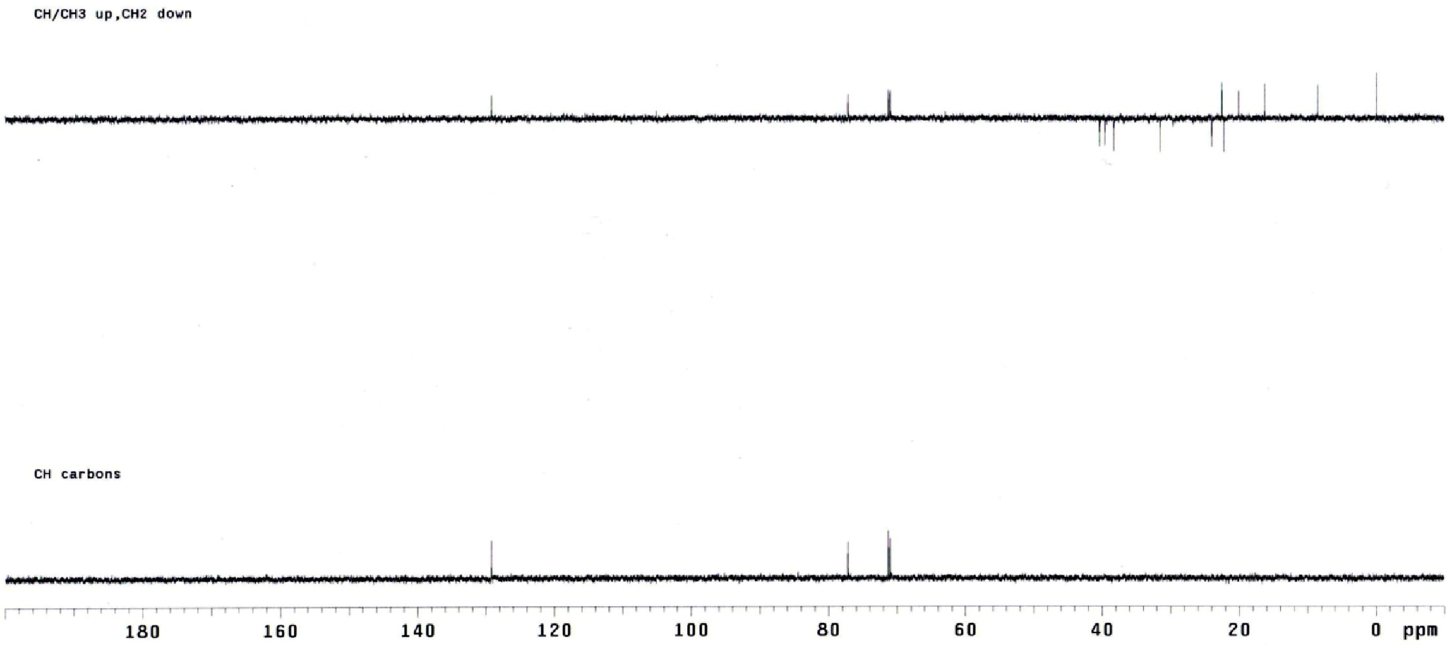

Figure S3. DEPT spectra of **1** in $CDCl_3$

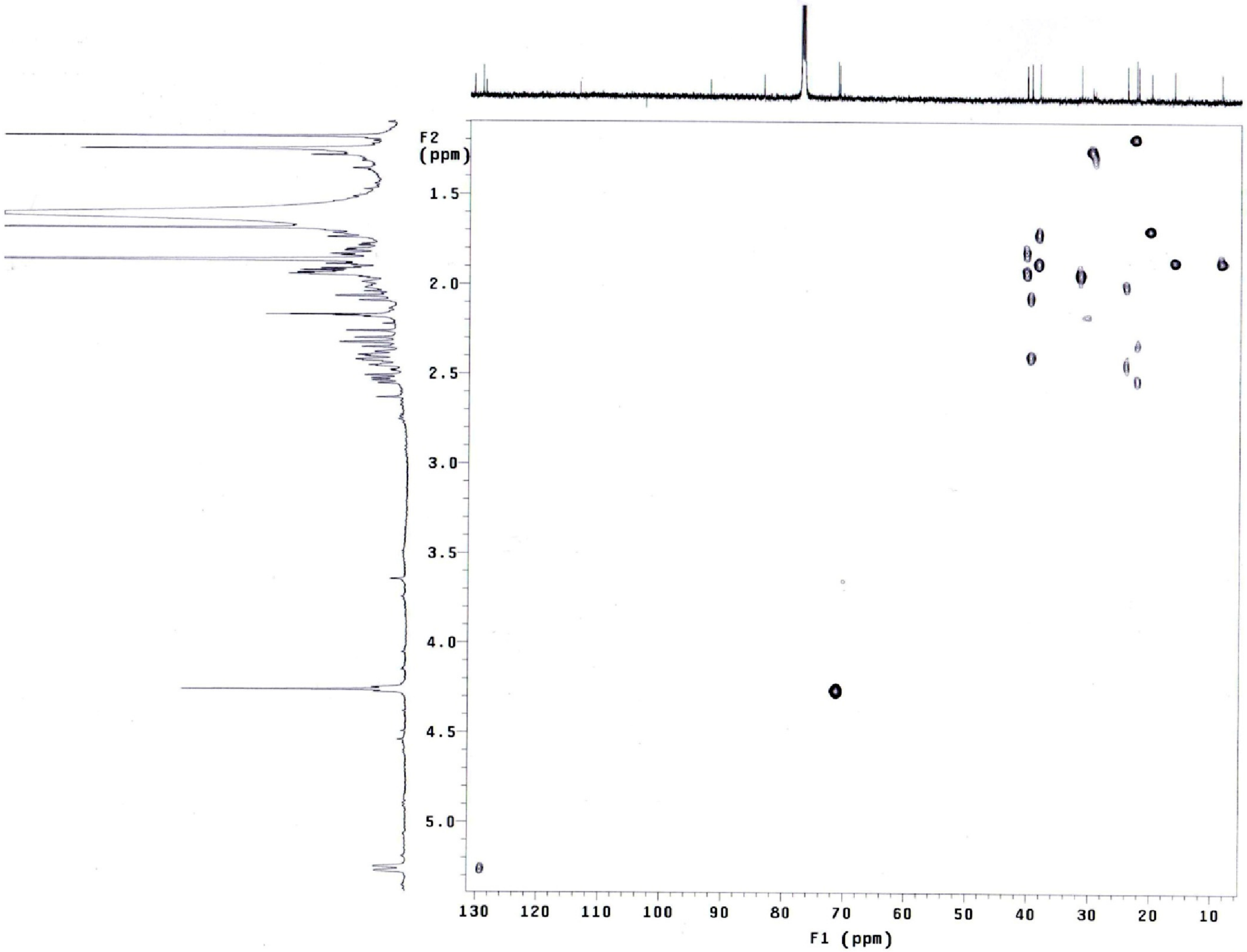

Figure S4. HMQC spectrum of **1** in $CDCl_3$

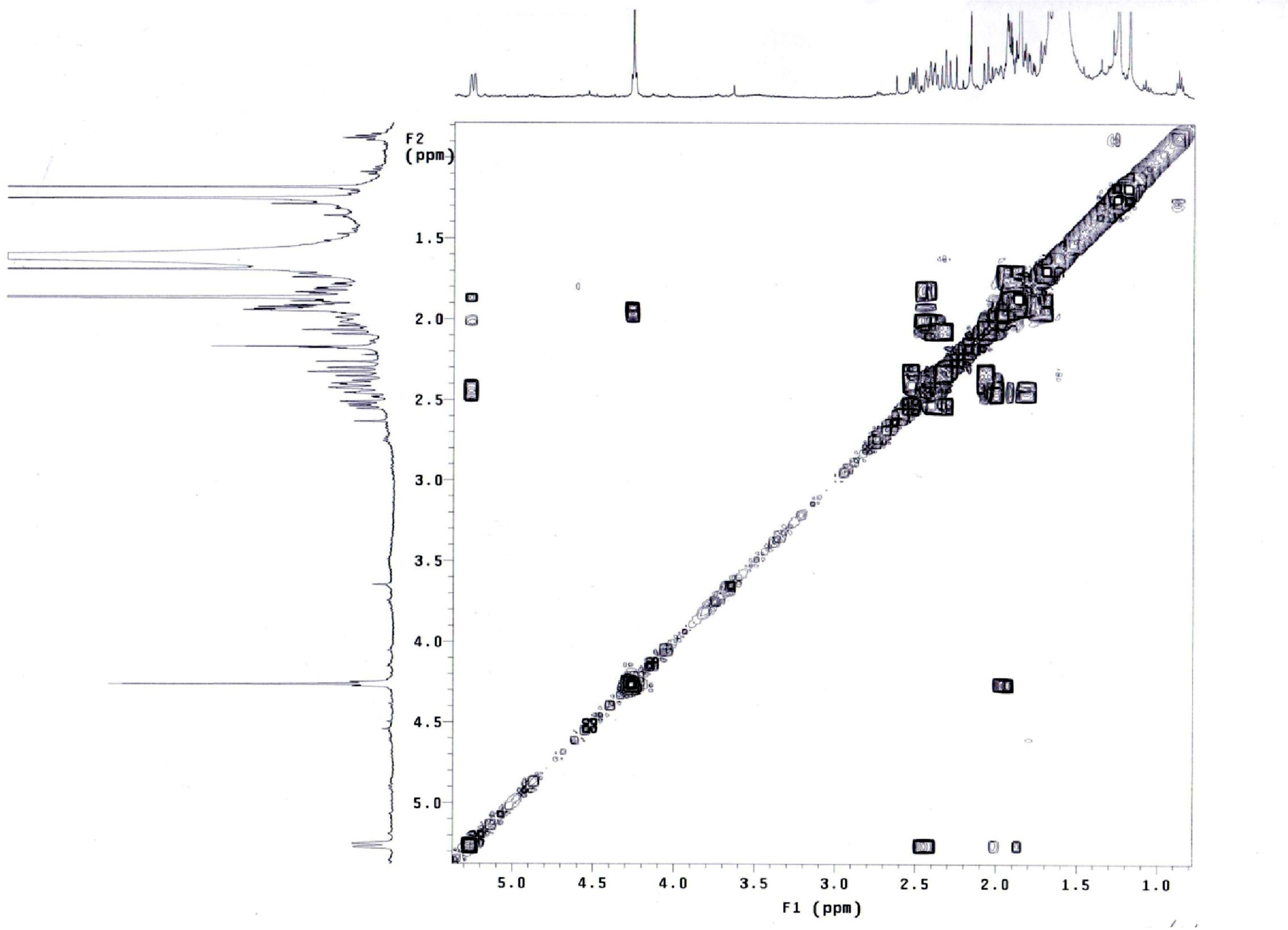

Figure S5. COSY spectrum of **1** in $CDCl_3$

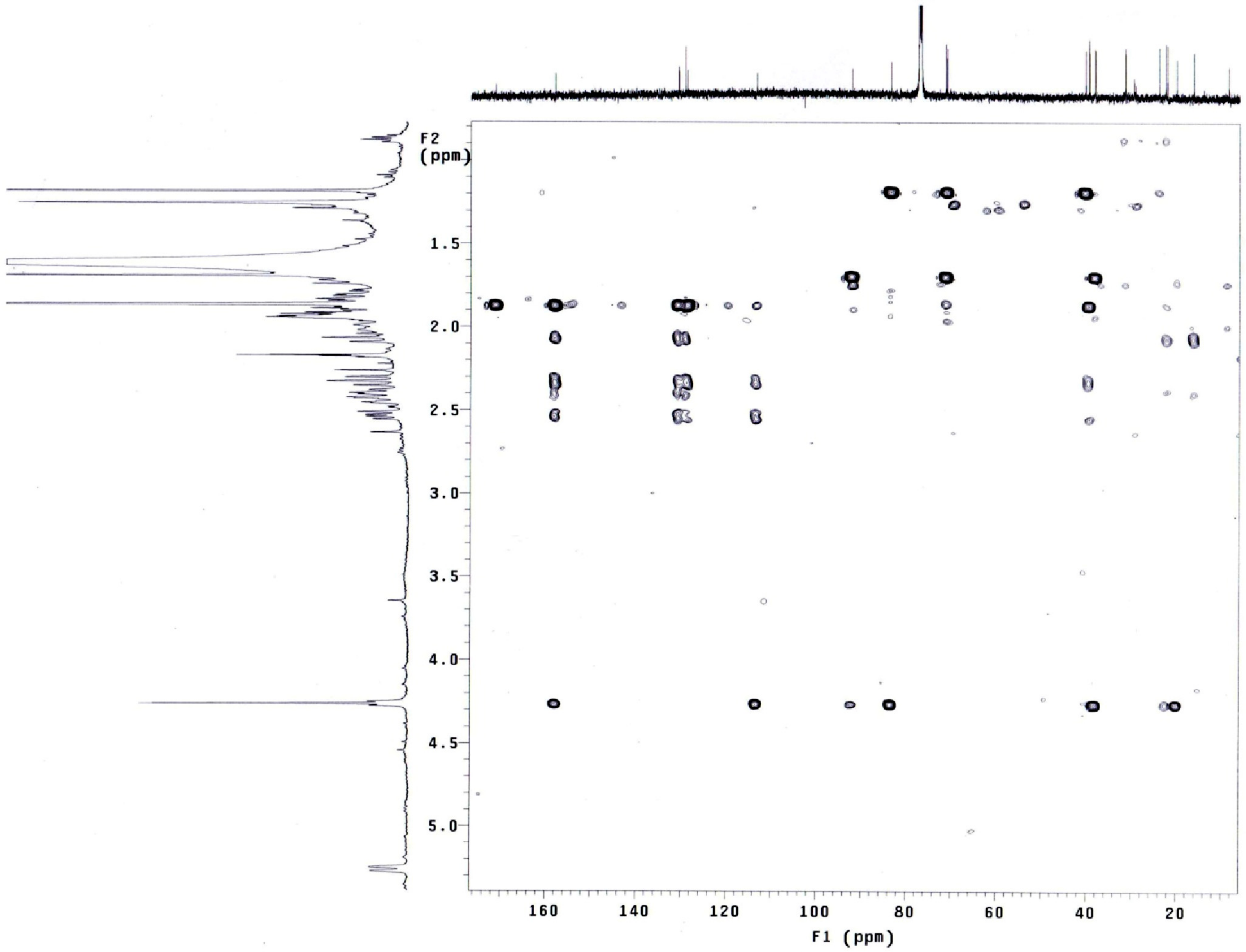

Figure S6. HMBC spectrum of **1** in $CDCl_3$

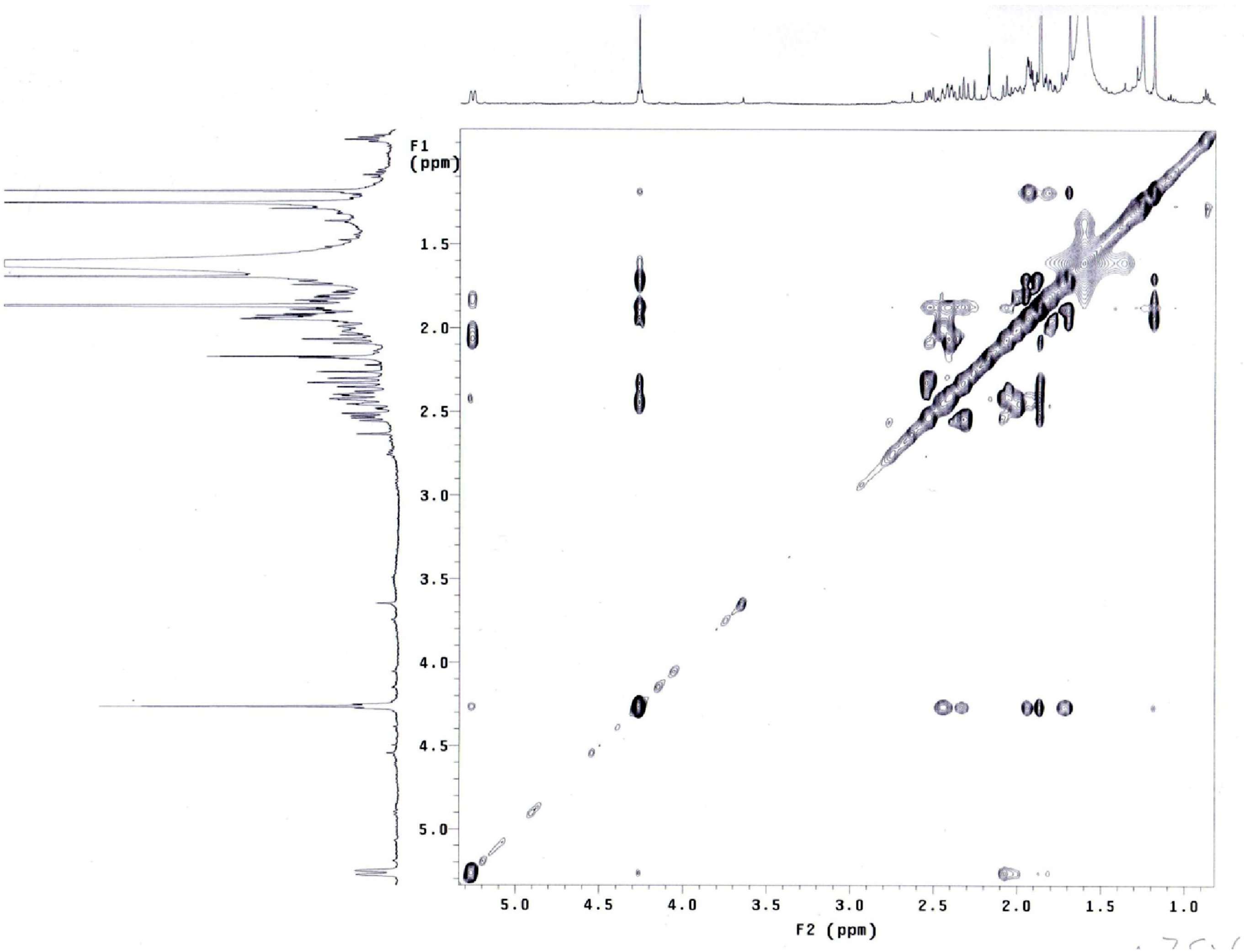

Figure S7. NOESY spectrum of **1** in $CDCl_3$

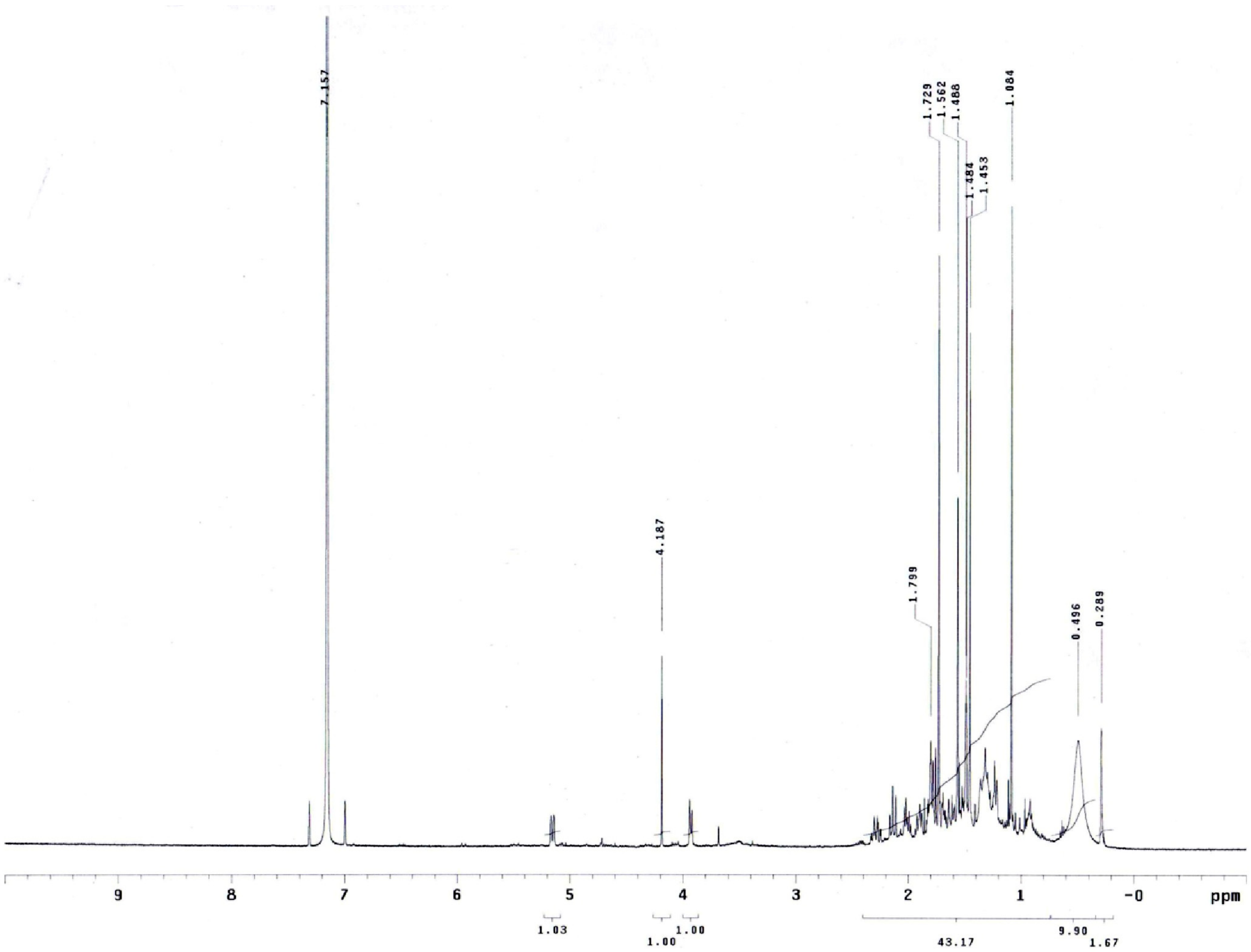

Figure S8. ^{1}H NMR spectrum of **1** in C_6D_6

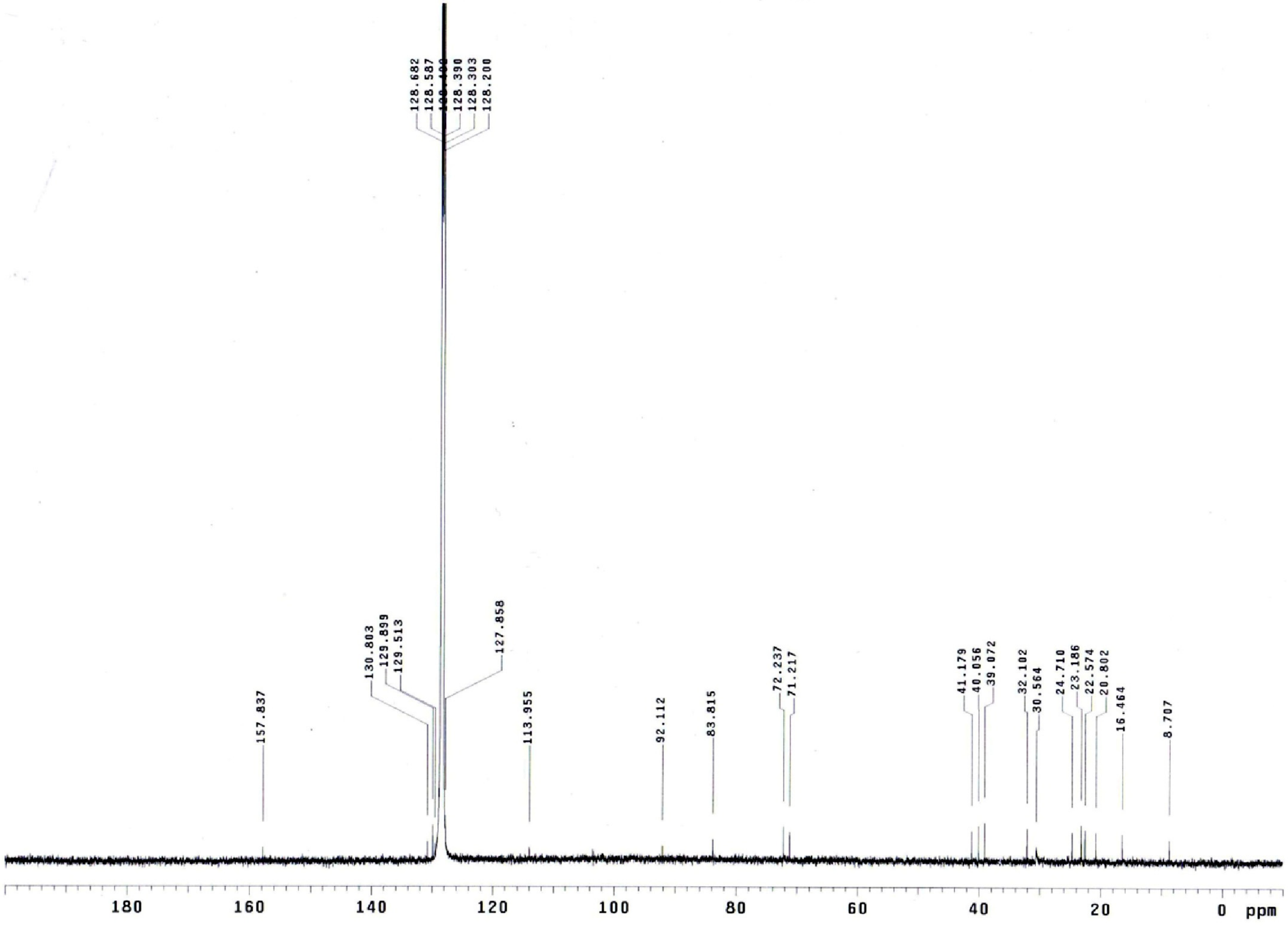

Figure S9. ^{13}C NMR spectrum of **1** in C_6D_6

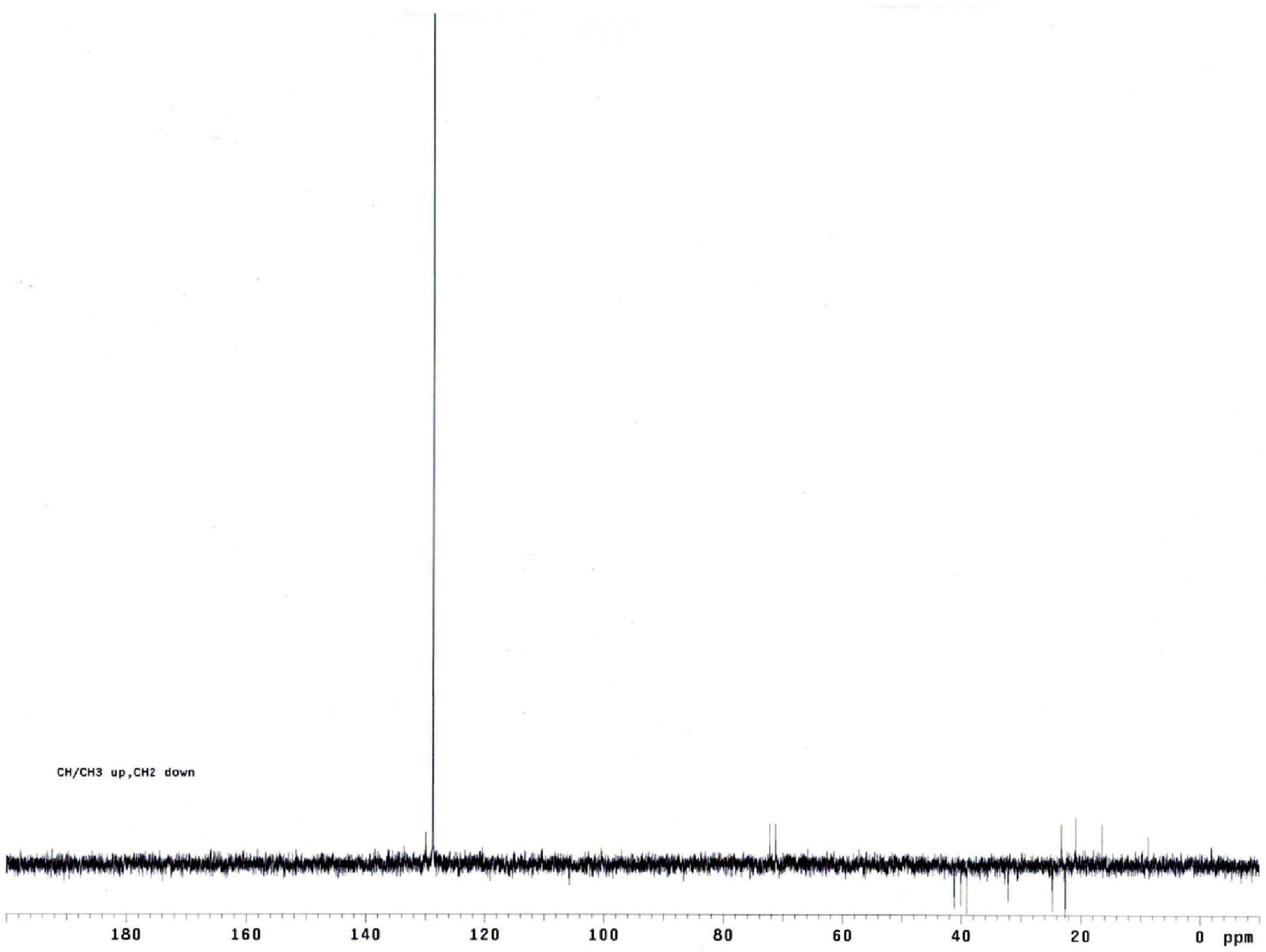

Figure S10. DEPT spectrum of **1** in C_6D_6

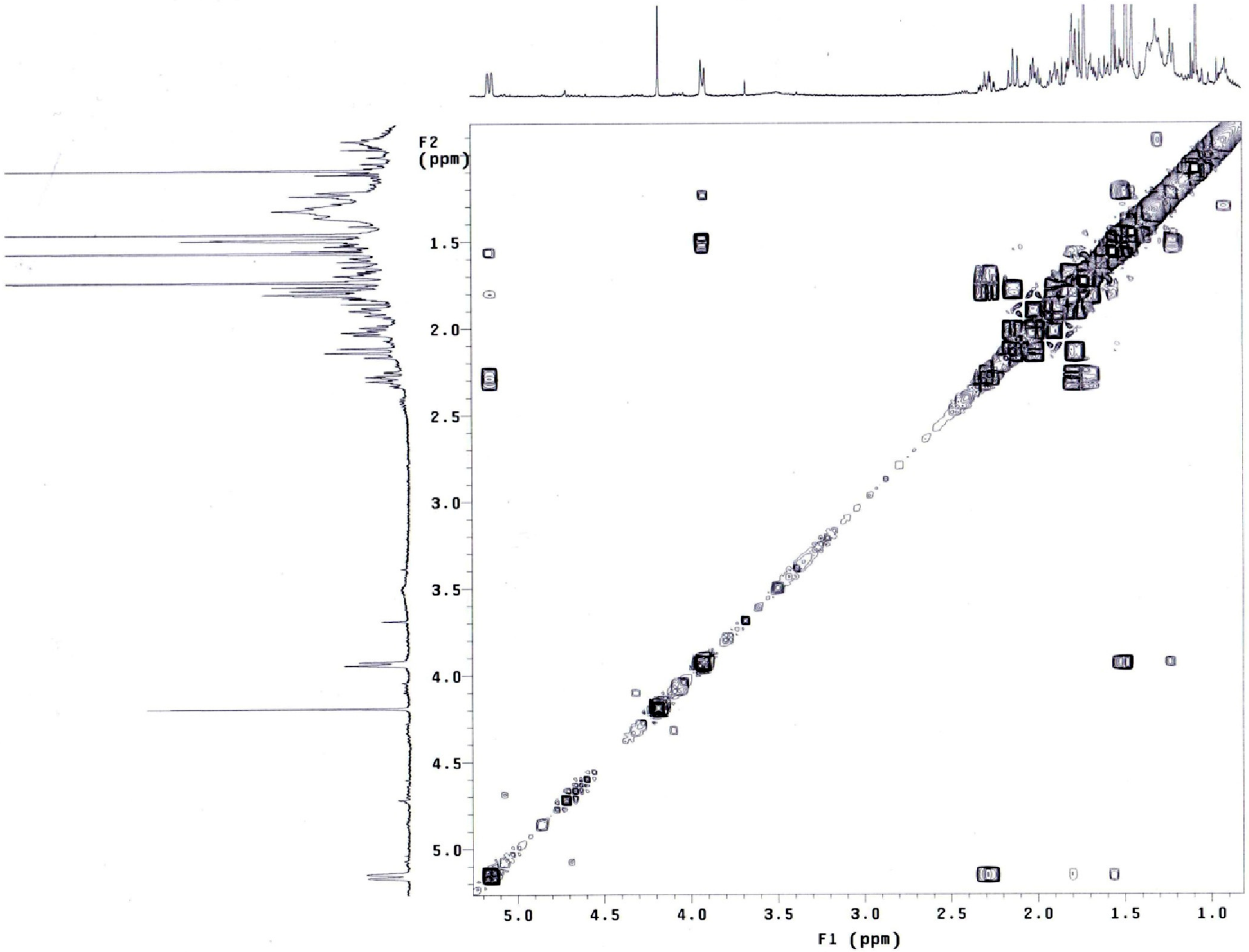

Figure S11. COSY spectrum of **1** in C_6D_6

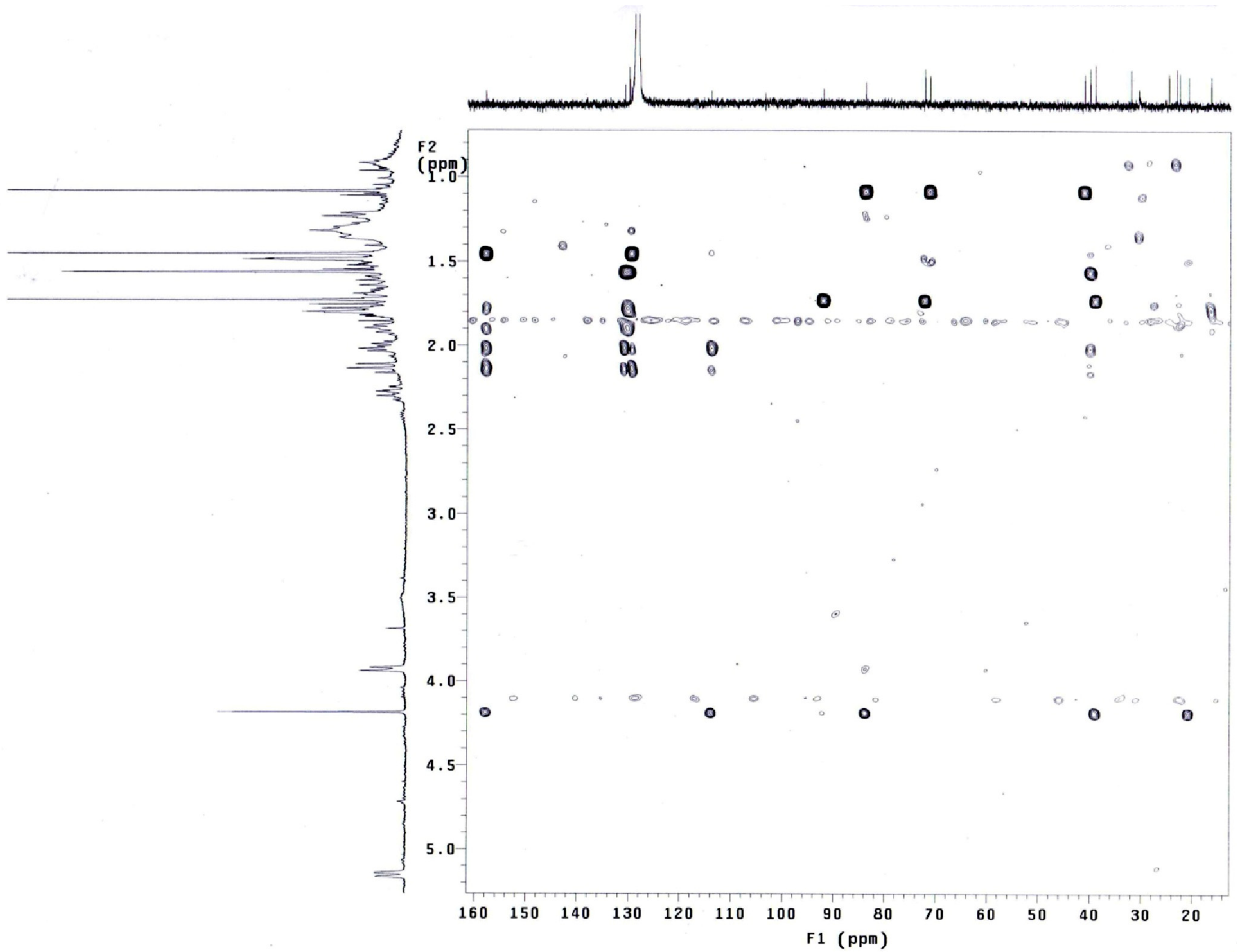

Figure S12. HMBC spectrum of **1** in C_6D_6

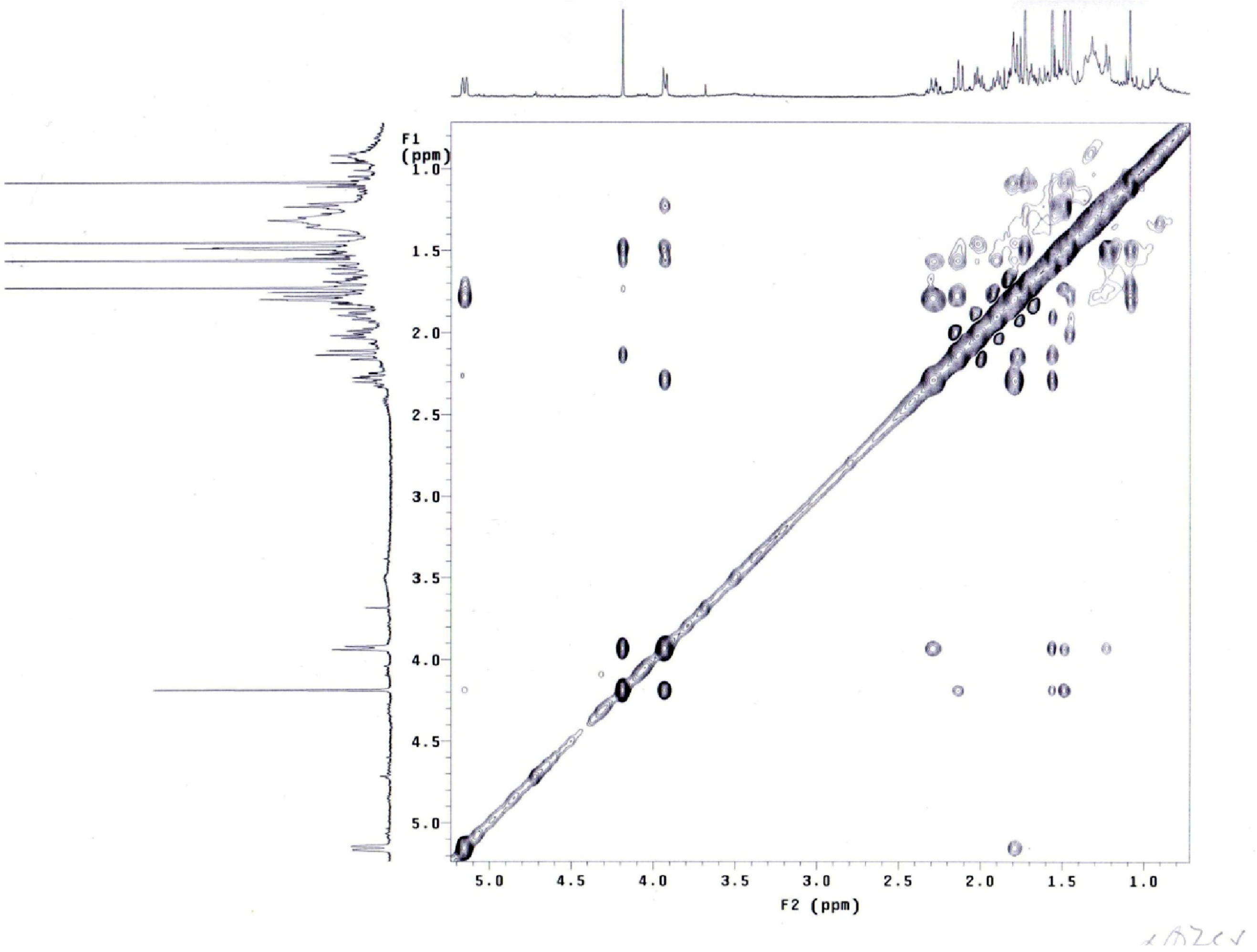

Figure S13. NOESY spectrum of **1** in C_6D_6

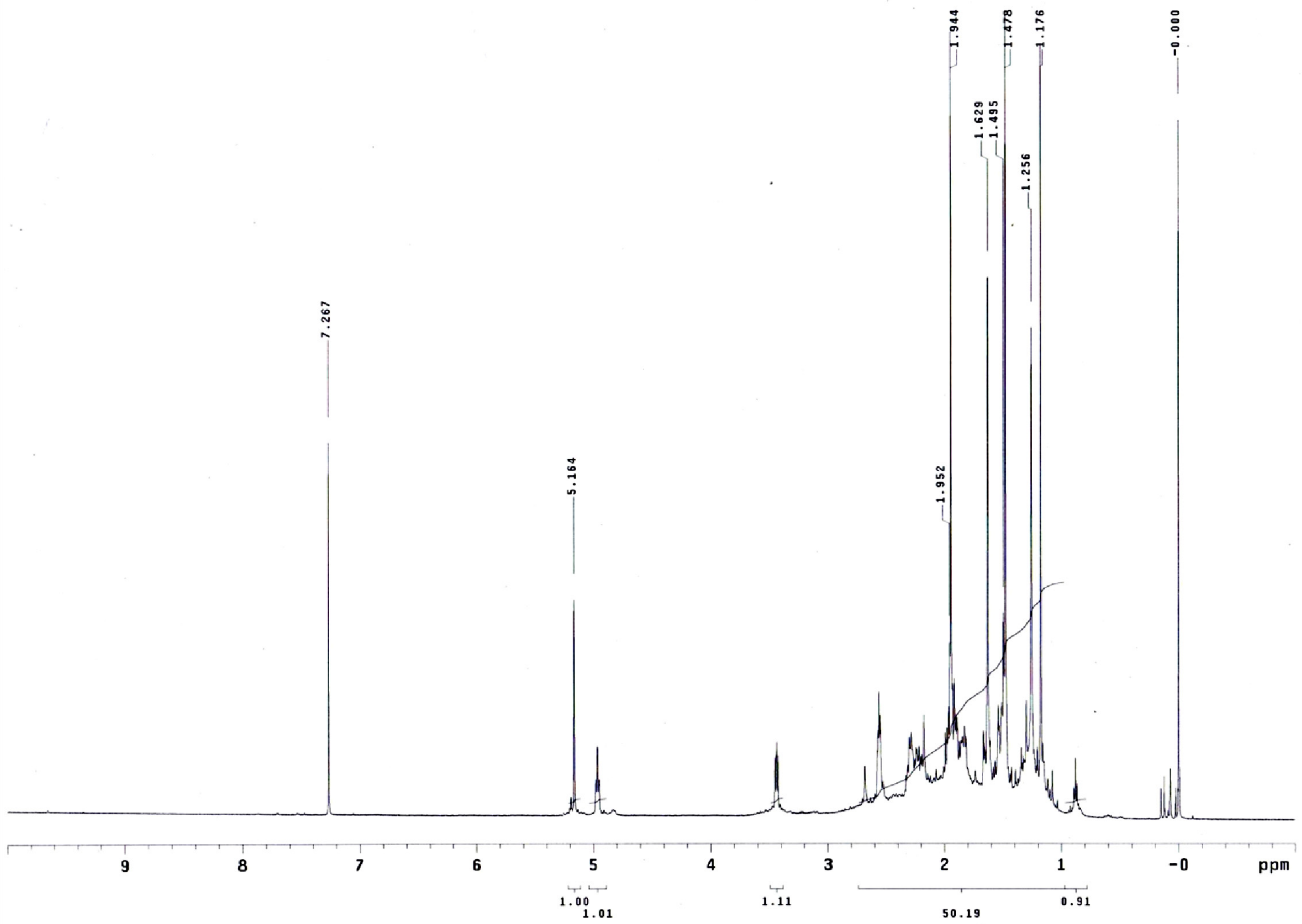

Figure S14. ^{1}H NMR spectrum of **2** in $CDCl_3$

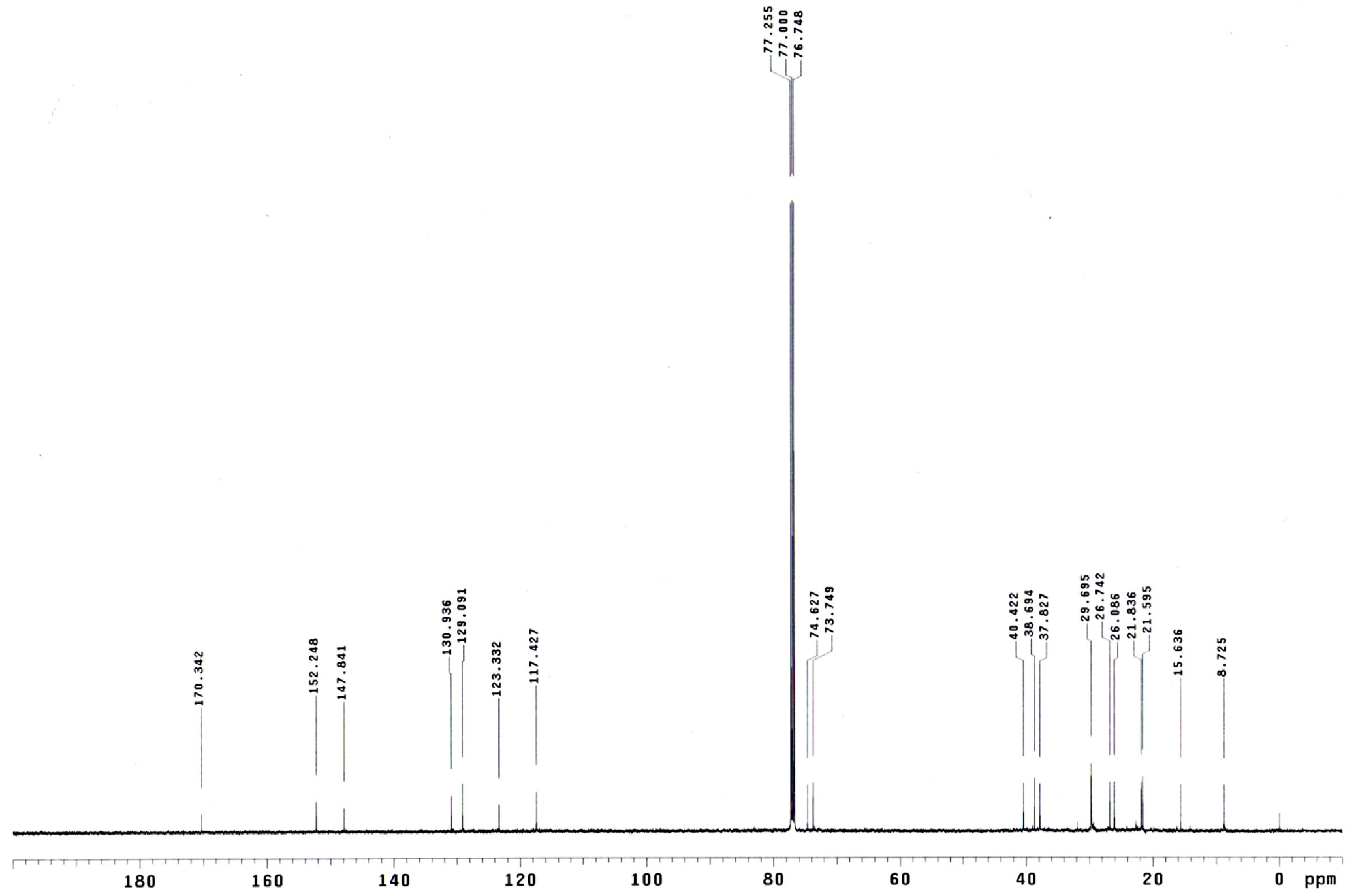

Figure S15. ^{13}C NMR spectrum of **2** in $CDCl_3$

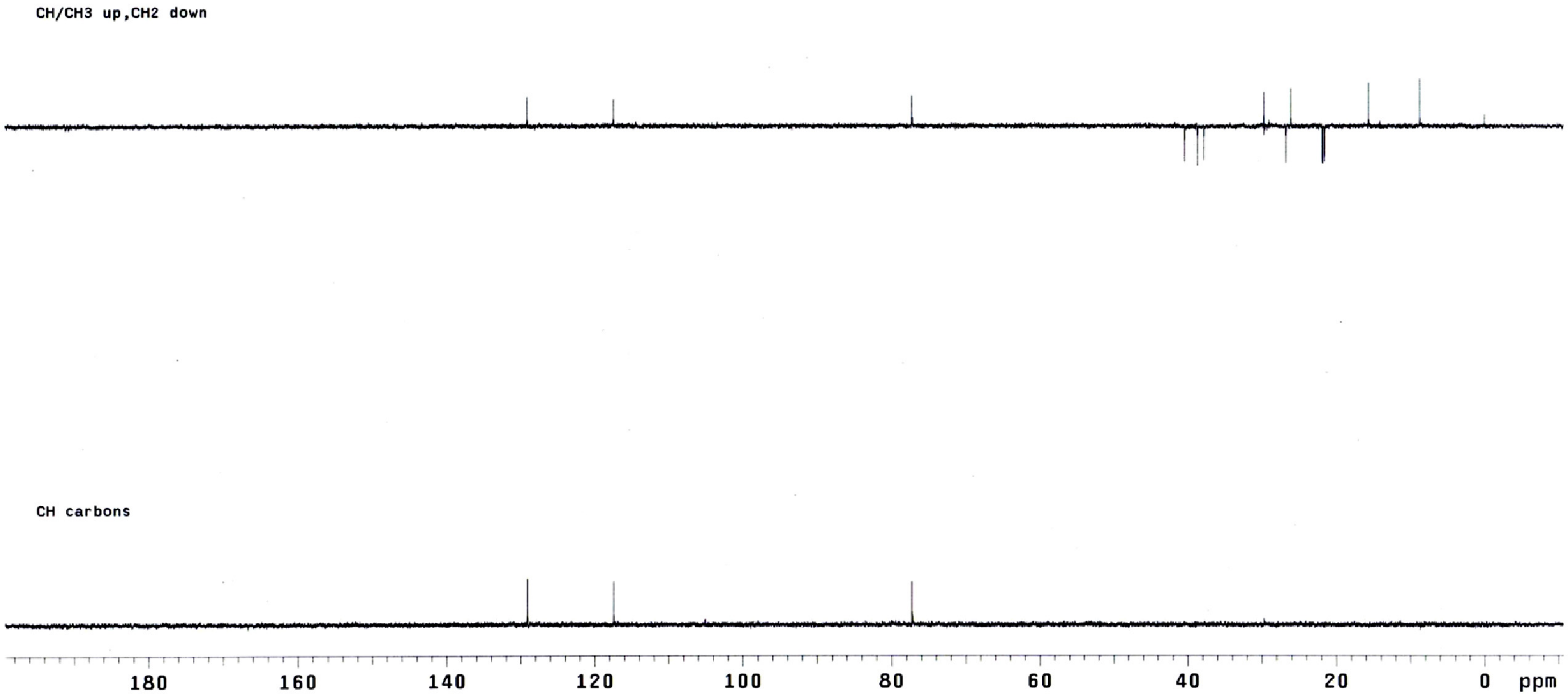

Figure S16. DEPT spectra of **2** in $CDCl_3$

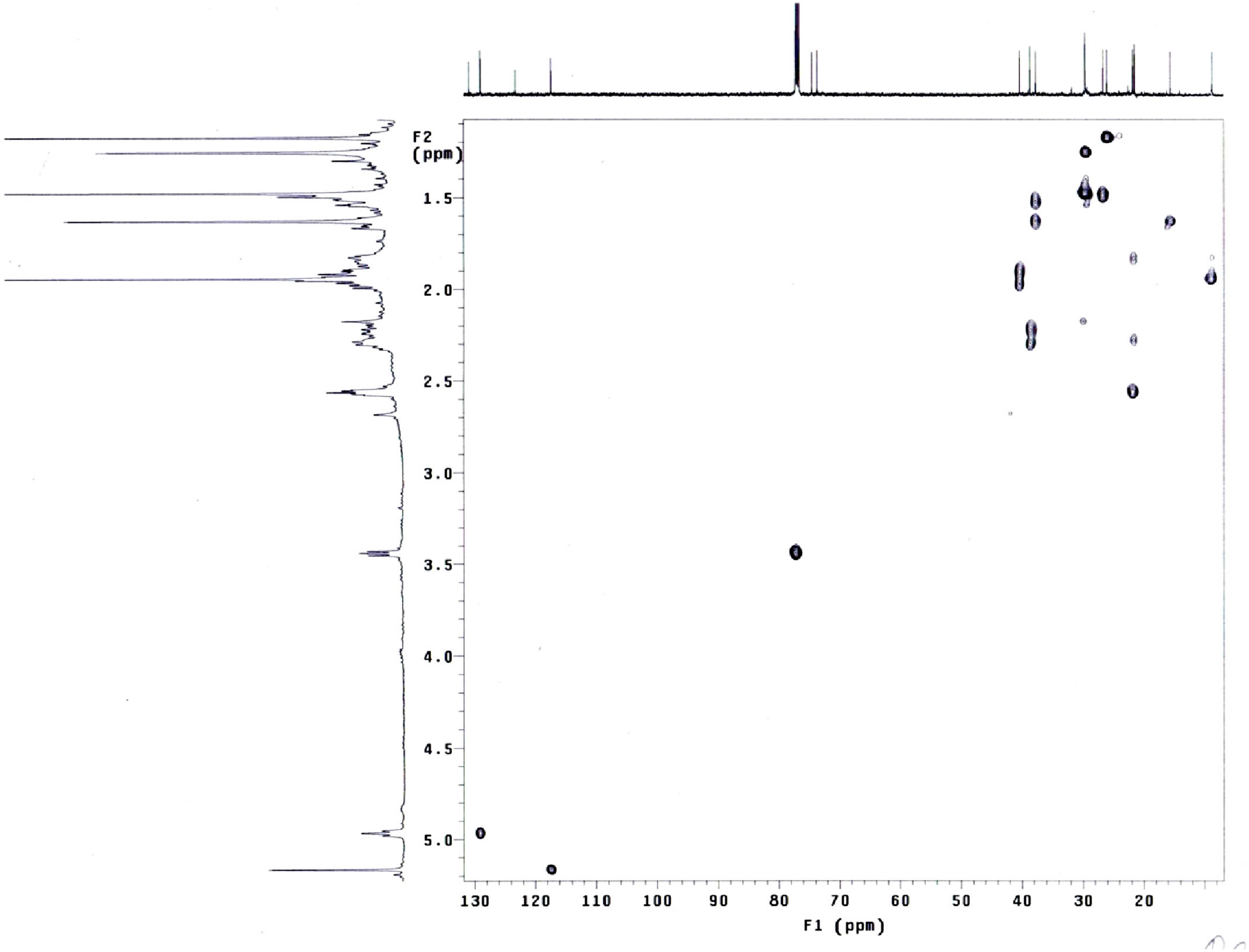

Figure S17. HMQC spectrum of **2** in $CDCl_3$

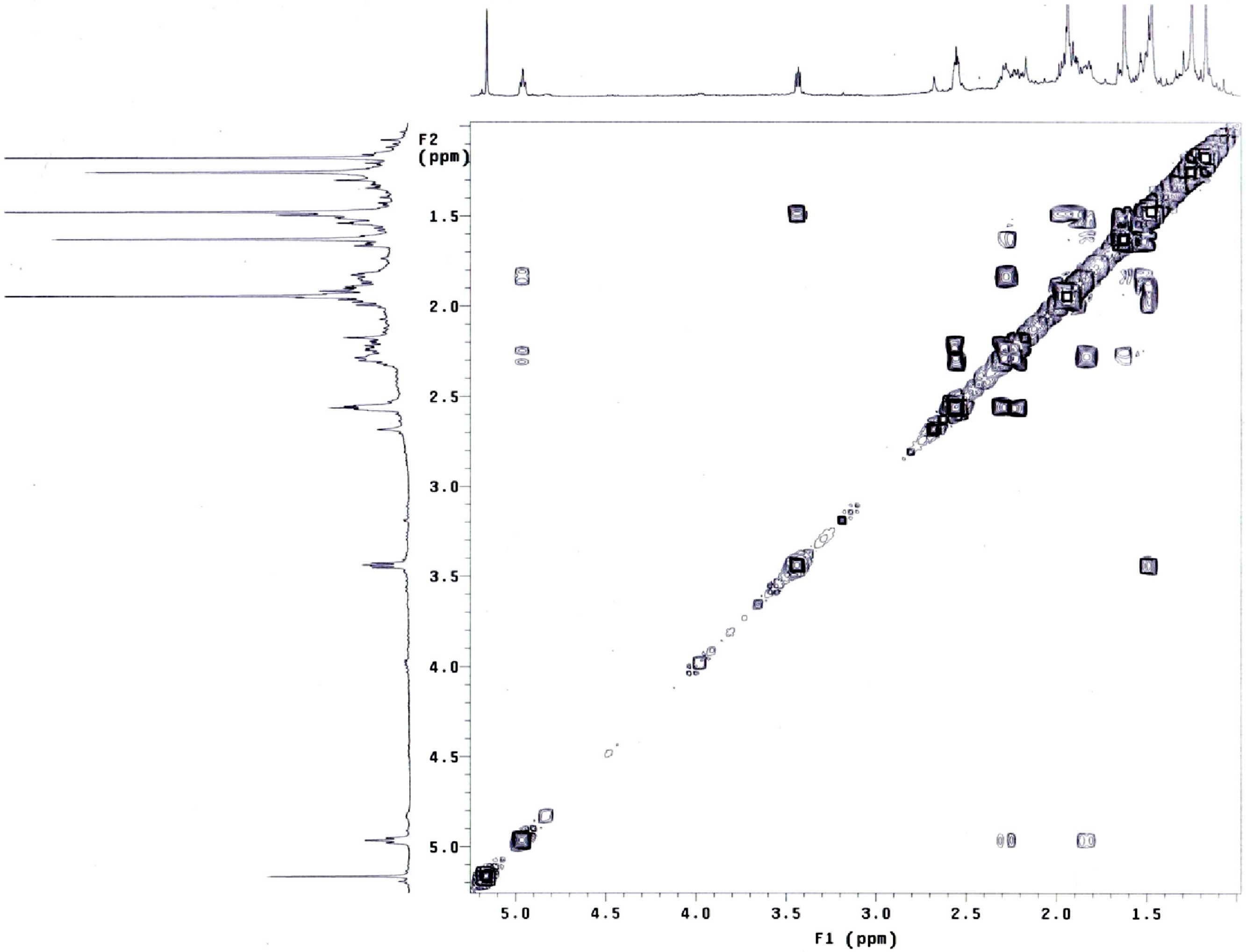

Figure S18. COSY spectrum of **2** in $CDCl_3$

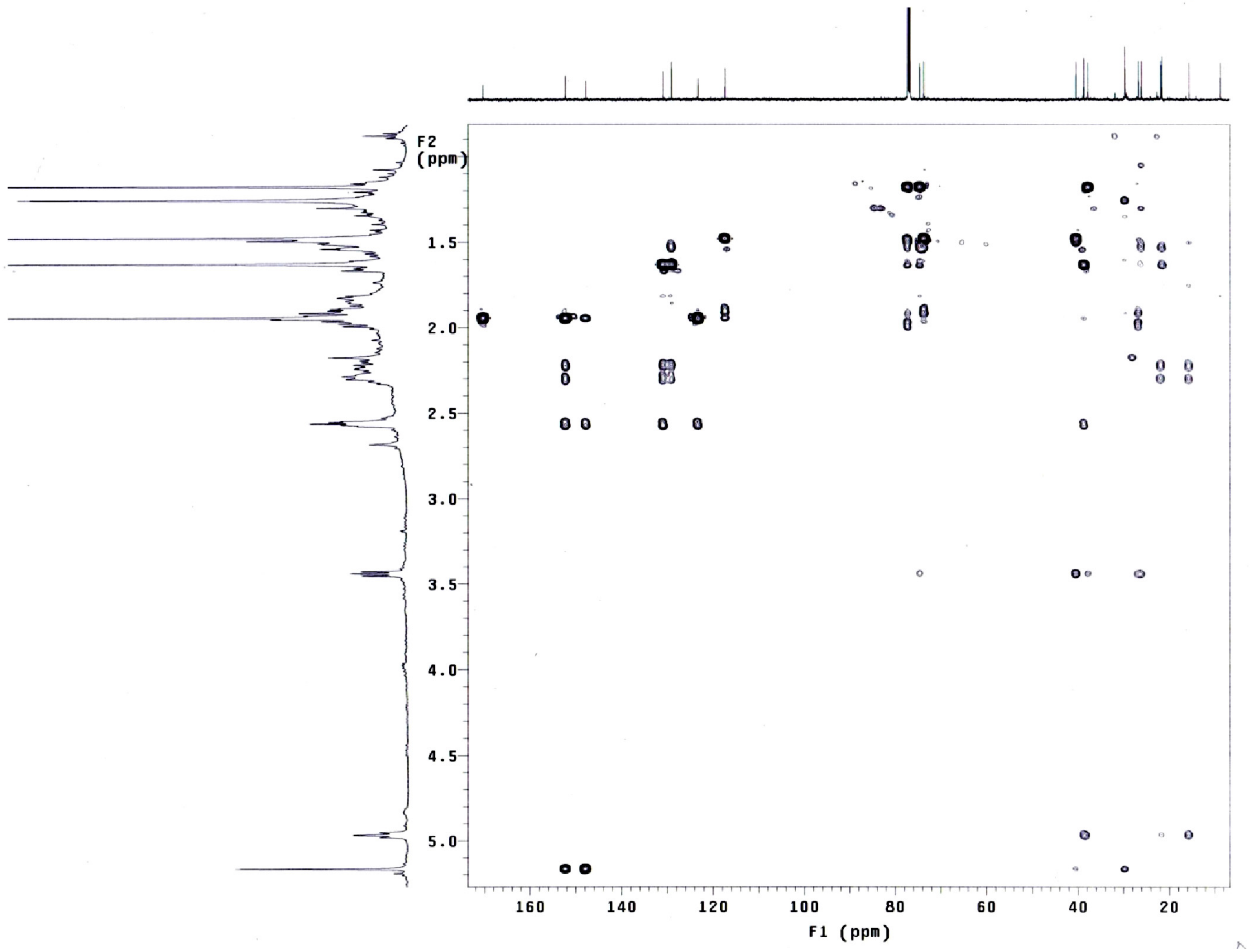

Figure S19. HMBC spectrum of **2** in $CDCl_3$

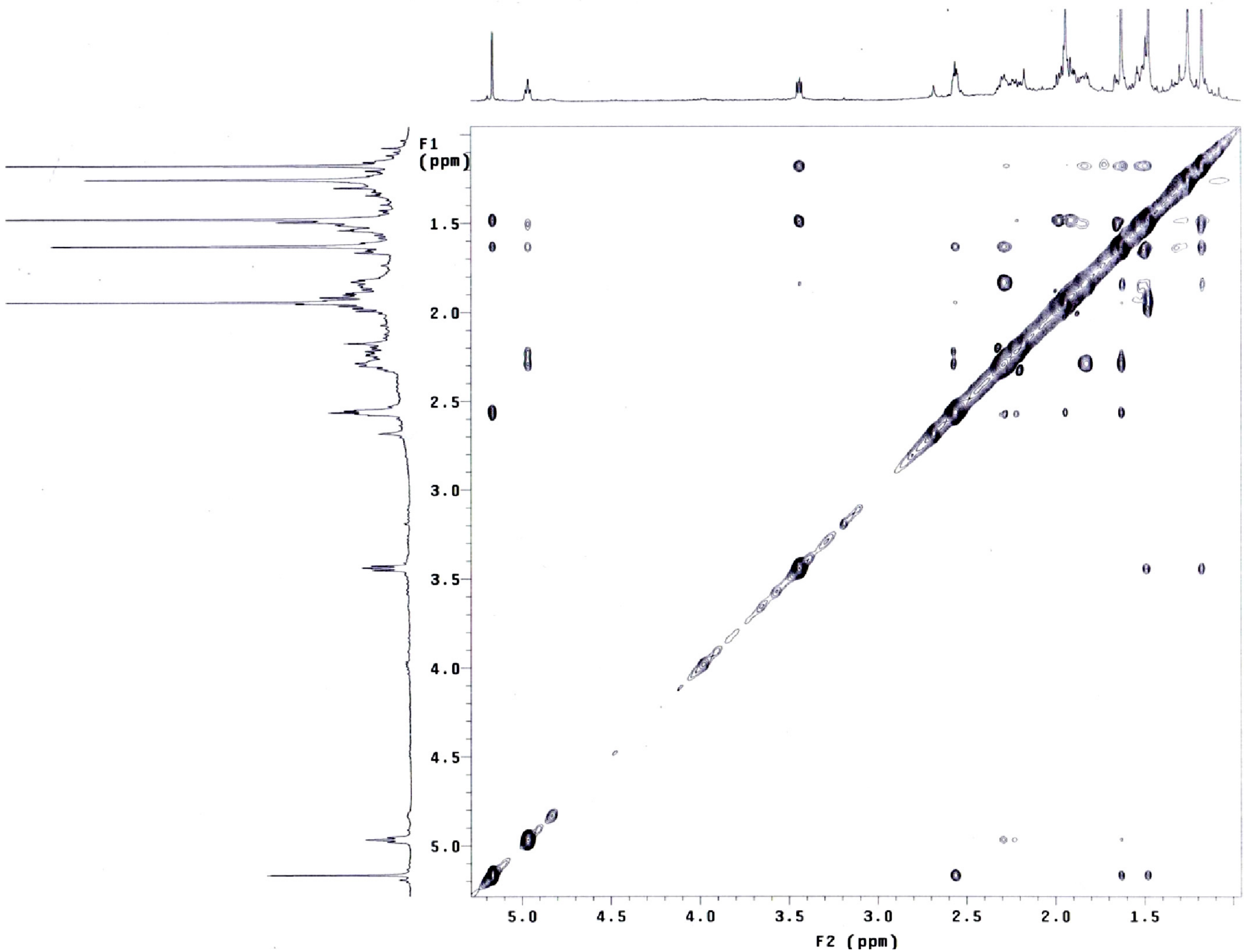

Figure S20. NOESY spectrum of **2** in $CDCl_3$

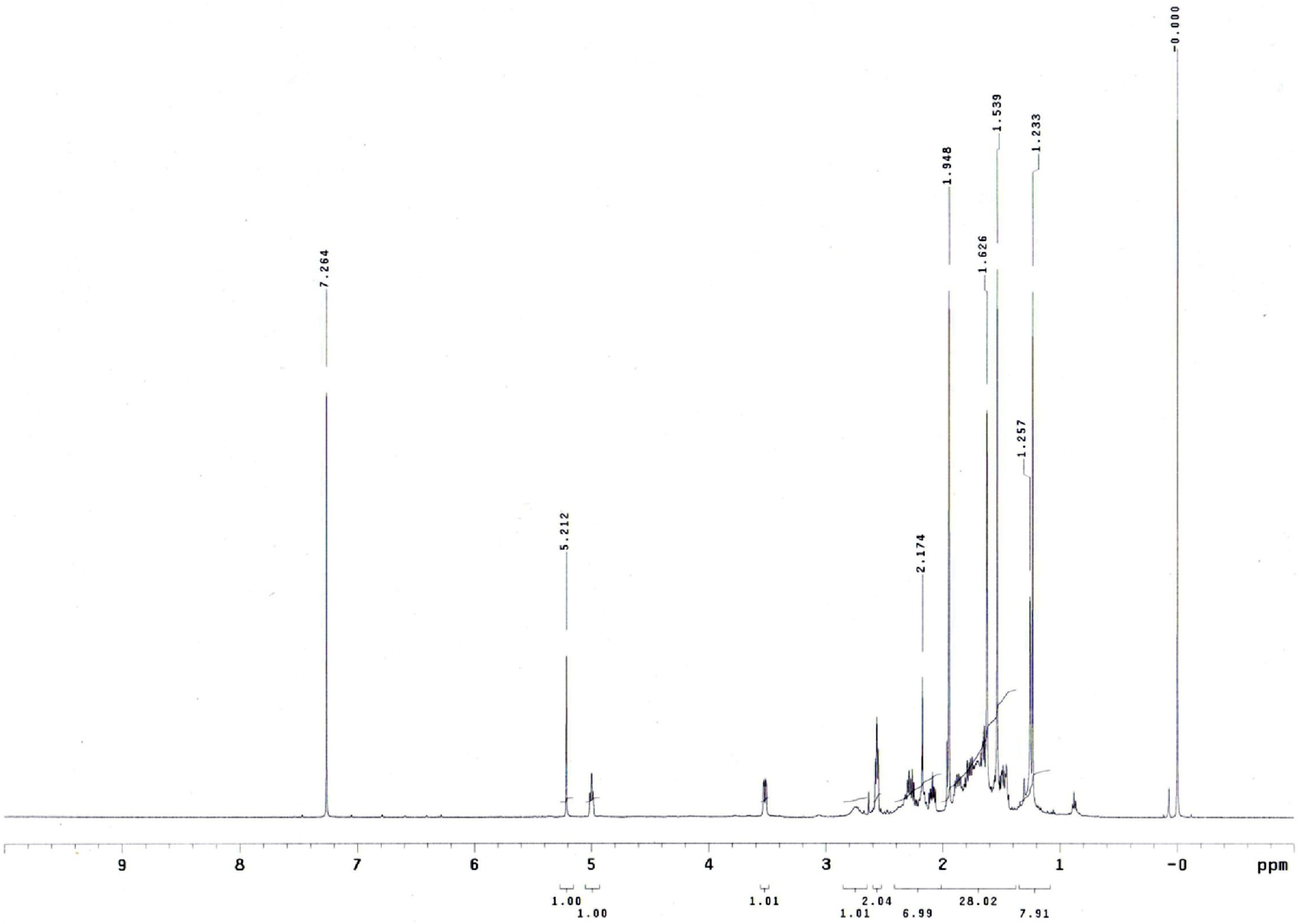

Figure S21. ^{1}H NMR spectrum of **3** in $CDCl_3$

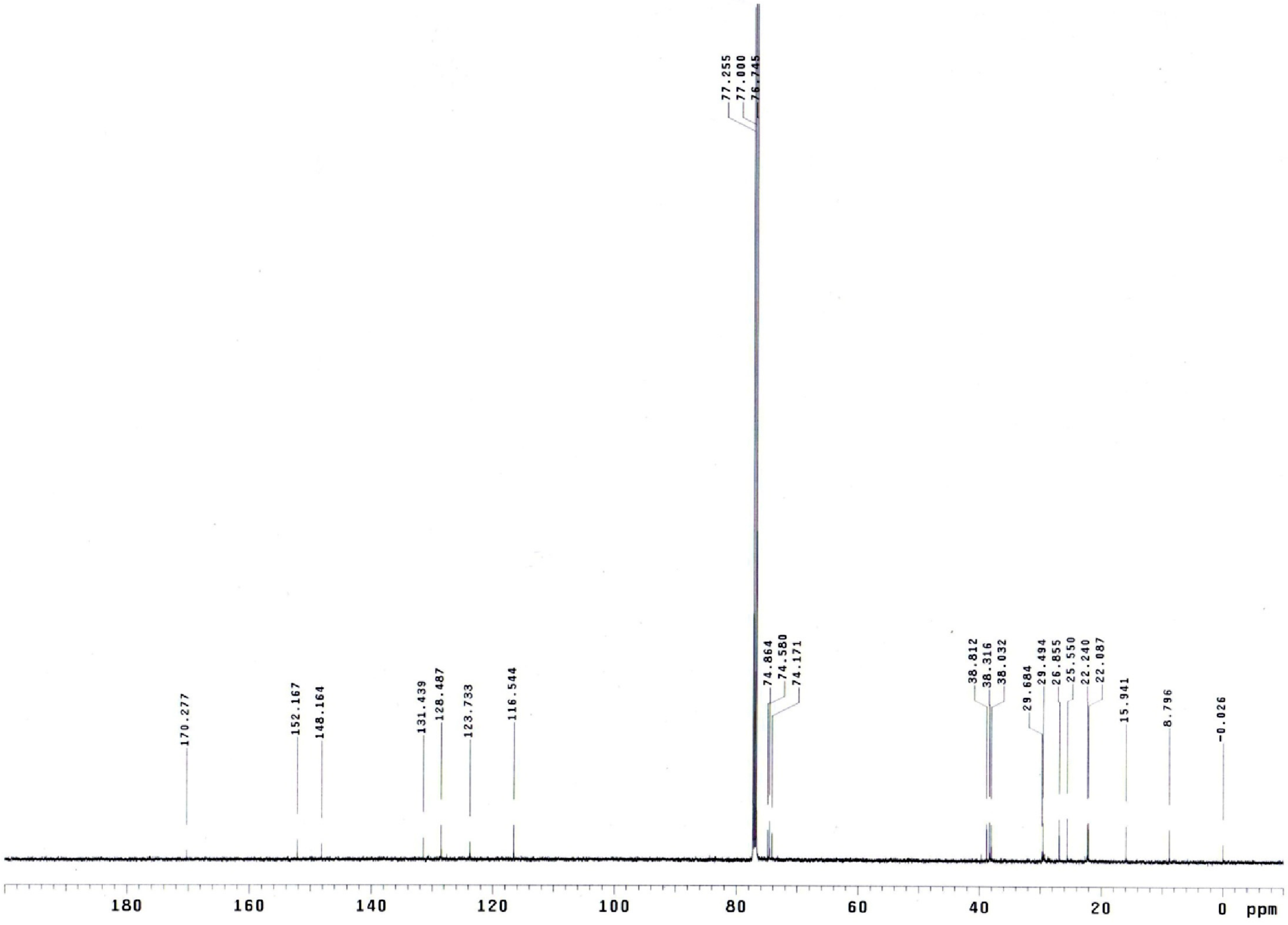

Figure S22. ^{13}C NMR spectrum of **3** in $CDCl_3$

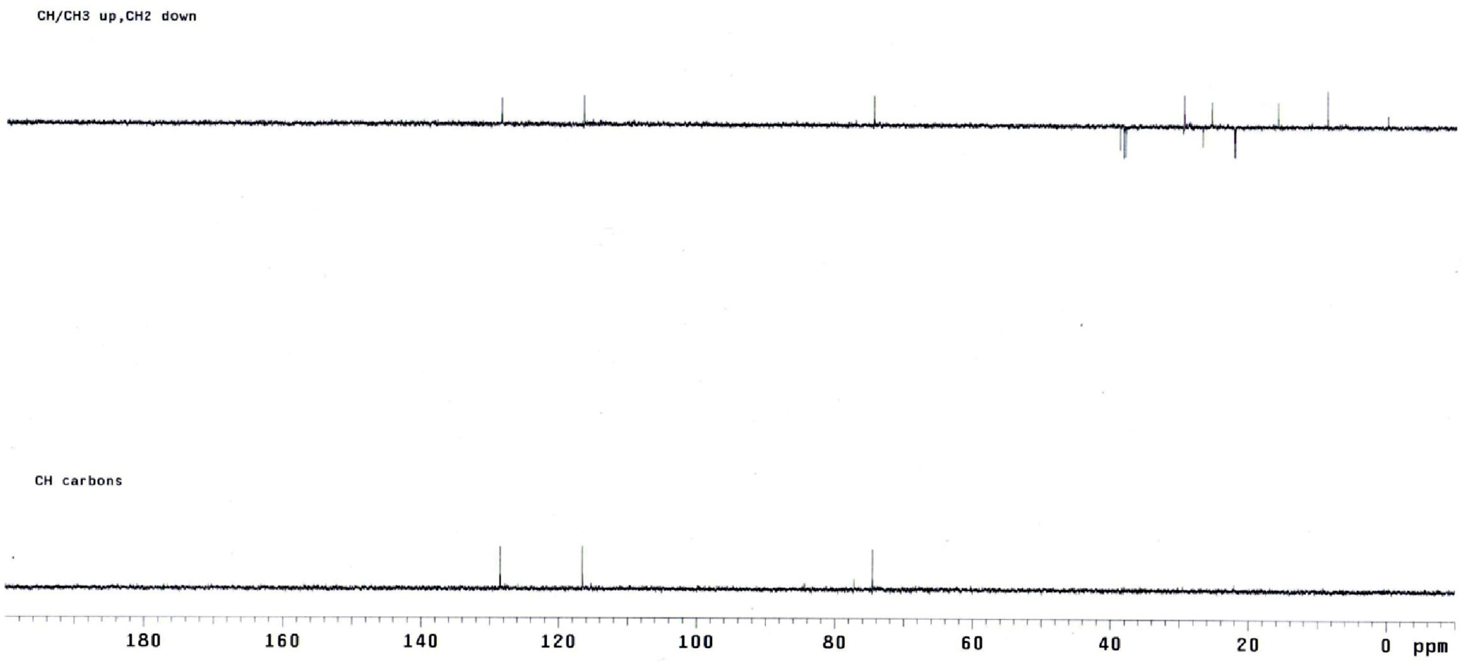

Figure S23. DEPT spectra of **3** in $CDCl_3$

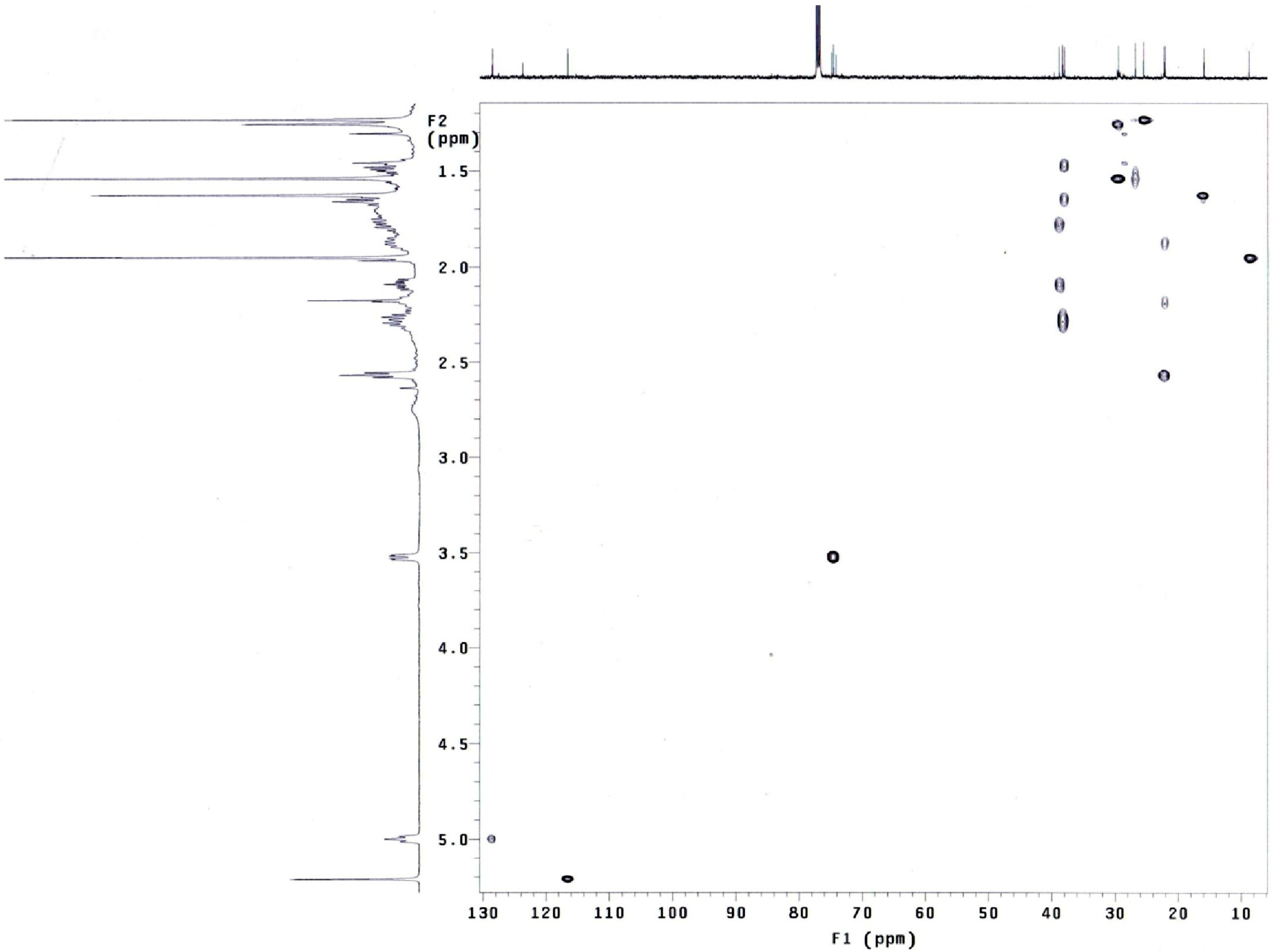

Figure S24. HMQC spectrum of **3** in $CDCl_3$

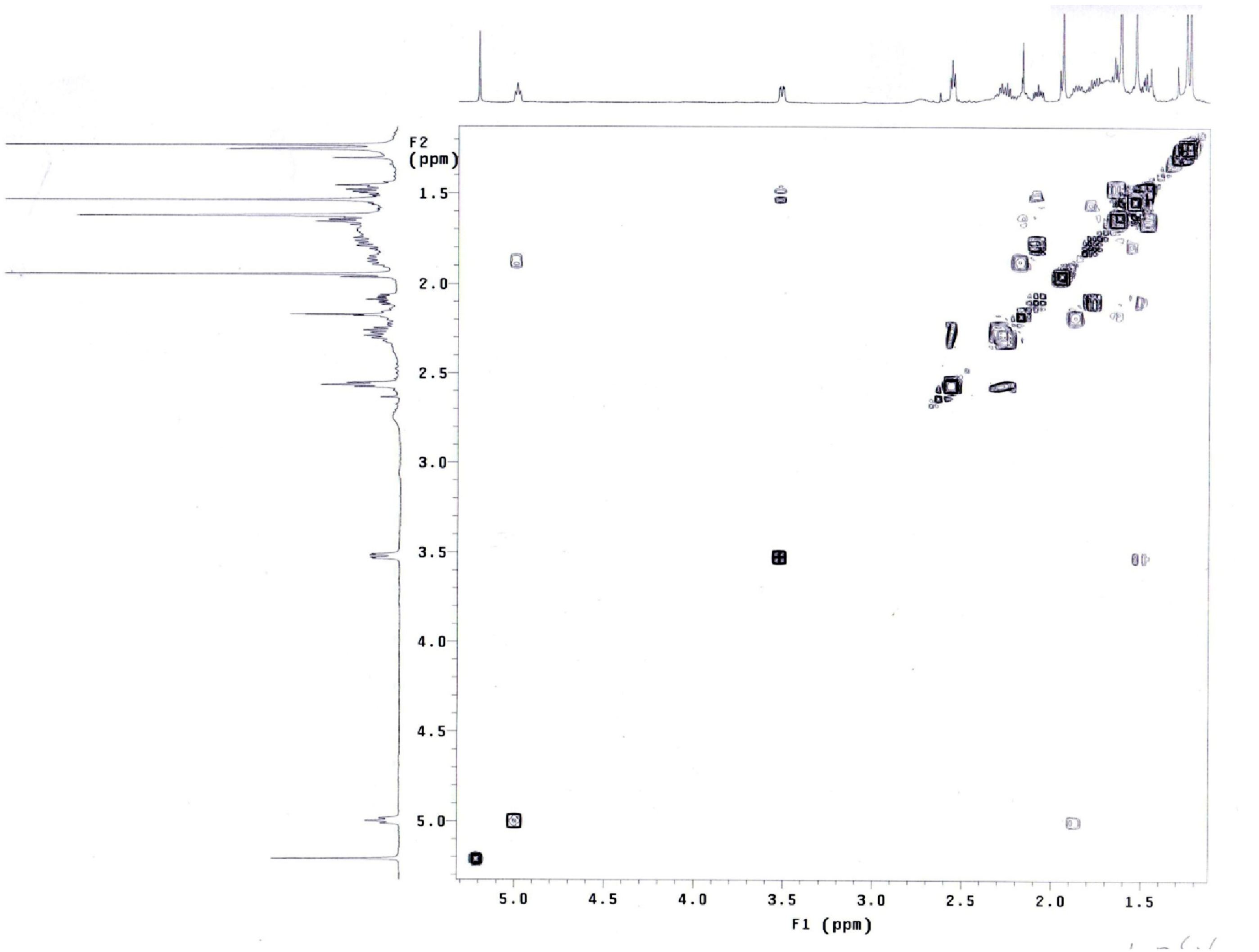

Figure S25. COSY spectrum of **3** in $CDCl_3$

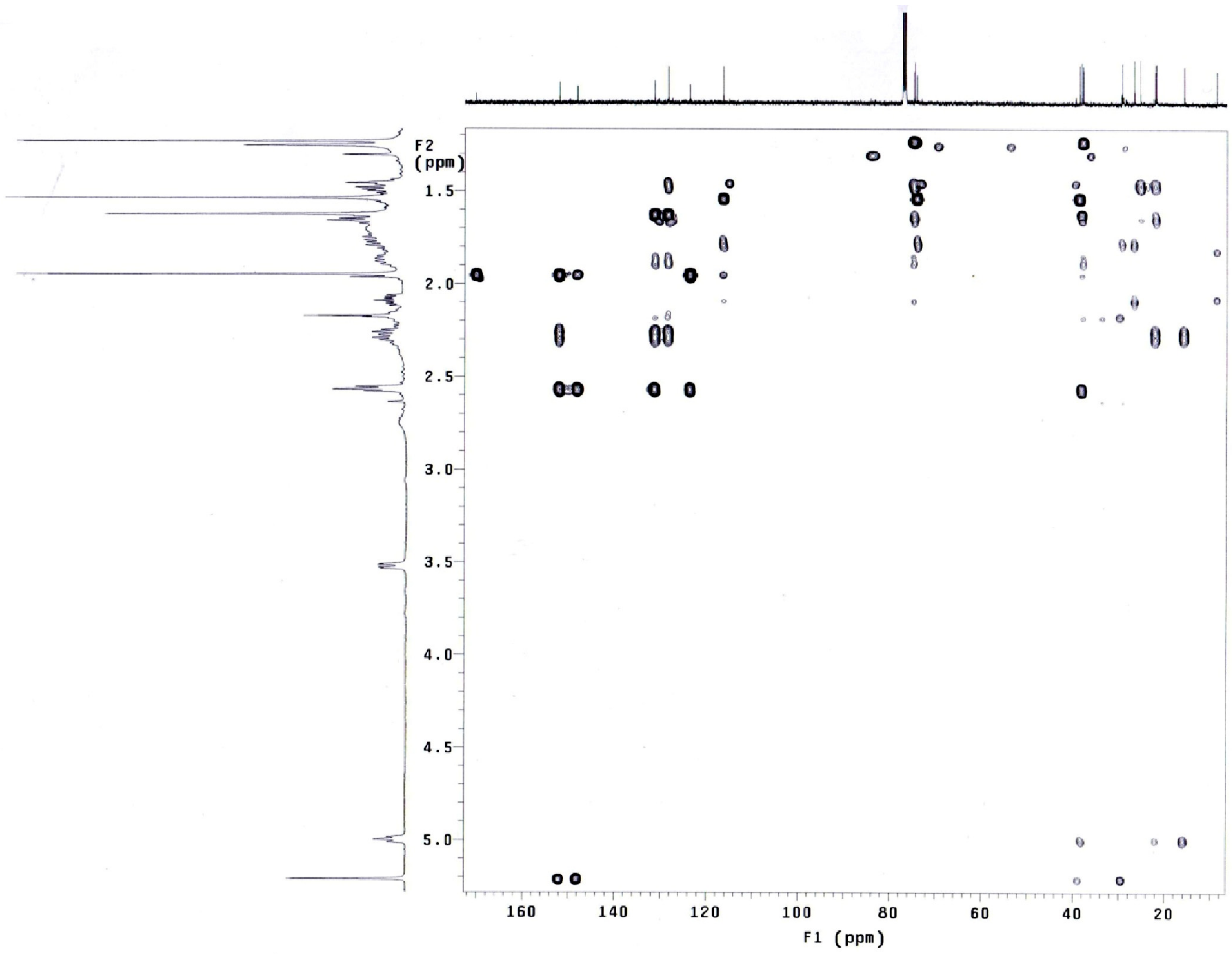

Figure S26. HMBC spectrum of **3** in $CDCl_3$

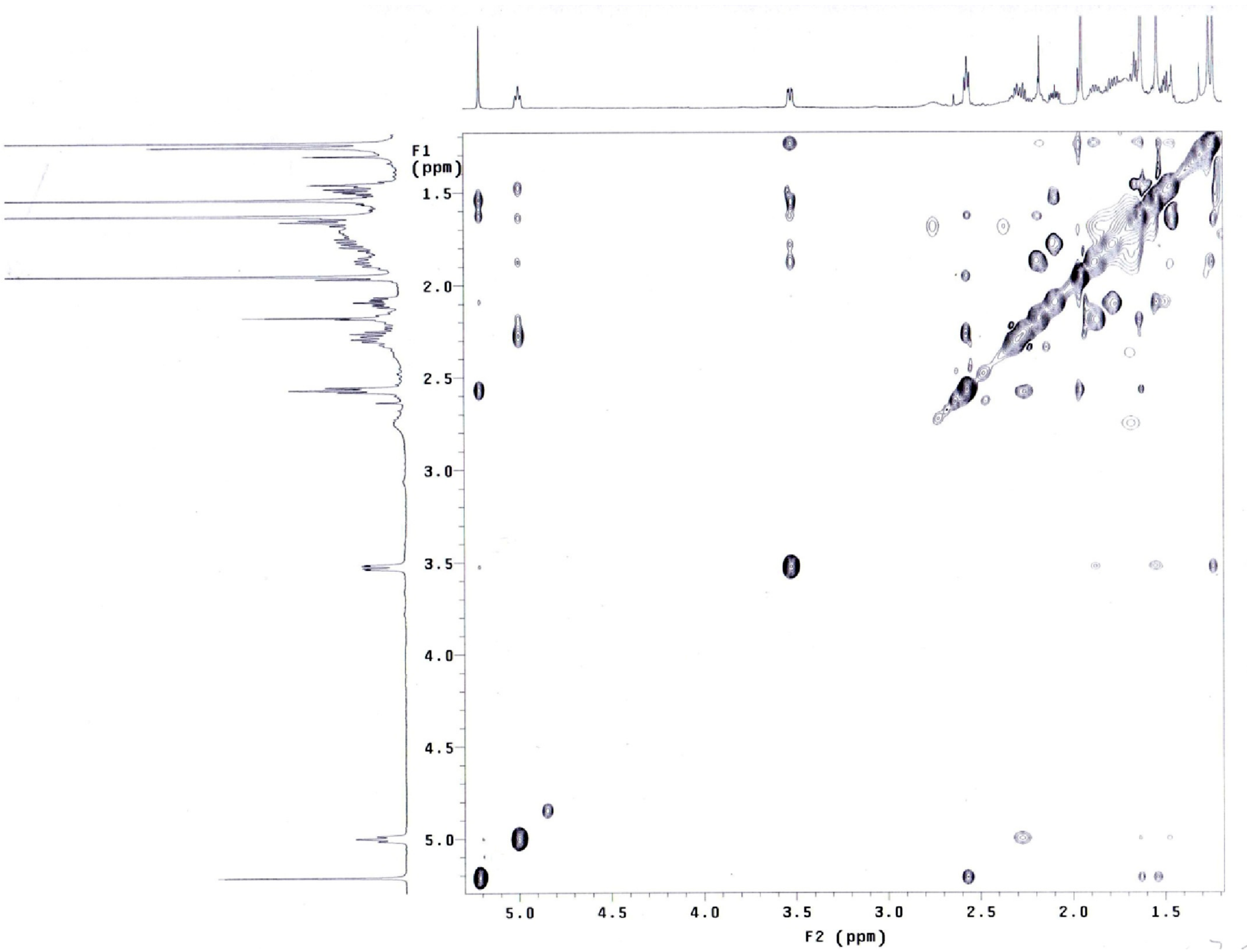

Figure S27. NOESY spectrum of **3** in $CDCl_3$

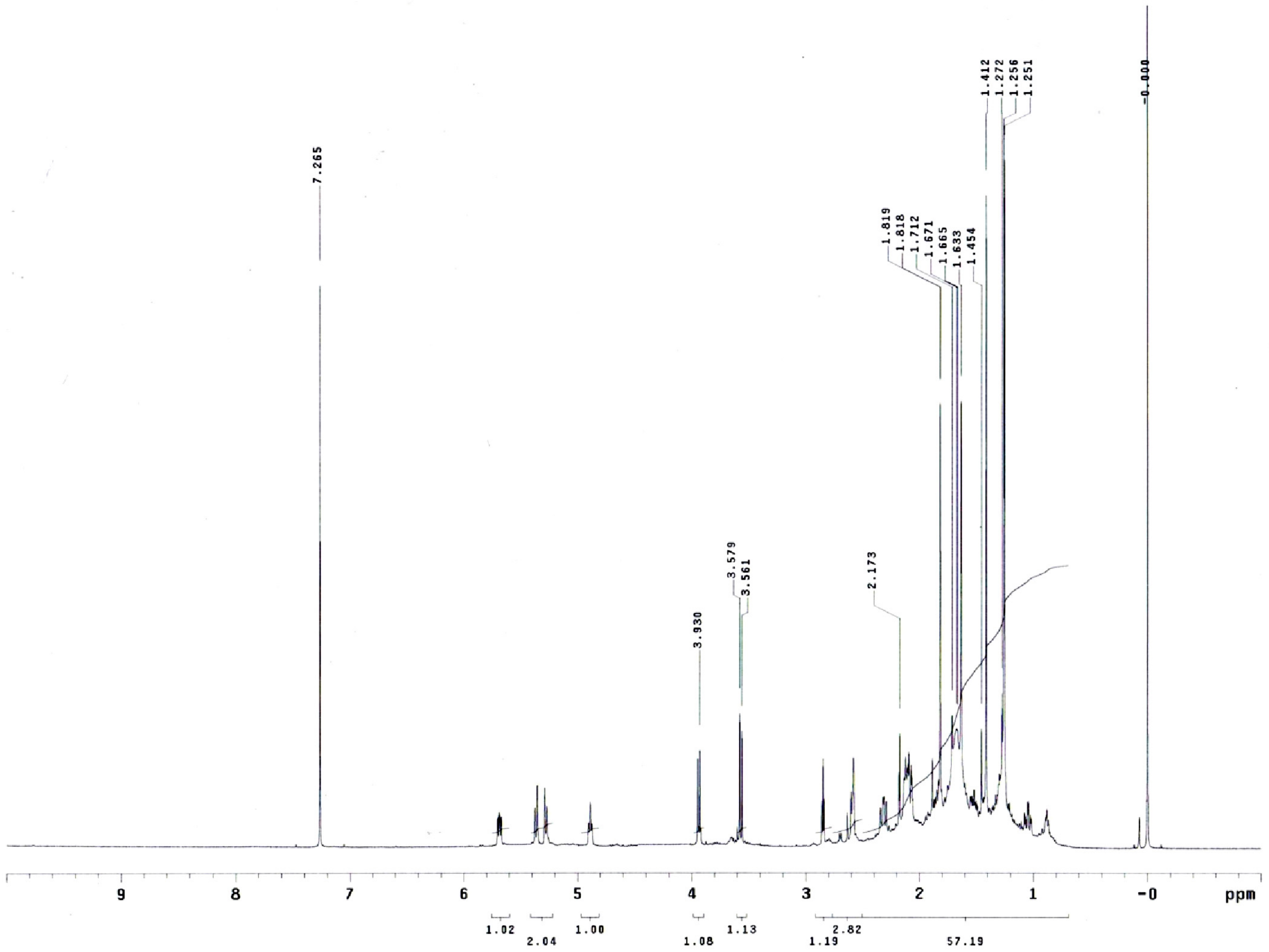

Figure S28. ^{1}H NMR spectrum of **4** in $CDCl_3$

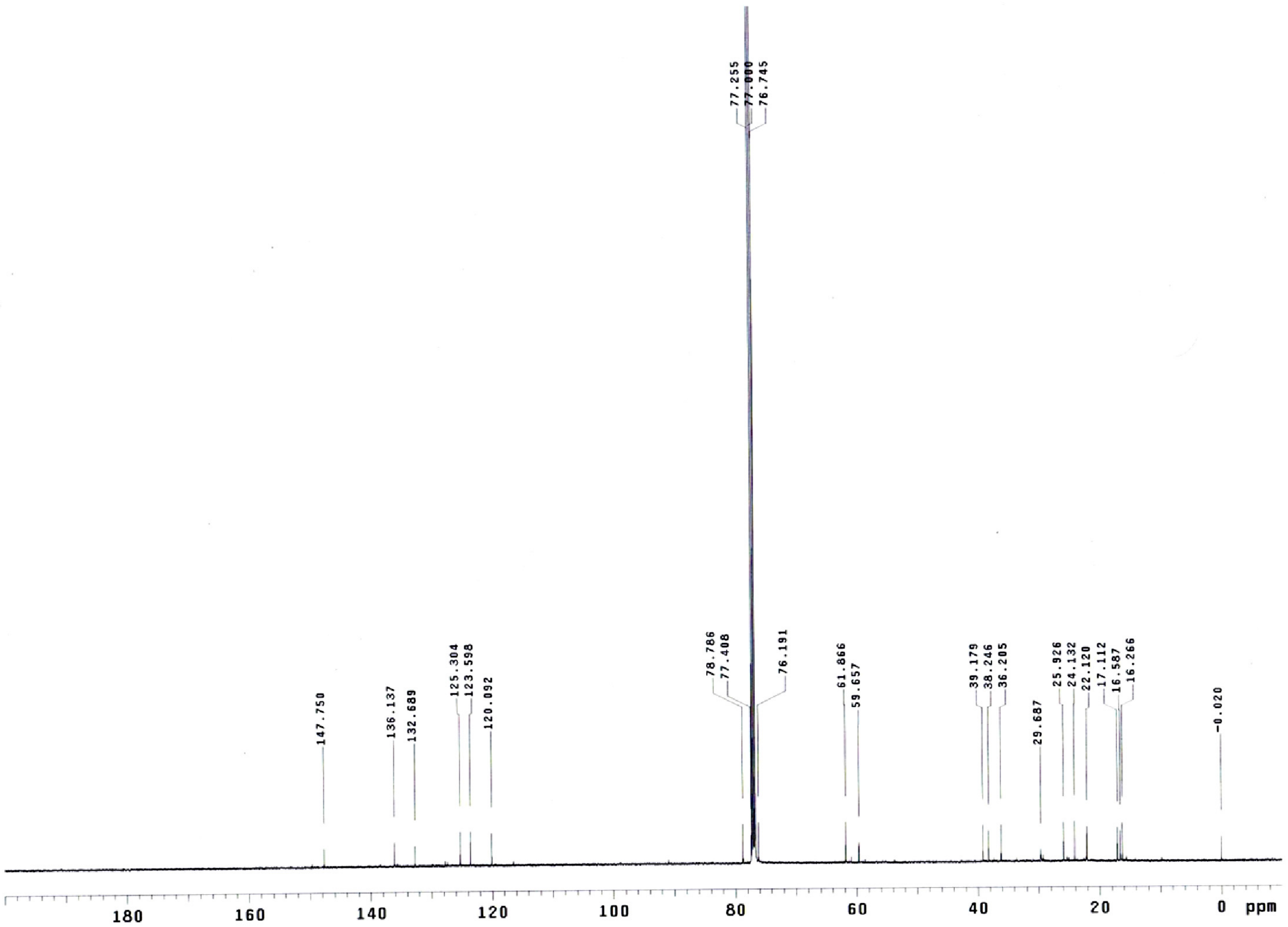

Figure S29. ^{13}C NMR spectrum of **4** in $CDCl_3$

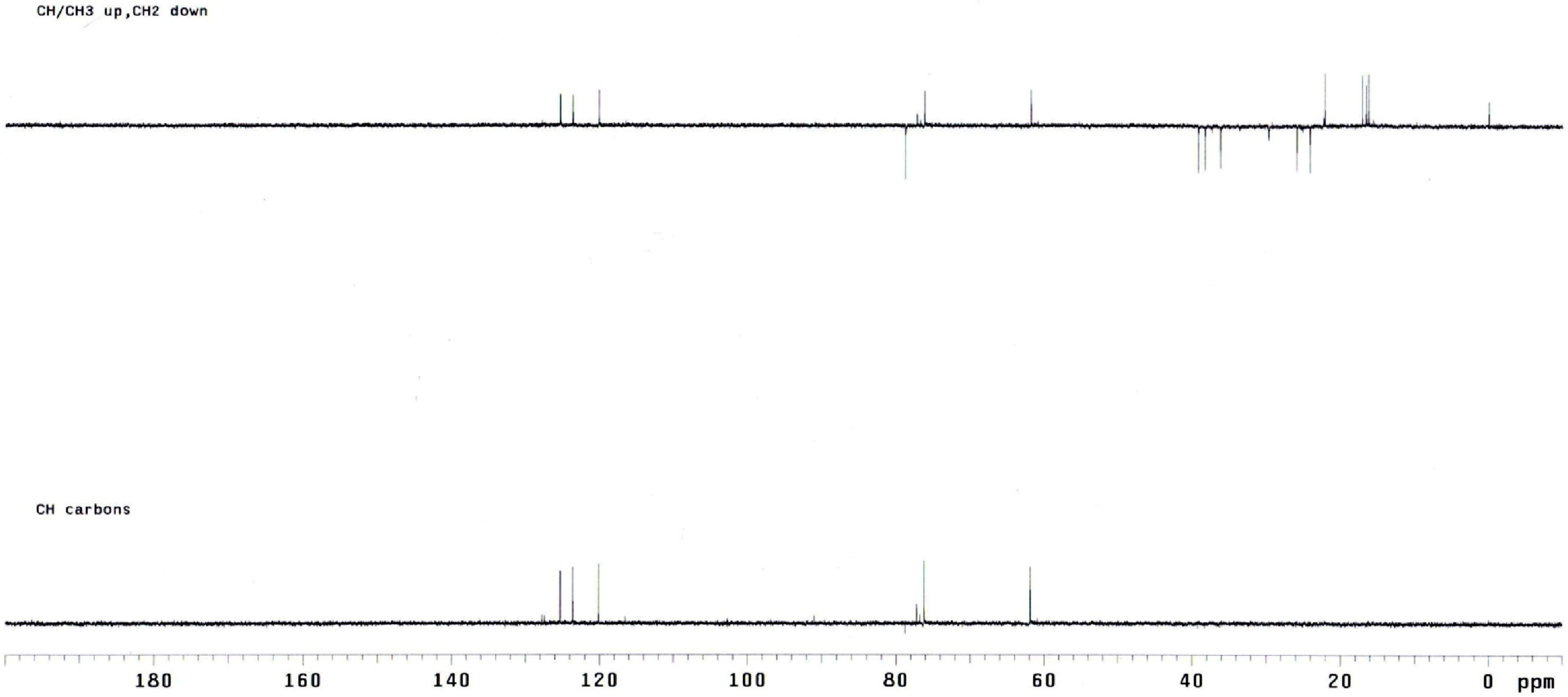

Figure S30. DEPT spectra of **4** in $CDCl_3$

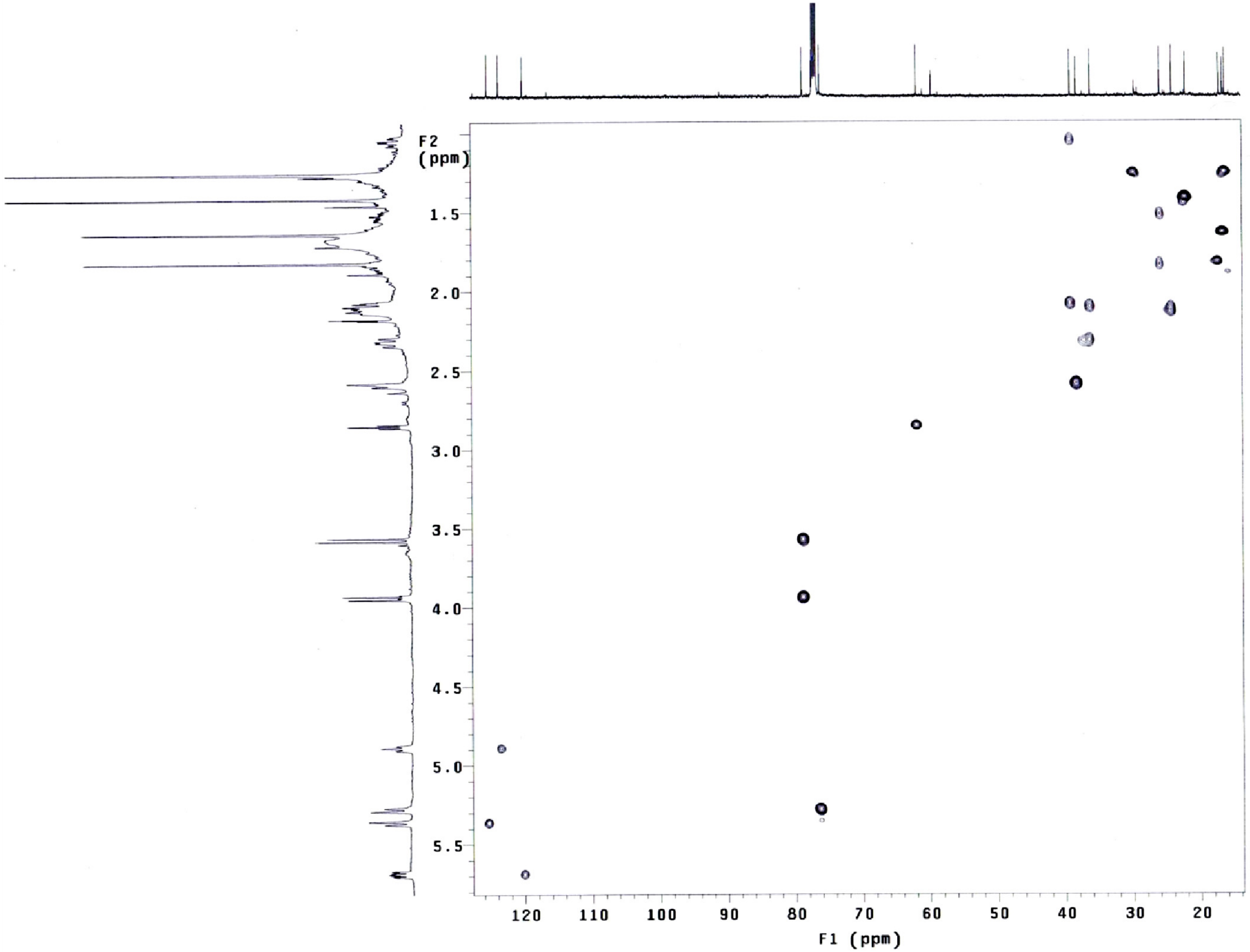

Figure S31. HMQC spectrum of **4** in $CDCl_3$

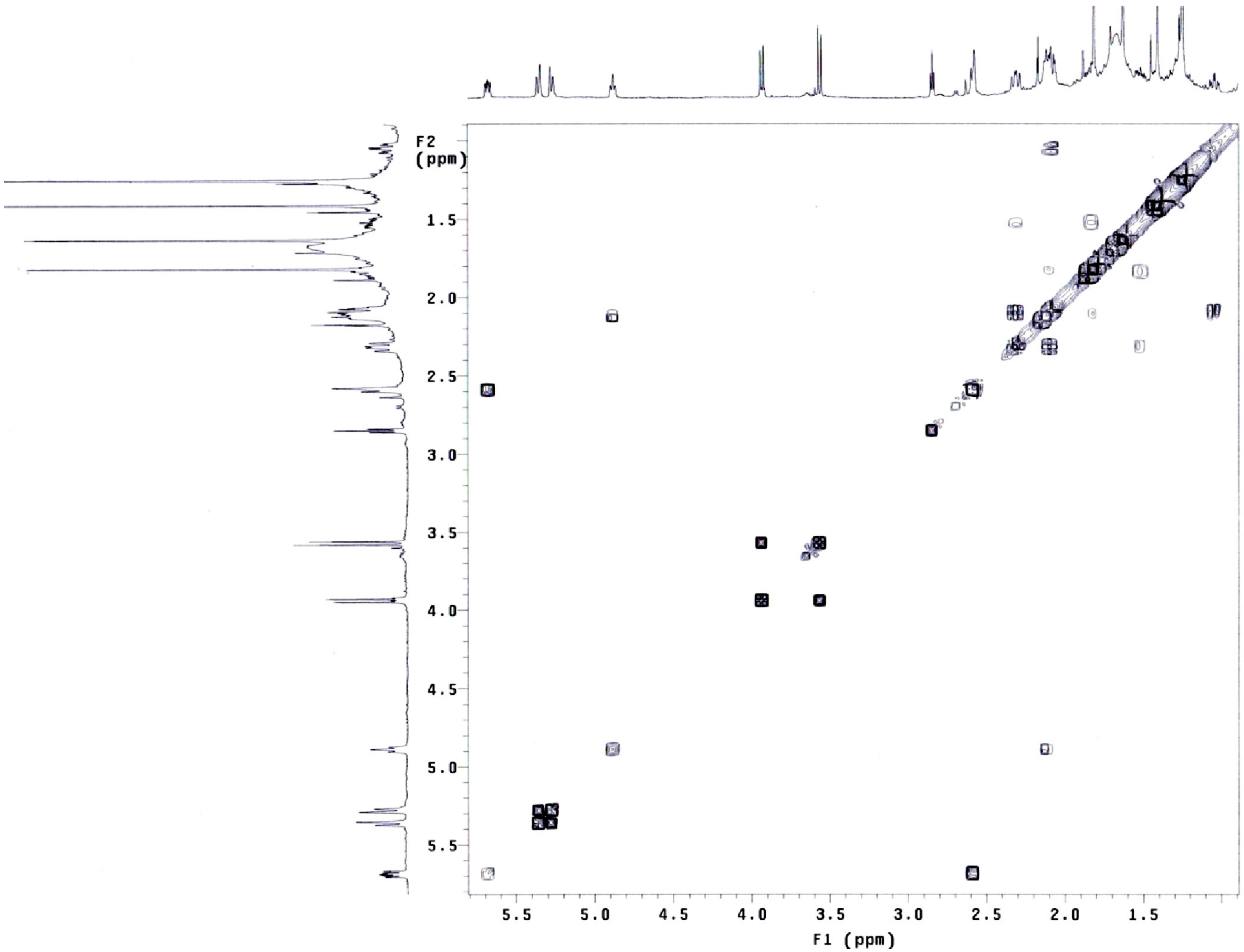

Figure S32. COSY spectrum of **4** in $CDCl_3$

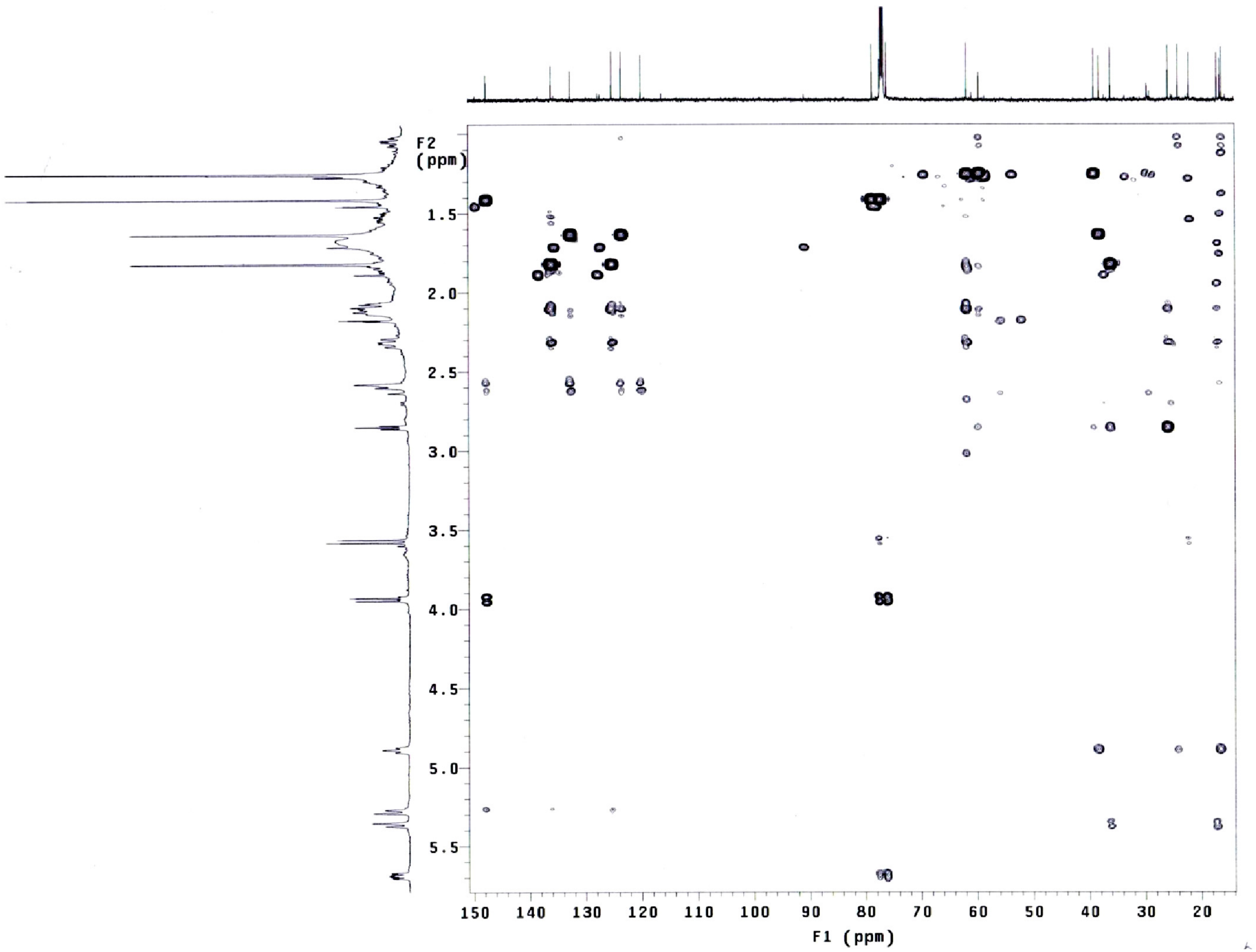

Figure S33. HMBC spectrum of **4** in $CDCl_3$

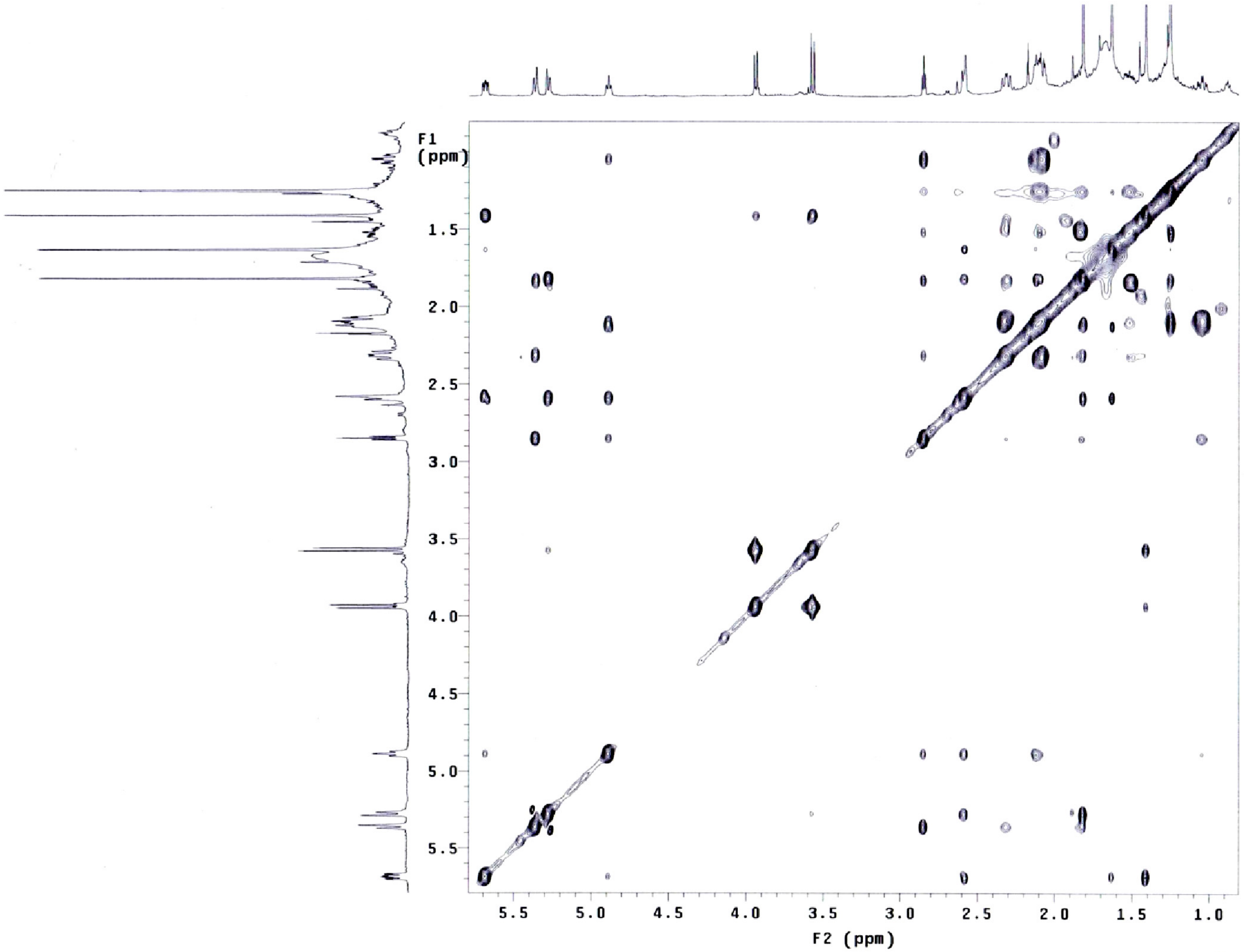

Figure S34. NOESY spectrum of **4** in $CDCl_3$

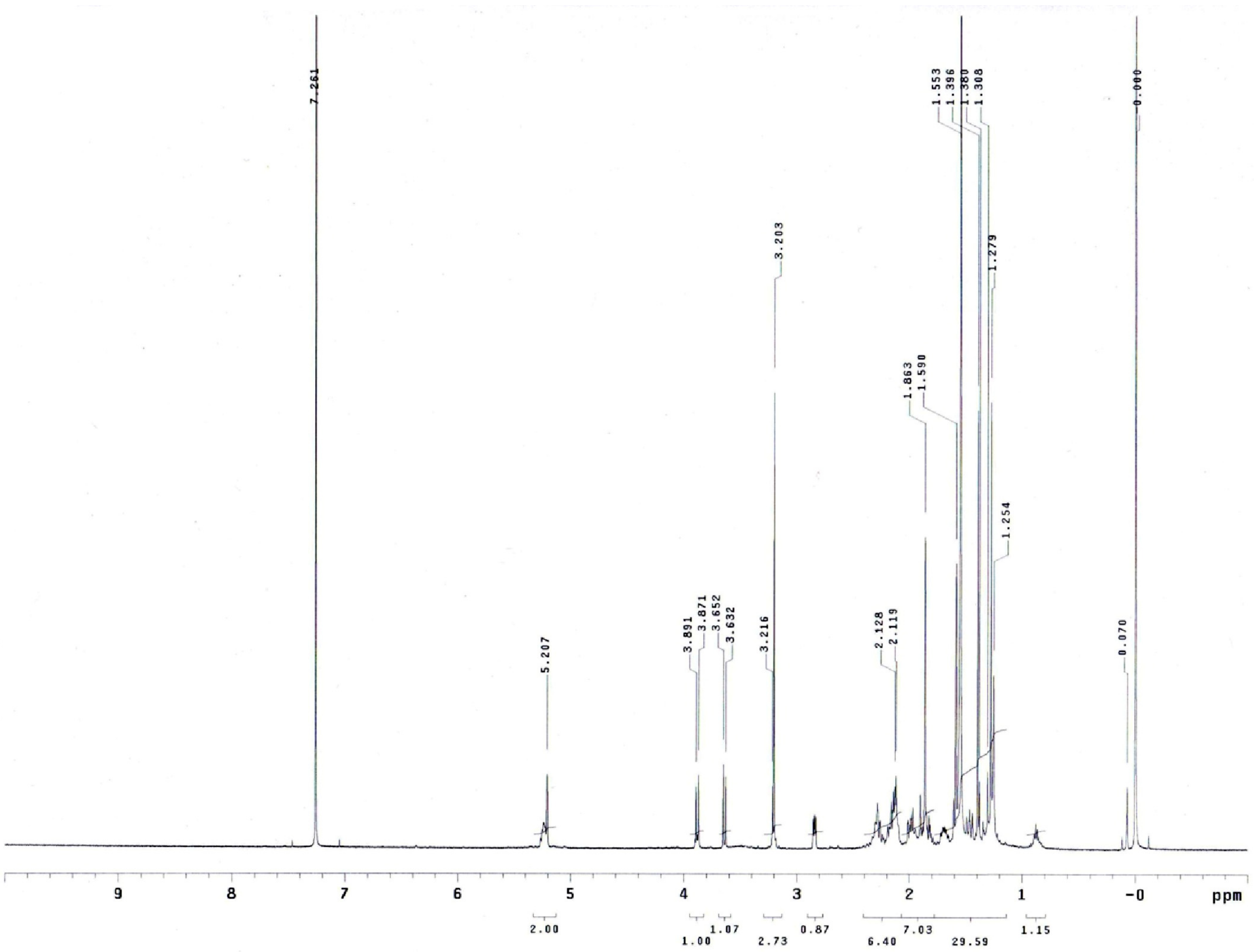

Figure S35. ^{1}H NMR spectrum of **5** in $CDCl_3$

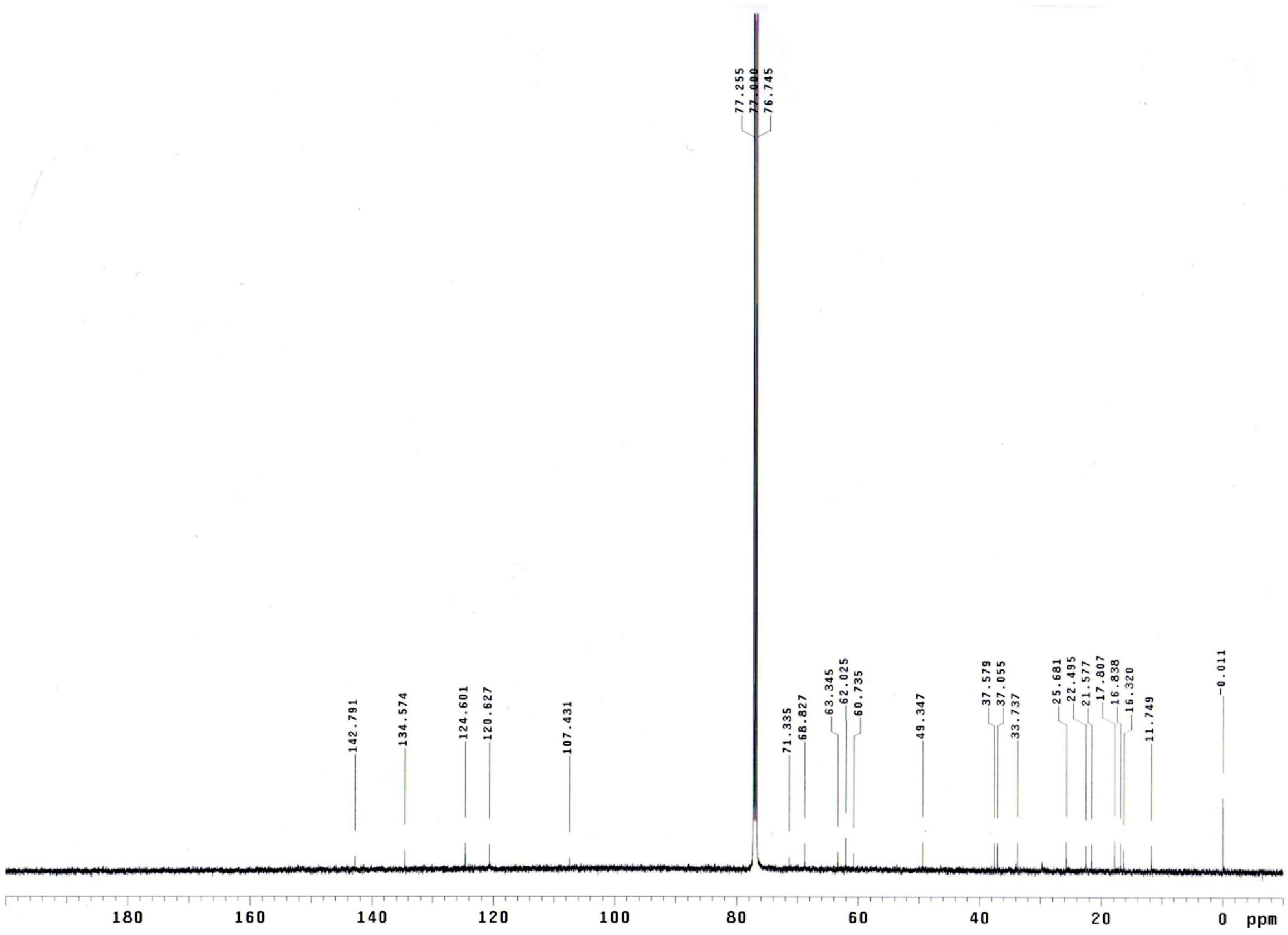

Figure S36. ^{13}C NMR spectrum of **5** in $CDCl_3$

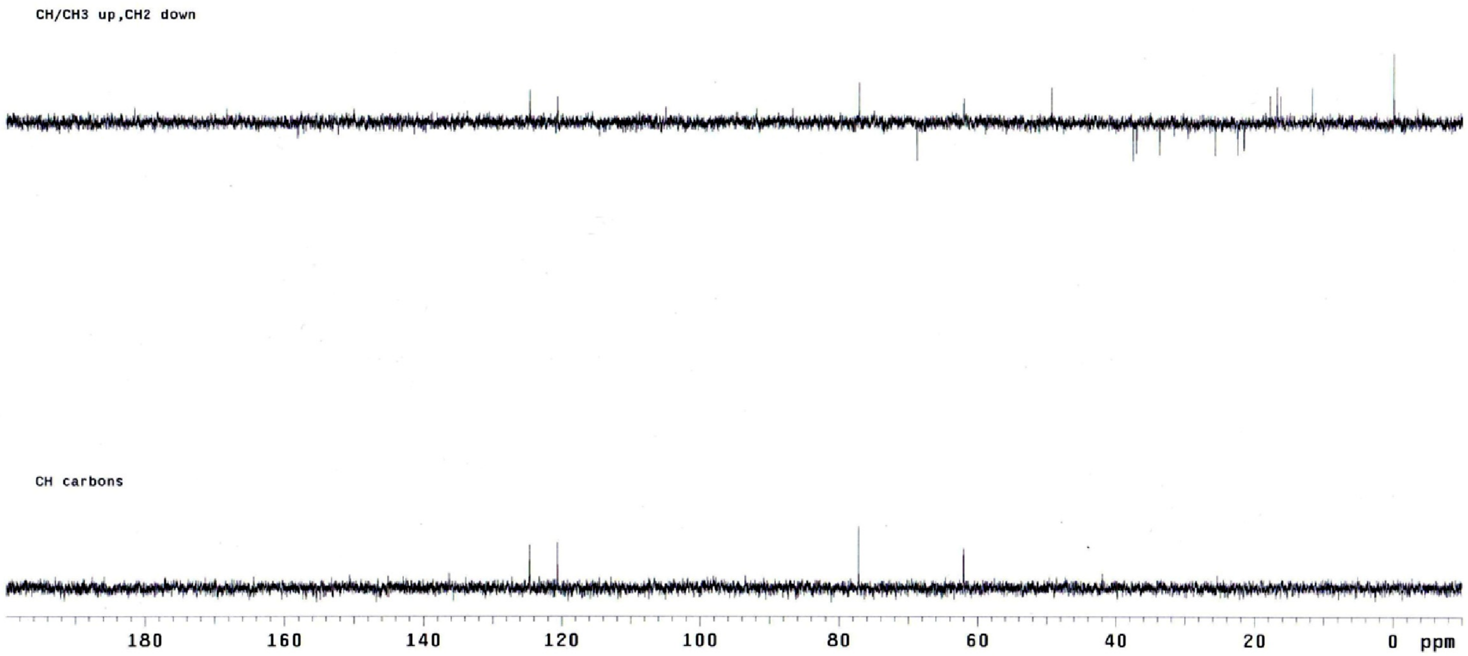

Figure S37. DEPT spectra of **5** in $CDCl_3$

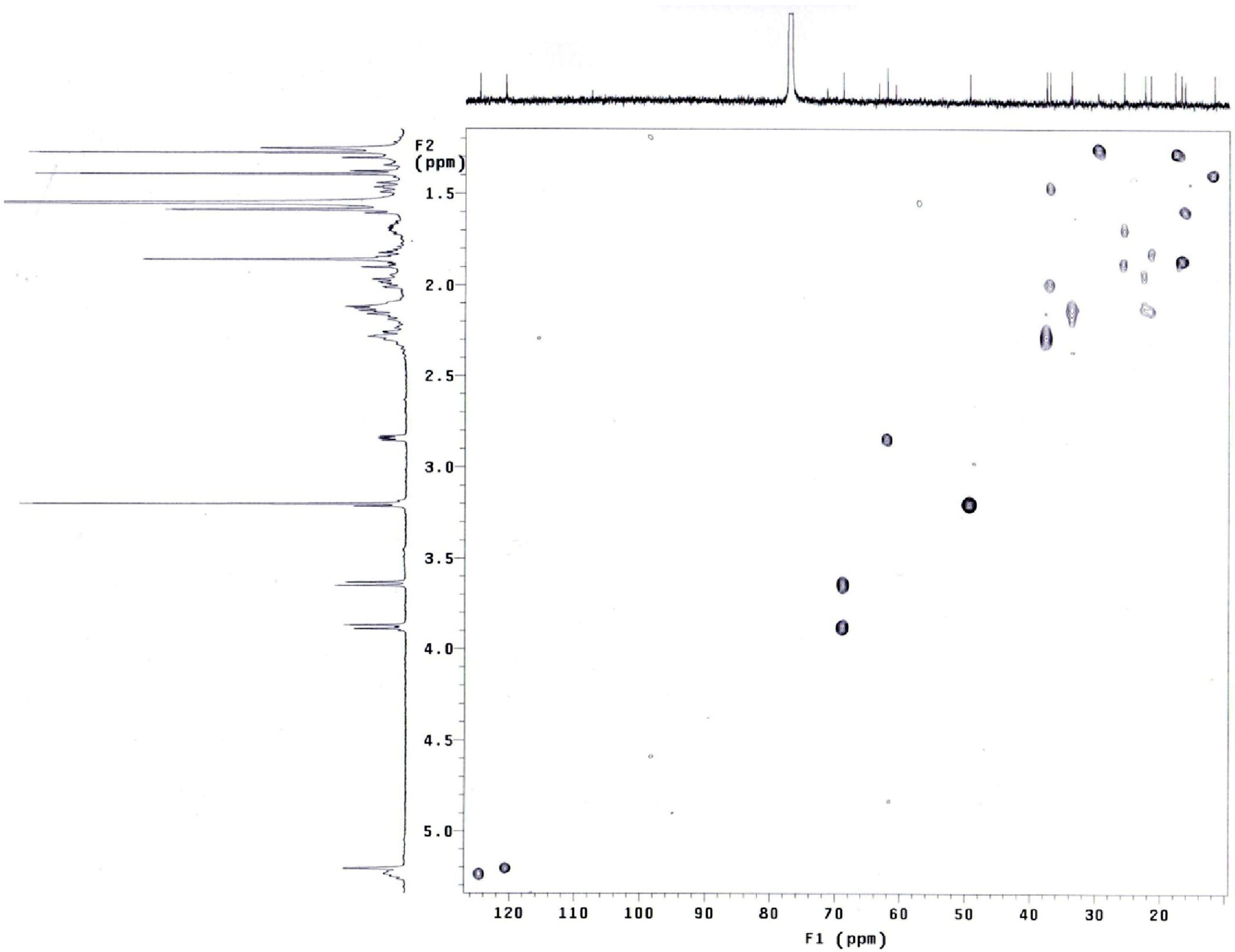

Figure S38. HMQC spectrum of **5** in $CDCl_3$

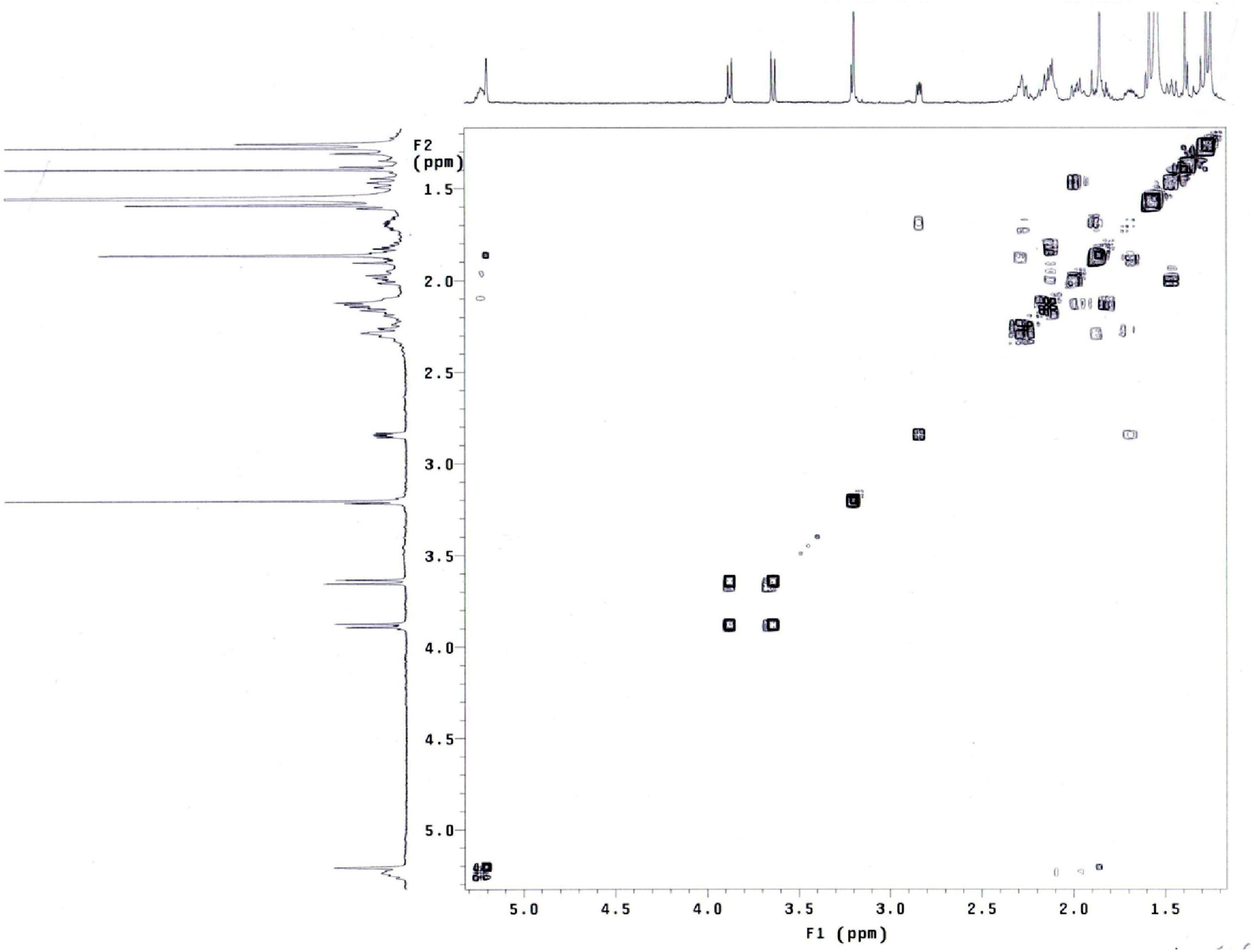

Figure S39. COSY spectrum of **5** in $CDCl_3$

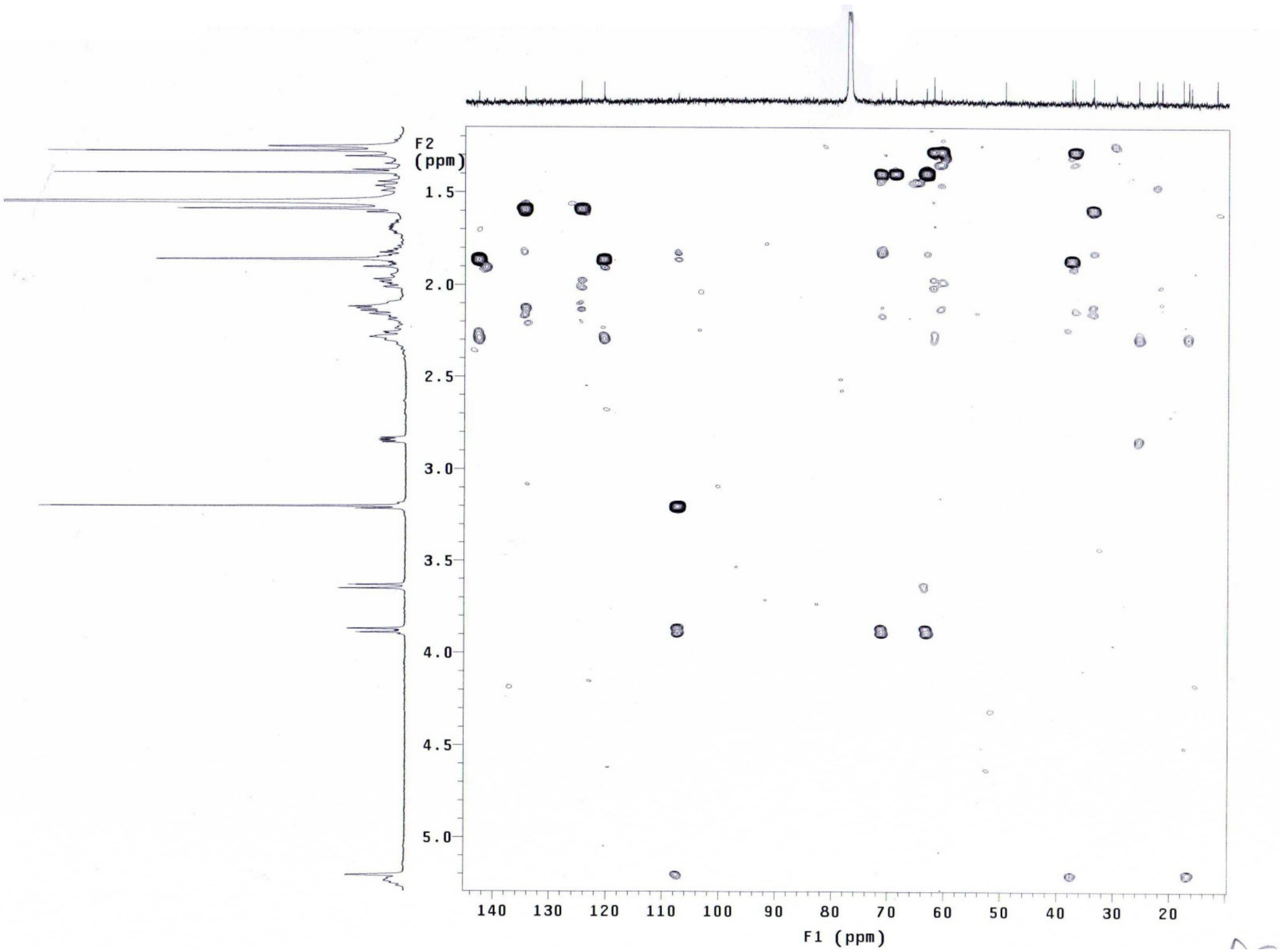

Figure S40. HMBC spectrum of **5** in $CDCl_3$

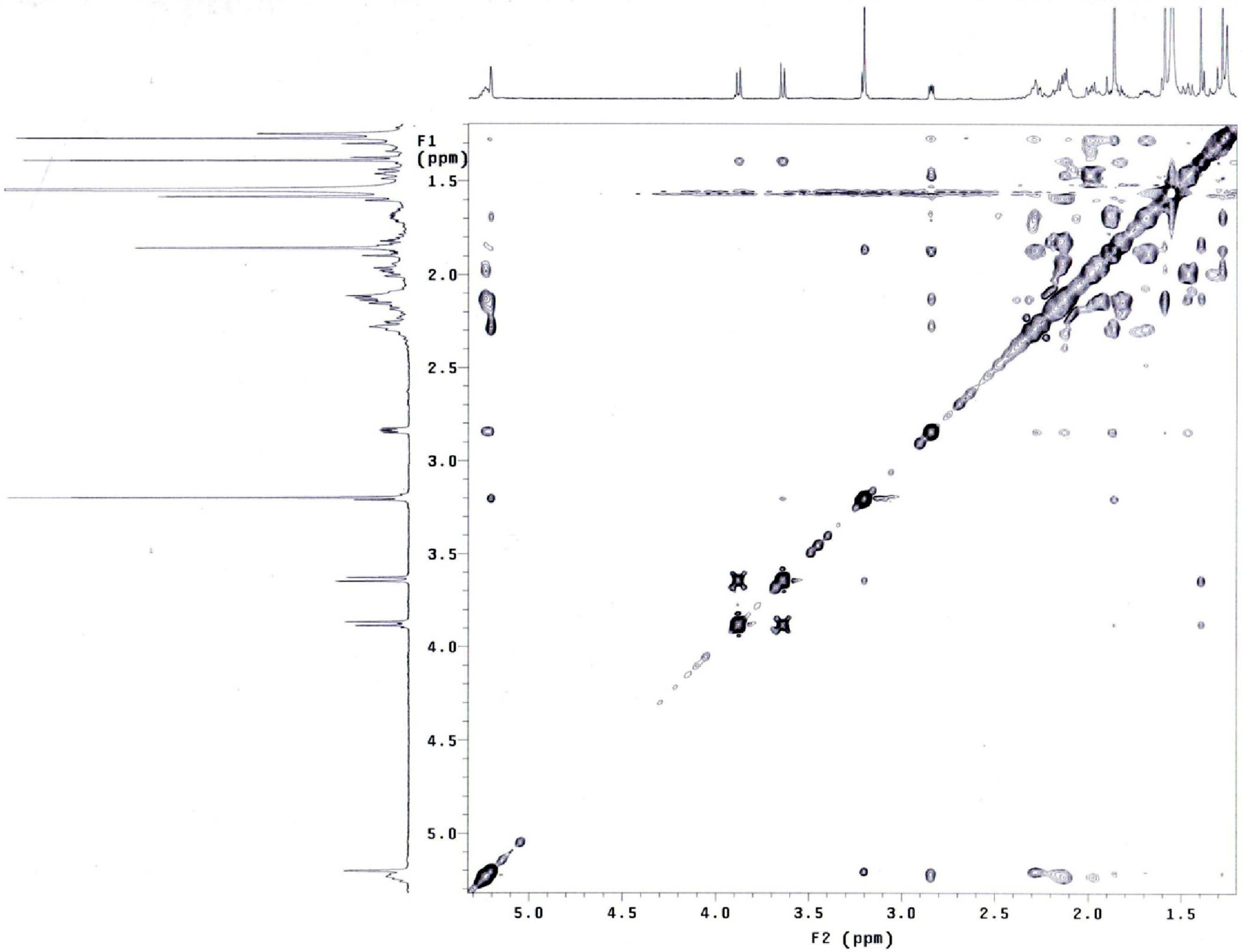

Figure S41. NOESY spectrum of **5** in $CDCl_3$

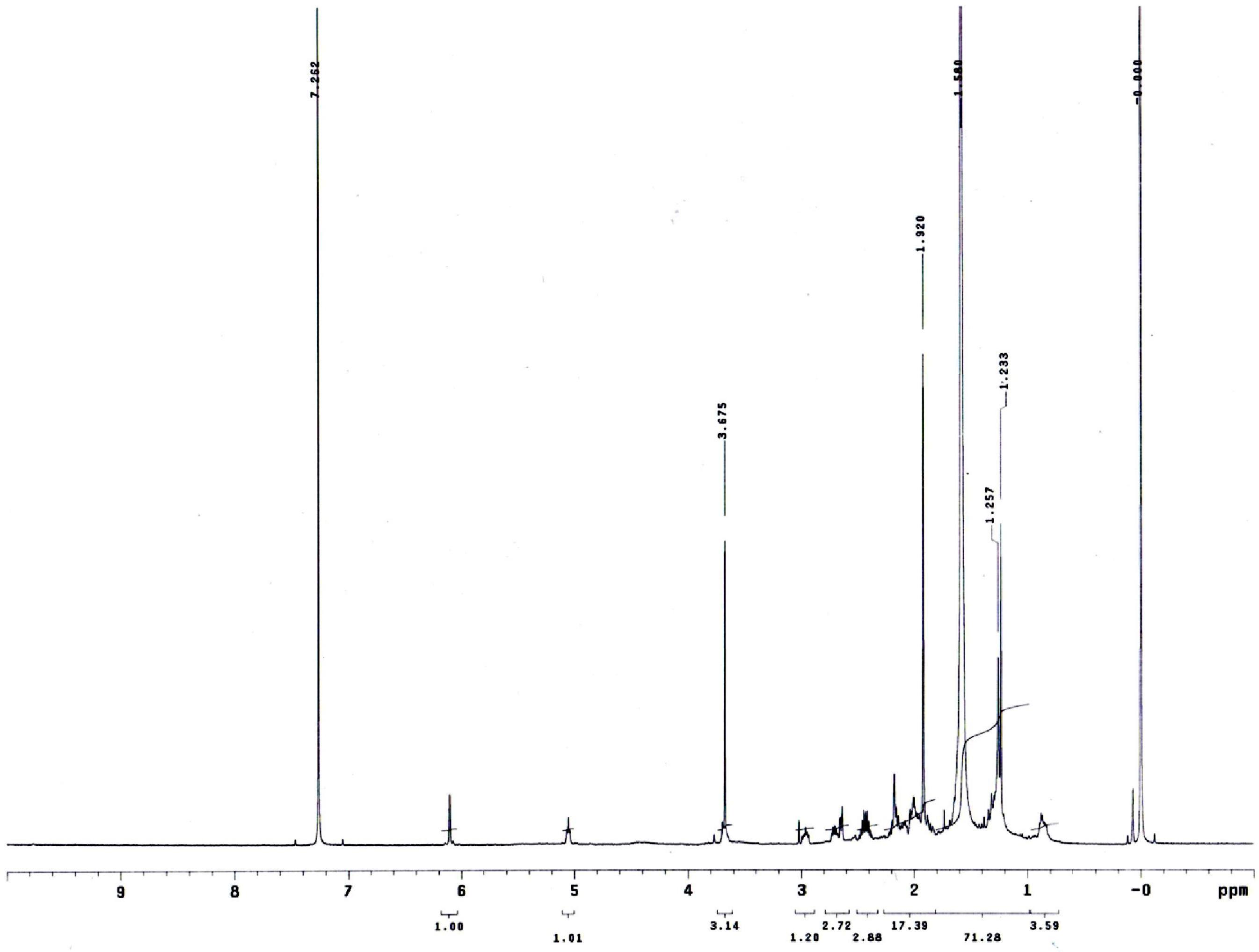

Figure S42. ^{1}H NMR spectrum of **6** in $CDCl_3$

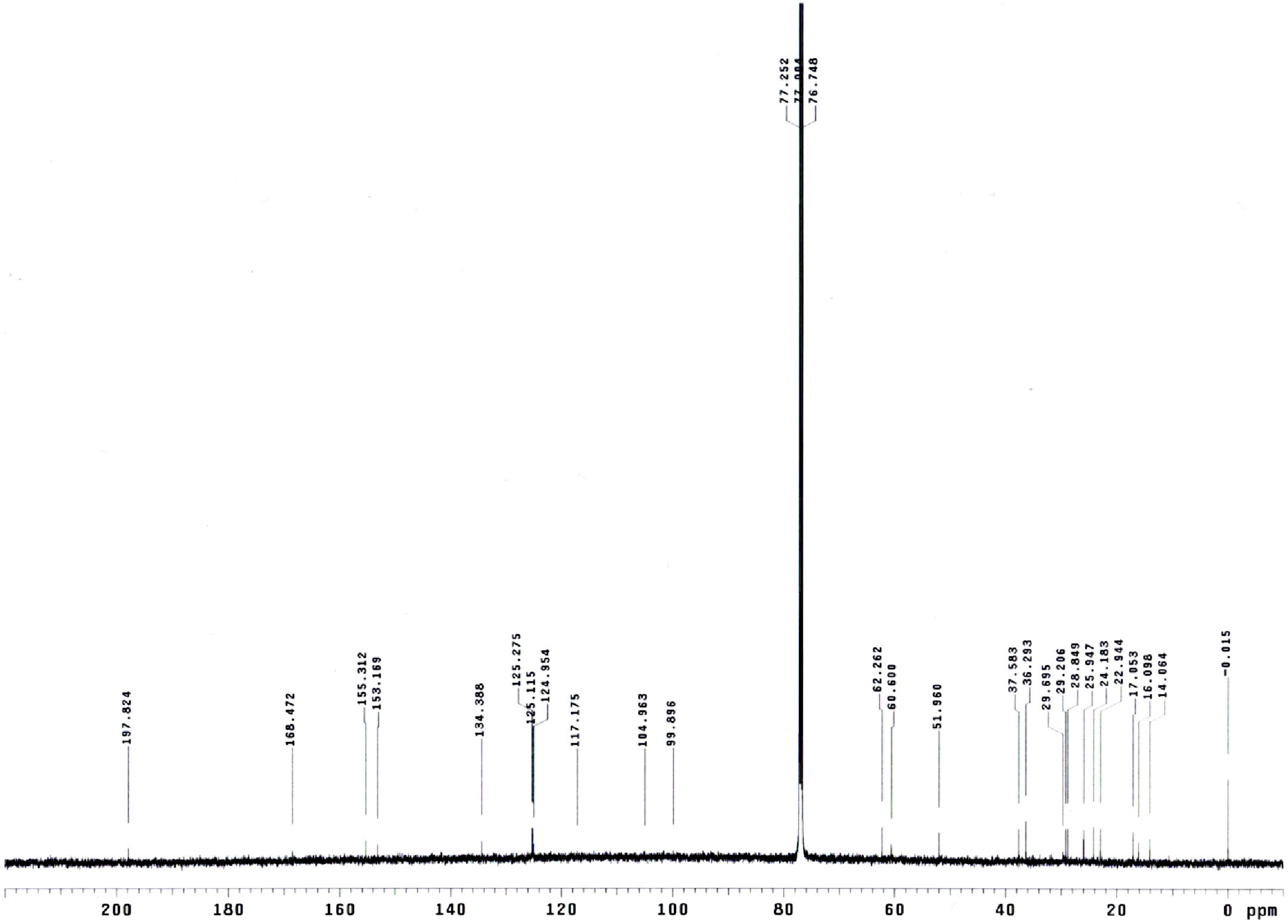

Figure S43. ^{13}C NMR spectrum of **6** in $CDCl_3$

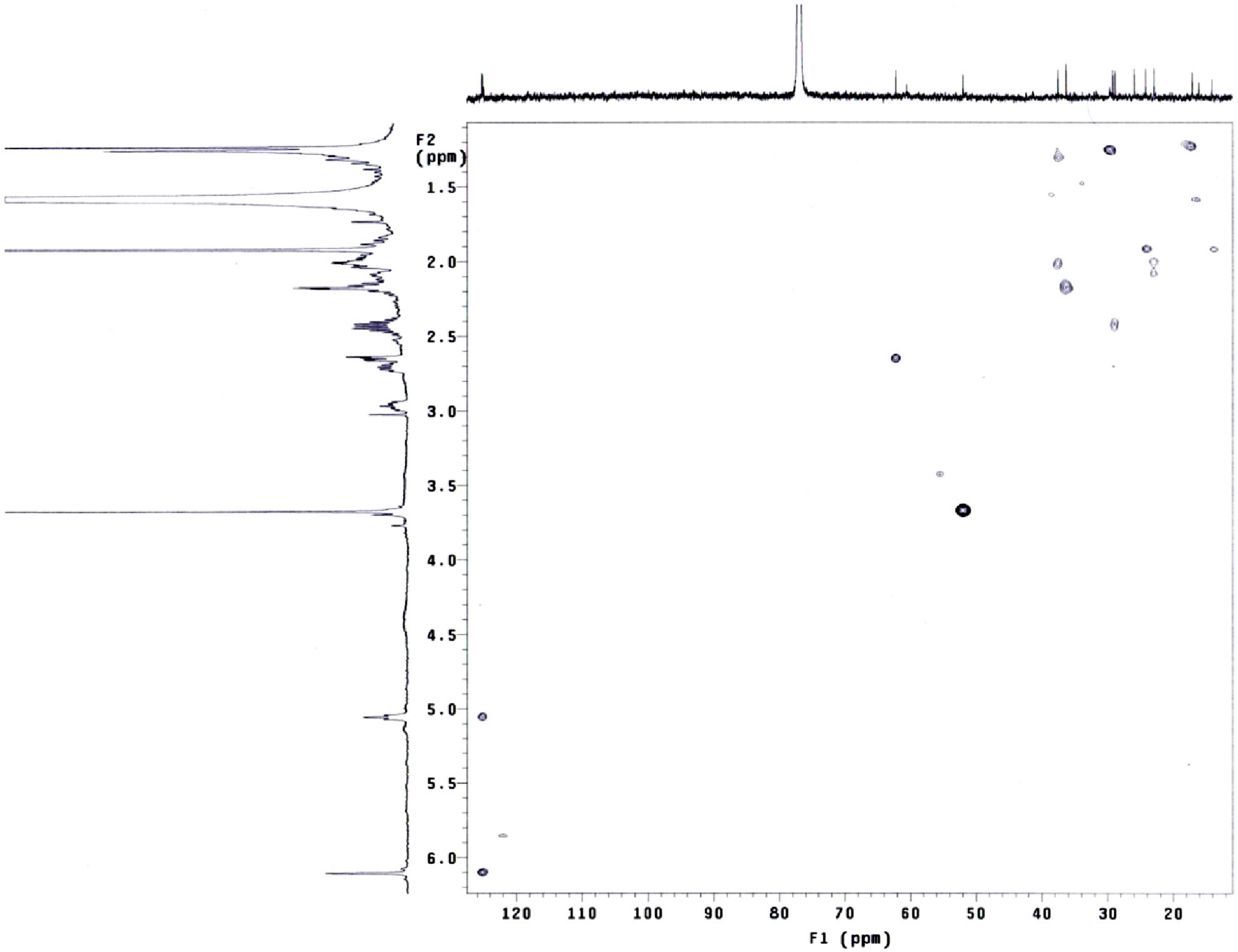

Figure S44. HMQC spectrum of **6** in $CDCl_3$

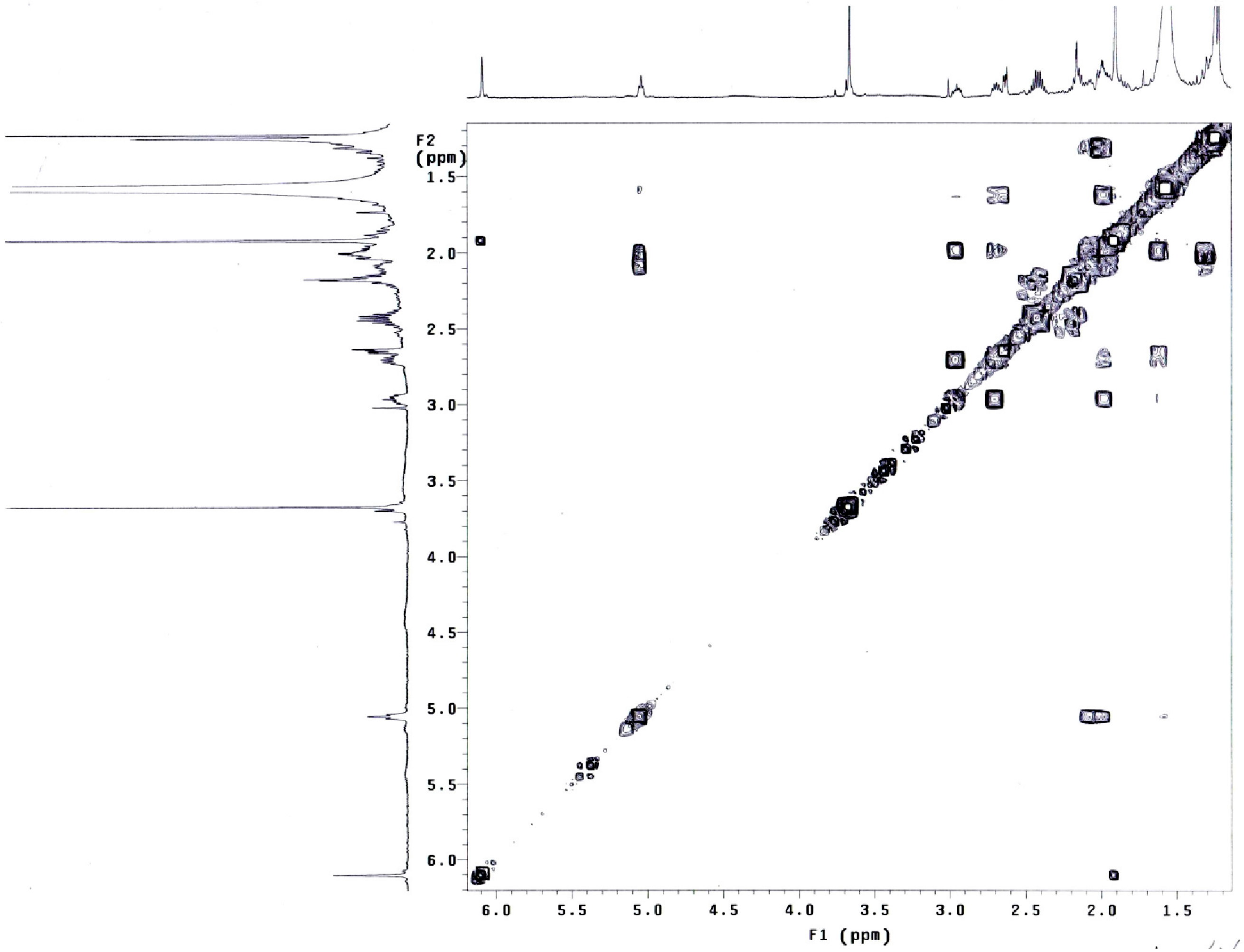

Figure S45. COSY spectrum of **6** in $CDCl_3$

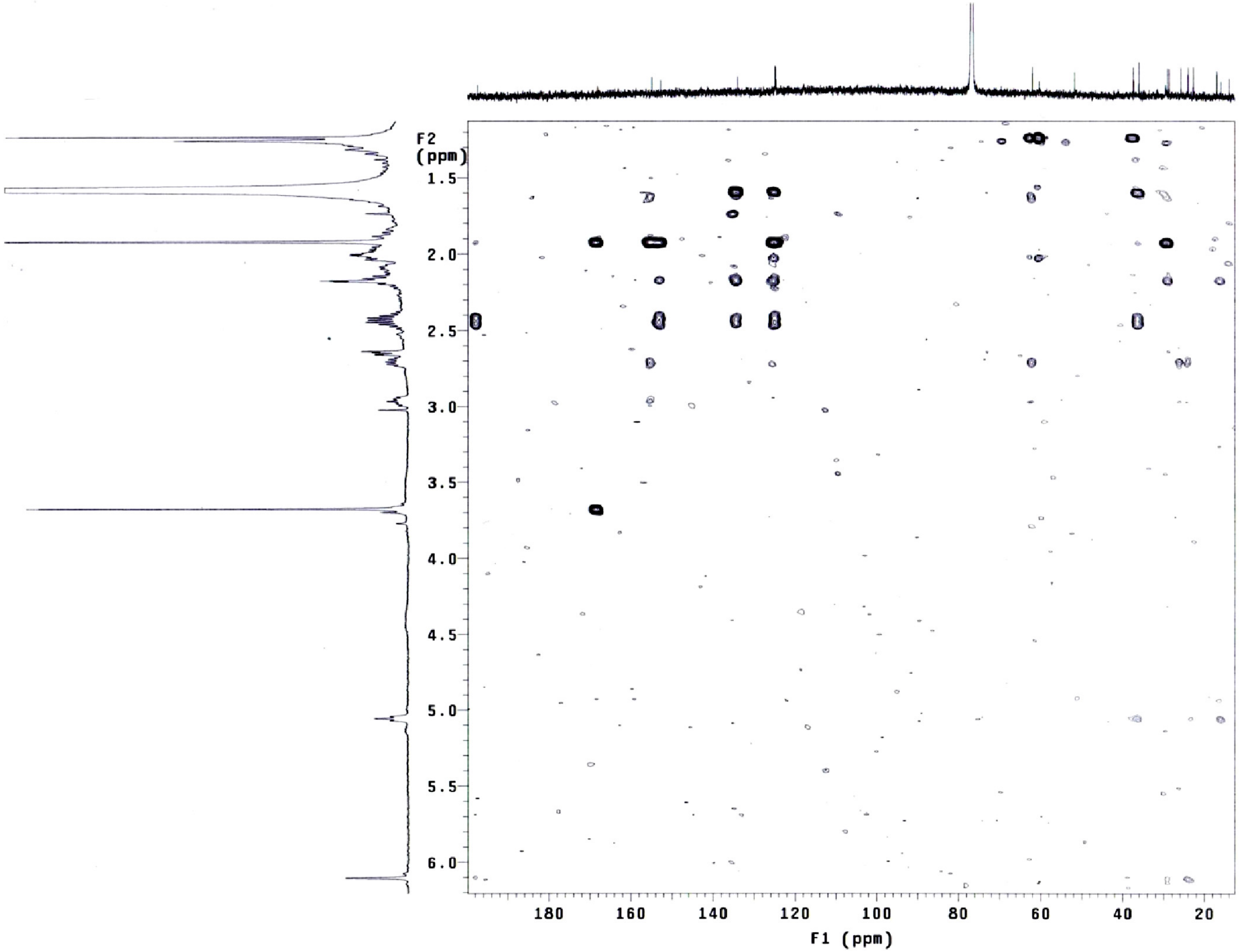

Figure S46. HMBC spectrum of **6** in $CDCl_3$

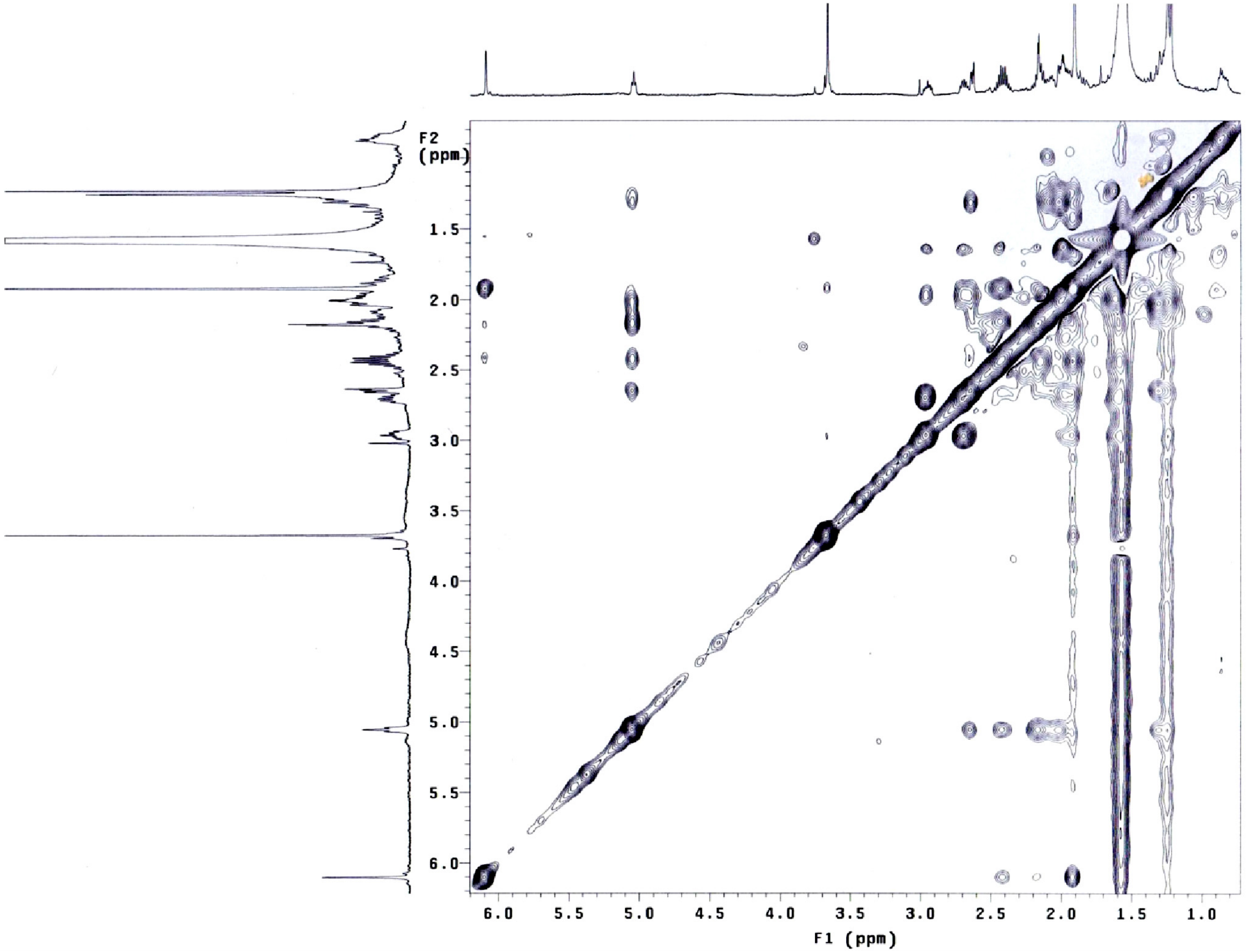

Figure S47. NOESY spectrum of **6** in $CDCl_3$
